# To what extent does the use of crosswalks instead of EQ-5D value sets impact reimbursement decisions?: a simulation study

**DOI:** 10.1007/s10198-022-01539-6

**Published:** 2022-11-13

**Authors:** Ângela Jornada Ben, Johanna M. van Dongen, Aureliano Paolo Finch, Mohamed El Alili, Judith E. Bosmans

**Affiliations:** 1 Department of Health Sciences, Faculty of Science, Vrije Universiteit Amsterdam, Amsterdam Public Health Research Institute, Van der Boechorststraat 7, 1081 BT Amsterdam, The Netherlands; 2 EuroQol Office, EuroQol Research Foundation, Marten Meesweg 107, 3068 AV Rotterdam, The Netherlands

**Keywords:** Quality of life, Methods, Cost–benefit analysis, EQ-5D, C18

## Abstract

**Purpose:**

Inconsistent results have been found on the impact of using crosswalks versus EQ-5D value sets on reimbursement decisions. We sought to further investigate this issue in a simulation study.

**Methods:**

Trial-based economic evaluation data were simulated for different conditions (depression, low back pain, osteoarthritis, cancer), severity levels (mild, moderate, severe), and effect sizes (small, medium, large). For all 36 scenarios, utilities were calculated using 3L and 5L value sets and crosswalks (3L to 5L and 5L to 3L crosswalks) for the Netherlands, the United States, and Japan. Utilities, quality-adjusted life years (QALYs), incremental QALYs, incremental cost-effectiveness ratios (ICERs), and probabilities of cost-effectiveness (pCE) obtained from values sets and crosswalks were compared.

**Results:**

Differences between value sets and crosswalks ranged from −0.33 to 0.13 for utilities, from −0.18 to 0.13 for QALYs, and from −0.01 to 0.08 for incremental QALYs, resulting in different ICERs. For small effect sizes, at a willingness-to-pay of €20,000/QALY, the largest pCE difference was found for moderate cancer between the Japanese 5L value set and 5L to 3L crosswalk (difference = 0.63). For medium effect sizes, the largest difference was found for mild cancer between the Japanese 3L value set and 3L to 5L crosswalk (difference = 0.06). For large effect sizes, the largest difference was found for mild osteoarthritis between the Japanese 3L value set and 3L to 5L crosswalk (difference = 0.08).

**Conclusion:**

The use of crosswalks instead of EQ-5D value sets can impact cost–utility outcomes to such an extent that this may influence reimbursement decisions.

**Supplementary Information:**

The online version contains supplementary material available at 10.1007/s10198-022-01539-6.

## Introduction

The EQ-5D is one of the most frequently used generic preference-based measures of health-related quality of life in economic evaluations worldwide [1, 2], as it is shown to be valid and responsive in multiple health conditions [3, 4] and cultural contexts [5]. It comprises a standardized descriptive system that describes health using five health dimensions (i.e., mobility, self-care, usual activities, pain/discomfort, and anxiety/depression). The original EQ-5D uses three severity levels per health dimension (EQ-5D-3L) to describe an individual’s health state, that is “no problems”, “some problems”, and “extreme problems” (further referred to as the EQ-5D-3L) [6]. To increase its sensitivity to changes within and between subjects’ health states and to reduce commonly observed ceiling effects, a 5-level version of the EQ-5D was developed (further referred to as the EQ-5D-5L) [7, 8]. The EQ-5D-5L describes health in terms of the same health dimensions, but uses five severity levels, that is “no problem”, “slight problems”, “moderate problems”, “severe problems”, and “extreme problems”. Literature has shown that the EQ-5D-5L has improved measurement properties compared with the EQ-5D-3L [9–11].

For Health Technology Assessment (HTA) purposes, EQ-5D health states are preferably scored using country-specific value sets. A value set includes a number of utilities assigned to each of the health states described by the EQ-5D [12]. These utilities typically indicate the general public’s preferences for a certain health state on a scale anchored at 0 (equaling death) and 1.0 (equaling full health). Utilities below zero are possible for health states that are considered to be worse than dead. By multiplying these utilities by the duration an individual spends in a certain health state, quality-adjusted life years (QALY) are calculated, which is the main effect outcome in cost–utility analyses [13].

In many countries, value sets are available for the EQ-5D-3L and/or the EQ-5D-5L. The use of national EQ-5D value sets is advised, if they have been produced according to the latest standard procedures (e.g., the EuroQol Valuation Technology—EQ-VT—protocol) [14, 15]. Otherwise, the country-specific value set may not be recommended by HTA agencies. For example, the National Institute for Health and Care Excellence (NICE) currently does not recommend using the EQ-5D-5L value set for England [16] due to methodological issues found in the initial version of the EQ-VT protocol [15, 17], but to use the mapping approach developed by Hernández-Alava and Pudney (2017) as an interim scoring method instead [18, 19]. In other situations, EQ-5D-3L or EQ-5D-5L data may have been collected in a clinical trial, while there is no national value set available at all for the country in which the trial was performed. In those cases, researchers may use a reference value set close to the socio-cultural context of application. It may also happen that a value set is only available for one of the EQ-5D versions (e.g., 3L), while data have been collected using the other version (e.g., 5L). In most of these cases, mapping approaches, such as crosswalks and copula mapping models, can be used to estimate utilities for the other instrument [20–22]. The most widely used mapping approach for HTA purposes [23] is the one of van Hout et al. (2012) [20], which estimates 5L utilities by mapping EQ-5D-5L to EQ-5D-3L (i.e., 5L to 3L crosswalk). An extension of this mapping approach was recently published by van Hout and Shaw (2021) [22], which estimates 3L utilities from mapping EQ-5D-3L to EQ-5D-5L (i.e., 3L to 5L crosswalk).

Given that healthcare decision-makers can be confronted with scientific evidence that is based on EQ-5D value sets or mapping approaches, guidance on choosing the most appropriate utility scoring method is urgently needed [23]. So far, literature suggests that EQ-5D scoring methods might result in different utility values, but inconsistent results have been found on the extent to which these differences affect differences in QALY between treatment groups (i.e., incremental QALY) and impact reimbursement decisions [18, 24–29]. Camacho et al. (2018), for example, concluded that the use of crosswalks instead of England 5L value sets may increase the likelihood of mental health interventions being cost-effective, while Ben et al. (2020) found that the probability of interventions for mental health and diabetes being cost-effective was not significantly affected using crosswalks compared to 5L value sets for England, the Netherlands, and Spain. Both studies, however, only used data of a small number of empirical studies (i.e., ≤ 5), which typically assessed a restricted number of health conditions and interventions with relatively small effect sizes.

This study was, therefore, conducted to further investigate the impact of using the 5L to 3L crosswalk compared to 5L value sets on cost–utility outcomes, and hence the possible impact on reimbursement decisions, in a broad range of simulated scenarios. These scenarios included a broader range of health conditions, particularly those that are associated with moderate and severe EQ-5D health states. Moreover, as a 3L to 5L crosswalk [22] has recently been published, we also decided to assess the impact of using the 3L to 5L crosswalk compared to the 3L value set in a wide range of simulated scenarios.

## Methods

To evaluate the impact of using crosswalks or EQ-5D value sets on cost–utility outcomes, trial-based economic evaluation data were simulated. In total, 36 different scenarios were simulated including four health conditions (i.e., depression, low back pain, osteoarthritis, and cancer), three severity levels (i.e., mild, moderate, and severe), and three treatment effect sizes (i.e., small, medium, and large). An overview of all scenarios can be found in Table 1. After using four EQ-5D scoring methods to estimate utilities (i.e., 3L and 5L value sets, 3L to 5L and 5L to 3L crosswalks) for the Netherlands (NL), the United States (US), and Japan (JP), cost–utility analyses were performed for all 36 scenarios. Finally, results obtained from the country-specific EQ-5D value sets and mapping approaches (also referred to as 3L to 5L and 5L to 3L crosswalks in this paper) were compared.

**Table 1:** Overview of simulated scenarios

Scenario	Patient population		Effect size
	Health condition	Severity level	
(1)	Depression	Mild	Small
(2)			Medium
(3)			Large
(4)		Moderate	Small
(5)			Medium
(6)			Large
(7)		Severe	Small
(8)			Medium
(9)			Large
(10)	Low back pain	Mild	Small
(11)			Medium
(12)			Large
(13)		Moderate	Small
(14)			Medium
(15)			Large
(16)		Severe	Small
(17)			Medium
(18)			Large
(19)	Osteoarthritis	Mild	Small
(20)			Medium
(21)			Large
(22)		Moderate	Small
(23)			Medium
(24)			Large
(25)		Severe	Small
(26)			Medium
(27)			Large
(28)	Cancer	Mild	Small
(29)			Medium
(30)			Large
(31)		Moderate	Small
(32)			Medium
(33)			Large
(34)		Severe	Small
(35)			Medium
(36)			Large

Third-six different scenarios were simulated including four different conditions (i.e., depression, low back pain, osteoarthritis, and cancer), three severity levels (i.e., mild, moderate, and severe health states), and three treatment effect sizes (i.e., small, medium, and large) for the Netherlands, the United States and Japan

### Data generation

Data from eight trial-based economic evaluations were used to inform the data generation process. These datasets contained EQ-5D-3L and EQ-5D-5L data of patients with depression [29, 30], low back pain [31, 32], osteoarthritis [33, 34], and cancer [35, 36].

First, the probabilities of observing the different EQ-5D-3L and EQ-5D-5L response levels per health dimension at baseline were extracted from the empirical data by treatment group (i.e., intervention and control). This was done for each EQ-5D version, health condition, and severity level separately. An overview of the cut-off scores [30, 37–44] used to classify patients as either having mild, moderate, or severe symptoms per health condition can be found in Appendix 1. Based on the extracted baseline probabilities, 150 baseline profiles were generated for a hypothetical intervention and control group. This was done using the EQ-5D simulation laboratory R package developed by Parkin et al., which is provided the EuroQol Foundation for simulation studies [45]. This package allows researchers to generate datasets with EQ-5D health states (e.g., 12,312) of artificial patients, based on pre-specified probabilities of observing the specific response levels within the dimensions. In the current study, these probabilities were based on empirical datasets [29–36].

Subsequently, 150 follow-up profiles were generated by treatment group for each EQ-5D version, health condition, and severity level separately. This was done using a matrix of transition probabilities which were also based on the empirical datasets [29–36]. These transitions probabilities were then tweaked to obtain small, medium, and large treatment effect sizes. The magnitude of the effect sizes was based on Cohen’s *d* (0.1–0.3 small, 0.5–0.7 medium, and > 0.8 large) [46].

Finally, baseline characteristics (i.e., age and gender) and follow-up costs were generated and linked to the health profiles using the simstudy R package [47]. Age was generated from a uniform integer distribution including minimum and maximum values of 25 and 75 years, respectively. The proportion of male subjects was randomly generated from a binary distribution with a mean of 0.19. Follow-up costs were generated from a gamma distribution with a mean of €2000, a “true value” of the mean difference between treatment groups of €250, and a variance of 1. Please note that “true value” means that in 95% of the cases, €250 is included in the 95% confidence interval of the generated cost difference. A negative correlation between costs and QALYs was implemented (*r* ≈ −0.10). This means that high costs are associated with lower QALYs and vice-versa. The R script for the data generation can be found at GitHub or in Appendix 2.

### Scoring methods

Utilities were estimated using four EQ-5D scoring methods: 3L value set, 5L value set, 3L to 5L crosswalk [22], and 5L to 3L crosswalk [20]. For both versions of the EQ-5D, utilities were calculated for NL, US, and JP using the equation 5d R package [48]. These three countries were chosen, because they differ considerably in terms of the utility decrements assigned to the different health dimensions of the EQ-5D. For example, for the EQ-5D-3L, the decrement of being “confined to bed” (response level 3 on the mobility dimension) is 0.161 in NL, 0.490 in US and 0.418 in JP. Another example is the decrement of being “extremely anxious or depressed” of the EQ-5D-5L (response level 5 in the anxiety/depression dimension), which is 0.421 in NL, 0.340 in US, and 0.197 in JP. Subsequently, 3L to 5L and 5L to 3L crosswalked utilities for the three countries were estimated using the mapping approaches available on the EuroQol website: https://euroqol.org/support/analysis-tools/cross-walk/. These mapping approaches were chosen as they are the ones mostly used in practice [23].

### Analysis

#### Utilities and QALYs

For all scenarios and countries, the utilities distribution of the two simulated measurement points (i.e., baseline and follow-up) were assessed using Kernel density histograms. Additionally, mean utilities at baseline and mean QALYs (estimated using the area under the curve method) [13] as well as their respective standard deviations and ranges were described. For the EQ-5D-3L, utilities and QALYs estimated using country-specific 3L value sets and their respective 3L to 5L crosswalks were described. For the EQ-5D-5L, utilities and QALYs estimated using the country-specific 5L value sets and their respective 5L to 3L crosswalks were described. Differences in utilities and QALYs between EQ-5D value sets and mapping approaches were compared using paired *t* tests and their corresponding 95% confidence intervals (95%CI) were described per country. To explore whether the differences between scoring methods were clinically relevant, a minimally clinically important difference of 0.074 was used as a threshold [49].

#### Cost–utility analysis

Using QALYs derived from the four EQ-5D scoring methods, cost–utility analyses were performed for all 36 scenarios per country. Incremental QALYs and costs between treatment groups and surrounding 95%CIs were estimated using seemingly unrelated regression analyses [50]. Incremental cost-effectiveness ratios (ICERs) were calculated by dividing incremental costs by incremental QALYs. Bias-corrected and accelerated bootstrapping with 2000 replications was used to estimate statistical uncertainty surrounding the ICERs [51, 52]. The distribution of the bootstrapped estimates was presented in the cost-effectiveness plane (CE-plane) [51]. The probability of an intervention being cost-effective compared to control was estimated using the Incremental Net Benefit (INB) approach, where the probability of cost-effectiveness was estimated as the probability that INB > 0 for every value of the willingness-to-pay (WTP) threshold (i.e., €0, €20,000, €30,000, and €50,000 per QALY) [53]. In this study, an intervention was considered cost-effective if the probability of cost-effectiveness at a specific WTP threshold was ≥ 0.80. Cost–utility analysis outcomes were descriptively compared across scoring methods (i.e., between EQ-5D value sets and crosswalks). Data analyses were performed in StataSE 16® (StataCorp LP, CollegeStation, TX, US).

## Results

### Utilities

**Fig. 1:**
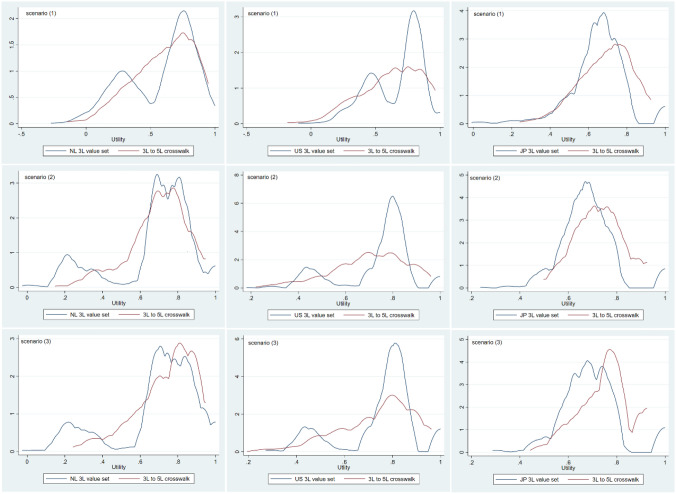
Utility distribution EQ-5D-3L value sets and 3L to 5L crosswalks for the Netherlands (NL), the United States (US), and Japan (JP). Scenario (1): mild depression and small treatment effect size. Scenario (2): mild depression and medium treatment effect size. Scenario (3): mild depression and large treatment effect size

**Fig. 2:**
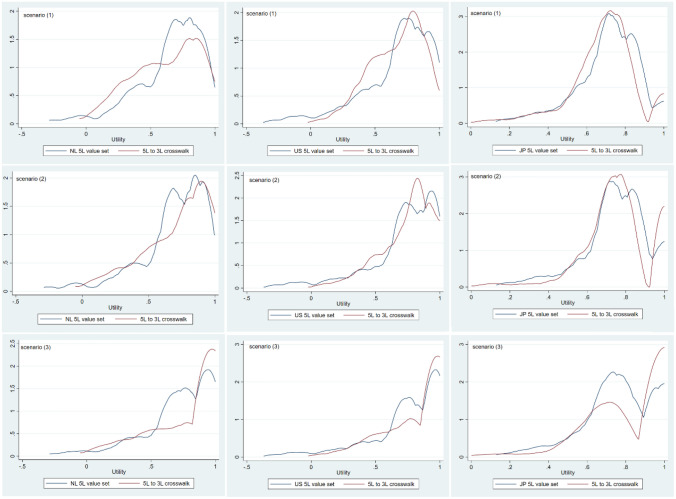
Utility distribution EQ-5D-5L value sets and 5L to 3L crosswalks for the Netherlands (NL), the United States (US), and Japan (JP). Scenario (1): mild depression and small treatment effect size. Scenario (2): mild depression and medium treatment effect size. Scenario (3): mild depression and large treatment effect size

The distribution of utilities at baseline estimated by the crosswalks differed in all scenarios and countries from those estimated by 3L and 5L value sets. Differences in utilities distributions were more pronounced for the EQ-5D-3L than for the EQ-5D-5L. An example of such differences is shown in Figs. 1 and 2. Detailed information can be found in Appendix 3.

Differences in baseline utilities between EQ-5D value sets and crosswalks ranged from −0.33 for the severe low back pain scenario (i.e., between the US 5L value set and 5L to 3L crosswalk, Table 3) to 0.13 for severe cancer scenario (i.e., between the US 3L value set and 3L to 5L crosswalk, Table 2). Baseline utilities estimated by EQ-5D value sets differed statistically significantly from those estimated using crosswalks in all health conditions and severity levels in the investigated countries, except for the Dutch EQ-5D-3L estimates for severe osteoarthritis (difference = 0.001, IC 95% −0.01; 0.01, Table 2) and for the Japanese EQ-5D-5L estimates for moderate depression (difference = −0.002, IC 95% −0.01; 0.003, Table 3).

**Table 2:** Differences in utilities estimated by 3L value sets and 3L to 5L crosswalks

Country	Scoring method	Patient population	Mean utilities (SD)	Min	Max	3L vs–3L to 5L cw (95% CI)
NL	3L value set	Mild depression	0.63 (0.12)	−0.03	1	0.01 (0.003; 0.02)
	3L to 5L crosswalk		0.62 (0.14)	0.07	0.95	
US	3L value set		0.71 (0.15)	0.27	1	**0.08 (0.07; 0.08)**
	3L to 5L crosswalk		0.63 (0.15)	0.11	0.96	
JP	3L value set		0.65 (0.08)	0.42	1	−0.02 (−0.03; −0.02)
	3L to 5L crosswalk		0.67 (0.09)	0.42	0.92	
NL	3L value set	Moderate depression	0.57 (0.22)	−0.07	1	0.01 (0.001; 0.02)
	3L to 5L crosswalk		0.56 (0.15)	−0.03	0.95	
US	3L value set		0.67 (0.16)	0.21	1	**0.09 (0.09; 0.10)**
	3L to 5L crosswalk		0.58 (0.16)	−0.02	0.96	
JP	3L value set		0.62 (0.10)	0.15	1	−0.02 (−0.02; −0.01)
	3L to 5L crosswalk		0.64 (0.09)	0.35	0.92	
NL	3L value set	Severe depression	0.30 (0.24)	−0.23	0.80	**−0.07 (−0.08; −0.06)**
	3L to 5L crosswalk		0.37(0.19)	−0.15	0.80	
US	3L value set		0.47 (0.19)	−0.01	0.84	**0.09 (0.08; 0.09)**
	3L to 5L crosswalk		0.38 (0.20)	−0.18	0.86	
JP	3L value set		0.50 (0.16)	−0.01	0.78	−0.03 (−0.04; −0.02)
	3L to 5L crosswalk		0.53 (0.11)	0.24	0.81	
NL	3L value set	Mild low back pain	0.79 (0.08)	0.43	1	0.06 (0.05; 0.06)
	3L to 5L crosswalk		0.73 (0.07)	0.50	0.95	
US	3L value set		0.79 (0.06)	0.51	1	0.06 (0.05; 0.07)
	3L to 5L crosswalk		0.73 (0.10)	0.38	0.96	
JP	3L value set		0.70 (0.06)	0.51	1	−0.04 (−0.04; −0.04)
	3L to 5L crosswalk		0.74 (0.07)	0.53	0.92	
NL	3L value set	Moderate low back pain	0.68 (0.19)	0.09	1	0.03 (0.02; 0.04)
	3L to 5L crosswalk		0.65 (0.13)	0.24	0.95	
US	3L value set		0.72 (0.14)	0.31	1	**0.09 (0.08; 0.09)**
	3L to 5L crosswalk		0.63 (0.14)	0.19	0.96	
JP	3L value set		0.65 (0.08)	0.42	1	−0.03 (−0.03; −0.03)
	3L to 5L crosswalk		0.68 (0.09)	0.44	0.92	
NL	3L value set	Severe low back pain	0.43 (0.25)	−0.11	0.81	−0.04 (−0.05; −0.02)
	3L to 5L crosswalk		0.47 (0.15)	0.01	0.74	
US	3L value set		0.54 (0.18)	0.08	0.82	**0.11 (0.09; 0.11)**
	3L to 5L crosswalk		0.43 (0.16)	0.001	0.77	
JP	3L value set		0.54 (0.09)	0.05	0.72	−0.04 (−0.04; −0.03)
	3L to 5L crosswalk		0.58 (0.08)	0.33	0.77	
NL	3L value set	Mild osteoarthritis	0.80 (0.09)	0.37	1	0.05 (0.05; 0.06)
	3L to 5L crosswalk		0.75 (0.08)	0.55	0.95	
US	3L value set		0.80 (0.09)	0.36	1	0.06 (0.05; 0.07)
	3L to 5L crosswalk		0.74 (0.10)	0.46	0.96	
JP	3L value set		0.71 (0.10)	0.30	1	−0.04 (−0.04; −0.03)
	3L to 5L crosswalk		0.76 (0.08)	0.55	0.92	
NL	3L value set	Moderate osteoarthritis	0.76 (0.09)	0.33	1	**0.08 (0.07; 0.08)**
	3L to 5L crosswalk		0.68 (0.07)	0.50	0.95	
US	3L value set		0.77 (0.07)	0.45	1	**0.11 (0.11; 0.12)**
	3L to 5L crosswalk		0.65 (0.10)	0.38	0.96	
JP	3L value set		0.66 (0.05)	0.51	1	−0.02 (−0.02; −0.02)
	3L to 5L crosswalk		0.68 (0.07)	0.52	0.92	
NL	3L value set	Severe osteoarthritis	0.52 (0.26)	−0.03	0.89	0.001 (−0.01; 0.01)
	3L to 5L crosswalk		0.52 (0.16)	0.07	0.85	
US	3L value set		0.61 (0.18)	0.27	0.85	**0.12 (0.11; 0.12)**
	3L to 5L crosswalk		0.49 (0.15)	0.09	0.83	
JP	3L value set		0.58 (0.08)	0.38	0.77	−0.02 (−0.02; −0.02)
	3L to 5L crosswalk		0.60 (0.78)	0.37	0.79	
NL	3L value set	Mild cancer	0.92 (0.09)	0.69	1	0.05 (0.04; 0.05)
	3L to 5L crosswalk		0.87 (0.08)	0.62	0.95	
US	3L value set		0.91 (0.08)	0.77	1	0.02 (0.02; 0.03)
	3L to 5L crosswalk		0.89 (0.08)	0.62	0.96	
JP	3L value set		0.88 (0.12)	0.65	1	0.02 (0.01; 0.02)
	3L to 5L crosswalk		0.86 (0.07)	0.66	0.92	
NL	3L value set	Moderate cancer	0.73 (0.15)	0.21	1	0.03 (0.03; 0.04)
	3L to 5L crosswalk		0.70 (0.11)	0.38	0.95	
US	3L value set		0.76 (0.11)	0.42	1	0.06 (0.05; 0.07)
	3L to 5L crosswalk		0.70 (0.13)	0.39	0.96	
JP	3L value set		0.69 (0.09)	0.45	1	−0.03 (−0.03; −0.02)
	3L to 5L crosswalk		0.72 (0.09)	0.50	0.92	
NL	3L value set	Severe cancer	0.55 (0.40)	−0.33	1	0.04 (0.03; 0.04)
	3L to 5L crosswalk		0.51 (0.36)	−0.31	1	
US	3L value set		0.62 (0.34)	−0.11	1	**0.13 (0.12; 0.14)**
	3L to 5L crosswalk		0.49 (0.40)	−0.42	0.96	
JP	3L value set		0.56 (0.34)	−0.11	1	−0.04 (−0.05; −0.02)
	3L to 5L crosswalk		0.60 (0.24)	0.10	0.92	

*3Lvs* EQ-5D-3L value set; *cw* crosswalk; *NL* the Netherlands; *US* United States; *JP* Japan; *CI* confidence interval

Differences in utilities between 3L value set and 3L to 5L crosswalk ≥ 0.074 (i.e., the minimally clinically important difference) are highlighted in bold

For the Netherlands, differences were clinically relevant in 2 out of 12 patient populations (i.e., 17%), for the United States in 8 out of 12 (i.e., 67%), for Japan no clinically relevant differences were found. Note that only 12 possible comparisons could be done as no treatment effect was simulated at baseline. That is, four health conditions times three severity levels, also referred to as patient population

**Table 3:** Differences in utilities estimated by 5L value sets and 5L to 3L crosswalks

Country	Scoring method	Patient population	Mean utilities (SD)	Min	Max	5Lvs–5L to 3L cw (95% CI)
NL	5L value set	Mild depression	0.66 (0.26)	−0.29	1	−0.03 (−0.03; −0.01)
	5L to 3L crosswalk		0.69 (0.20)	0.003	1	
US	5L value set		0.70 (0.28)	−0.37	1	−0.06 (−0.08; −0.05)
	5L to 3L crosswalk		0.76 (0.15)	0.20	1	
JP	5L value set		0.71 (0.16)	0.13	1	0.01 (0.005; 0.01)
	5L to 3L crosswalk		0.70 (0.13)	0.30	1	
NL	5L value set	Moderate depression	0.58 (0.29)	−0.41	1	−0.04 (−0.05; −0.03)
	5L to 3L crosswalk		0.62 (0.23)	−0.16	1	
US	5L value set		0.62 (0.31)	−0.45	1	**−0.10 (−0.12; −0.08)**
	5L to 3L crosswalk		0.72 (0.17)	0.13	1	
JP	5L value set		0.67 (0.17)	0.08	1	−0.002 (−0.01; 0.003)
	5L to 3L crosswalk		0.67 (0.13)	0.24	1	
NL	5L value set	Severe depression	0.37 (0.37)	−0.41	1	**−0.08 (−0.10; −0.08)**
	5L to 3L crosswalk		0.45 (0.28)	−0.26	1	
US	5L value set		0.40 (0.40)	−0.45	1	−0.20 (−0.22; −0.18)
	5L to 3L crosswalk		0.60 (0.22)	−0.04	1	
JP	5L value set		0.55 (0.21)	0.07	1	−0.04 (−0.04; −0.03)
	5L to 3L crosswalk		0.59 (0.17)	−0.06	1	
NL	5L value set	Mild low back pain	0.45 (0.34)	−0.18	0.80	**−0.10 (−0.11; −0.09)**
	5L to 3L crosswalk		0.55 (0.22)	0.17	0.81	
US	5L value set		0.39 (0.35)	−0.22	0.78	**−0.22 (−0.24; −0.20)**
	5L to 3L crosswalk		0.61 (0.16)	0.35	0.81	
JP	5L value set		0.52 (0.18)	0.24	0.76	−0.03 (−0.04; −0.04)
	5L to 3L crosswalk		0.55 (0.10)	0.41	0.69	
NL	5L value set	Moderate low back pain	0.42 (0.31)	−0.28	0.86	**−0.10 (−0.12; −0.09)**
	5L to 3L crosswalk		0.52 (0.20)	−0.11	0.84	
US	5L value set		0.37 (0.32)	−0.32	0.90	**−0.22 (−0.24; −0.20)**
	5L to 3L crosswalk		0.59 (0.15)	0.06	0.83	
JP	5L value set		0.52 (0.18)	0.13	0.87	−0.03 (−0.04; −0.02)
	5L to 3L crosswalk		0.54 (0.10)	0.005	0.77	
NL	5L value set	Severe low back pain	0.24 (0.22)	−0.08	0.75	**−0.18 (−0.18; −0.16)**
	5L to 3L crosswalk		0.42 (0.13)	0.27	0.72	
US	5L value set		0.18 (0.23)	−0.15	0.65	**−0.33 (−0.34; −0.31)**
	5L to 3L crosswalk		0.51 (0.09)	0.39	0.72	
JP	5L value set		0.45 (0.15)	0.25	0.71	−0.05 (−0.06; −0.04)
	5L to 3L crosswalk		0.50 (0.07)	0.42	0.63	
NL	5L value set	Mild osteoarthritis	0.82 (0.17)	0.05	1	−0.001 (−0.01; 0.004)
	5L to 3L crosswalk		0.82 (0.13)	0.32	1	
US	5L value set		0.82 (0.20)	−0.02	1	−0.006 (−0.02; 0.01)
	5L to 3L crosswalk		0.83 (0.11)	0.44	1	
JP	5L value set		0.82 (0.15)	0.33	1	0.06 (0.05; 0.06)
	5L to 3L crosswalk		0.76 (0.13)	0.44	1	
NL	5L value set	Moderate osteoarthritis	0.78 (0.13)	−0.08	1	−0.002 (−0.01; 0.002)
	5L to 3L crosswalk		0.78 (0.10)	0.20	1	
US	5L value set		0.75 (0.15)	−0.06	1	−0.03 (−0.04; −0.03)
	5L to 3L crosswalk		0.79 (0.09)	0.38	1	
JP	5L value set		0.75 (0.12)	0.30	1	0.06 (0.05; 0.06)
	5L to 3L crosswalk		0.69 (0.10)	0.43	1	
NL	5L value set	Severe osteoarthritis	0.59 (0.35)	−0.38	0.89	−0.05 (−0.07; −0.04)
	5L to 3L crosswalk		0.65 (0.27)	−0.23	0.87	
US	5L value set		0.55 (0.38)	−0.55	0.94	**−0.13 (−0.15; −0.11)**
	5L to 3L crosswalk		0.68 (0.23)	−0.07	0.86	
JP	5L value set		0.63 (0.23)	−0.001	0.90	0.03 (0.03; 0.04)
	5L to 3L crosswalk		0.60 (0.19)	−0.09	0.81	
NL	5L value set	Mild cancer	0.85 (0.20)	−0.10	1	−0.01 (−0.01; −0.003)
	5L to 3L crosswalk		0.86 (0.18)	−0.02	1	
US	5L value set		0.85 (0.22)	−0.18	1	−0.03 (−0.04; −0.02)
	5L to 3L crosswalk		0.88 (0.15)	0.23	1	
JP	5L value set		0.85 (0.18)	0.20	1	0.01 (0.01; 0.02)
	5L to 3L crosswalk		0.83 (0.18)	0.31	1	
NL	5L value set	Moderate cancer	0.76 (0.26)	−0.34	1	−0.03 (−0.03; −0.02)
	5L to 3L crosswalk		0.79 (0.20)	−0.06	1	
US	5L value set		0.75 (0.28)	−0.42	1	−0.06 (−0.07; −0.05)
	5L to 3L crosswalk		0.81 (0.16)	0.18	1	
JP	5L value set		0.76 (0.21)	0.10	1	0.01 (0.01; 0.02)
	5L to 3L crosswalk		0.75 (0.19)	0.27	1	
NL	5L value set	Severe cancer	0.55 (0.50)	−0.45	1	−0.06 (−0.08; −0.05)
	5L to 3L crosswalk		0.61 (0.41)	−0.33	1	
US	5L value set		0.52 (0.53)	−0.57	1	**−0.16 (−0.19; −0.14)**
	5L to 3L crosswalk		0.69 (0.32)	−0.11	1	
JP	5L value set		0.65 (0.33)	−0.02	1	−0.01 (−0.02; −0.005)
	5L to 3L crosswalk		0.66 (0.30)	−0.11	1	

*5Lvs* EQ-5D-5L value set; *cw* crosswalk; *NL* the Netherlands; *US* United States; *JP* Japan; *NL* the Netherlands; *US* United States; *JP* Japan; *CI* confidence interval

Differences in utilities between 5L value set and 5L to 3L crosswalk ≥ 0.074 (i.e., the minimally clinically important difference) are highlighted in bold. For the Netherlands, differences were clinically relevant in 4 out of 12 patient populations (i.e., 33%), for the United States in 6 out of 12 (i.e., 50%), for Japan no clinically relevant differences were found. Note that only 12 possible comparisons could be done as no treatment effect was simulated at baseline. That is, four health conditions times three severity levels, also referred to as patient population

No clinically relevant differences between the Japanese 3L value set and 3L to 5L crosswalk were found, whereas clinically relevant differences were found in 17% of the 12 possible comparisons between the Dutch 3L value set and 3L to 5L crosswalk and in 67% of those between the US value set and 3L to 5L crosswalk (Table 2). No clinically relevant differences between the Japanese 5L value set and 5L to 3L crosswalk were found, whereas between the Dutch and US value sets and their respective 5L to 3L crosswalks, clinically relevant differences were found in 33% and 50% of the comparisons, respectively (Table 3).

### QALYs

Differences in QALYs between EQ-5D value sets and crosswalks ranged from −0.18 (i.e., between the US 5L value set and 5L to 3L crosswalk, Table 4, scenario 16) to 0.13 (i.e., between the US 3L value set and 3L to 5L crosswalk, Table 4, scenario 26). QALYs statistically significantly differed between EQ-5D value sets and crosswalks in all 36 scenarios for the three countries. No clinically relevant differences between the 3L value set and 3L to 5L crosswalk were found for Japan and the Netherlands, whereas differences were clinically relevant in 14% of scenarios for the US. Clinically relevant differences between the 5L value set and 5L to 3L crosswalk were found in 8%, 25%, and 50% of scenarios, for the Netherlands, Japan, and the United States, respectively.

**Table 4:** Overview of differences in QALY between EQ-5D value sets and crosswalks

Country	Scoring method	Scenario	Patient population	Effect size	QALYs (SD)	Min	Max	QALY–QALY cw (95% CI)
NL	3L value set	(7)	Severe depression	Small	0.44 (0.20)	−0.07	0.88	**−0.07 (−0.07; −0.06)**
	3L to 5L crosswalk				0.51 (0.15)	0.06	0.84	
US	3L value set				0.56 (0.15)	0.20	0.91	0.04 (0.04; 0.05)
	3L to 5L crosswalk				0.52 (0.15)	0.10	0.85	
JP	3L value set				0.58 (0.12)	0.25	0.91	−0.05 (−0.05; −0.04)
	3L to 5L crosswalk				0.63 (0.08)	0.41	0.81	
NL	3L value set	(26)	Severe osteoarthritis	Medium	0.28 (0.13)	−0.03	0.46	−0.05 (−0.06; −0.05)
	3L to 5L crosswalk				0.33 (0.08)	0.03	0.50	
US	3L value set				0.45 (0.09)	0.24	0.60	**0.13 (0.12; 0.13)**
	3L to 5L crosswalk				0.33 (0.08)	−0.02	0.53	
JP	3L value set				0.52 (0.09)	0.30	0.65	0.002 (−0.01; 0.002)
	3L to 5L crosswalk				0.52 (0.04)	0.33	0.63	
NL	5L value set	(16)	Severe low back pain	Small	0.42 (0.18)	−0.06	0.87	−0.07 (−0.08; −0.07)
	5L to 3L crosswalk				0.49 (0.15)	0.12	0.86	
US	5L value set				0.39 (0.18)	−0.15	0.82	**−0.18 (−0.19; −0.18)**
	5L to 3L crosswalk				0.57 (0.12)	0.23	0.86	
JP	5L value set				0.43 (0.15)	0.05	0.82	−0.15 (−0.15; −0.14)
	5L to 3L crosswalk				0.58 (0.10)	0.21	0.81	
NL	5L value set	(22)	Moderate osteoarthritis	Small	0.71 (0.15)	0.13	0.96	0.02 (0.02; 0.02)
	5L to 3L crosswalk				0.69 (0.14)	0.25	0.94	
US	5L value set				0.69 (0.15)	0.13	0.97	−0.03 (−0.03; −0.02)
	5L to 3L crosswalk				0.72 (0.11)	0.37	0.93	
JP	5L value set				0.73 (0.12)	0.22	0.97	**0.05 (0.04; 0.05)**
	5L to 3L crosswalk				0.68 (0.10)	0.34	0.91	

*3Lvs* EQ-5D-3L value set; *5Lvs* EQ-5D-5L value set; *cw* crosswalk; *NL* the Netherlands; *US* United States; *JP* Japan; *CI* confidence interval

Scenario 7 represents the lowest difference in QALYs between 3L value sets and 3L to 5L crosswalks across all scenarios (i.e., −0.07 in bold). Scenario 26 represents the largest difference in incremental QALYs between 3L value sets and 3L to 5L crosswalks across all scenarios (i.e., 0.13 in bold)

Scenario 16 represents the lowest difference in QALYs between 5L value sets and 5L to 3L crosswalks across all scenarios (i.e., −0.18 in bold). Scenario 22 represents the largest difference in incremental QALYs between 5L value sets and 5L to 3L crosswalks across all scenarios (i.e., 0.05 in bold)

### Cost–utility analysis

#### Incremental QALYs

Over all scenarios, the largest difference in incremental QALYs between 3L value sets and 3L to 5L crosswalks was 0.06 using Dutch valuations (Table 5, scenario 9), while the largest difference between 5L value sets and 5L to 3L crosswalks was 0.08 using US valuations (Table 6, scenario 33).

**Table 5:** Overview of cost–utility outcomes: Differences between 3L value sets and 3L to 5L crosswalks

Country	Scoring method	Scenario	Patient population	Effect size	Δ utilities 3Lvs–3L to 5L cw (95% CI)	Δ QALYs 3Lvs–3L to 5L cw (95% CI)	Δ incremental QALYs 3Lvs–3L to 5L cw	Δ ICER, €/point 3Lvs–3L to 5L cw	Δ Probability of cost-effectiveness			
									*p*_CE_ (0)	*p*_CE_ (20,000)	*p*_CE_ (30,000)	*p*_CE_ (50,000)
NL	3L value set	(1)	Mild depression	Small	0.01 (0.003; 0.02)	−0.03 (−0.03; −0.02)	0.02	−2628	0	0.10	0.07	0.03
	3L to 5L crosswalk											
US	3L value set				0.08 (0.07; 0.08)	0.04 (0.03; 0.04)	0.01	−5190	0	**0.15**	**0.15**	**0.13**
	3L to 5L crosswalk											
JP	3L value set				−0.02 (−0.03; −0.02)	−0.01 (−0.01; 0.001)	0.02	−844,618	0	**0.42**	**0.47**	**0.47**
	3L to 5L crosswalk											
NL	3L value set	(9)	Severe depression	Large	−0.07 (−0.08; −0.06)	−0.04 (−0.05; −0.04)	**0.06**	−169	0	0	0	0
	3L to 5L crosswalk											
US	3L value set				0.09 (0.08; 0.09)	0.06 (0.06; 0.07)	0.004	−16	0	0	0	0
	3L to 5L crosswalk											
JP	3L value set				−0.03 (−0.04; −0.02)	−0.03 (−0.04; −0.02)	0.03	−266	0	0	0	0
	3L to 5L crosswalk											
NL	3L value set	(11)	Mild low back pain	Medium	0.06 (0.05; 0.06)	−0.01 (−0.01; 0.001)	0.02	−761	0	0	0	0
	3L to 5L crosswalk											
US	3L value set				0.06 (0.05; 0.07)	0.03 (0.03; 0.04)	−0.01	532	0	0	0	0
	3L to 5L crosswalk											
JP	3L value set				−0.04 (−0.04; −0.04)	0.004 (0.0004; 0.01)	0.003	−251	0	0	0	0
	3L to 5L crosswalk											
NL	3L value set	(19)	Mild osteoarthritis	Small	0.05 (0.05; 0.06)	0.05 (0.05; 0.05)	0.004	−533	0	−0.01	−0.02	−0.03
	3L to 5L crosswalk											
US	3L value set				0.06 (0.05; 0.07)	0.05 (0.05; 0.06)	0.01	−3176	0	**0.14**	**0.13**	**0.11**
	3L to 5L crosswalk											
JP	3L value set				−0.04 (−0.04; −0.03)	0.02 (0.01; 0.02)	0.02	−38,836	0	**0.37**	**0.15**	**0.15**
	3L to 5L crosswalk											
NL	3L value set	(21)	Mild osteoarthritis	Large	0.05 (0.05; 0.06)	0.05 (0.05; 0.06)	0.002	−211	0	−0.001	0	0
	3L to 5L crosswalk											
US	3L value set				0.06 (0.05; 0.07)	0.05 (0.05; 0.06)	0.01	−2161	0	0.05	0.002	0
	3L to 5L crosswalk											
JP	3L value set				−0.04 (−0.04; −0.03)	0.04 (0.04; 0.05)	0.05	−5954	0	**0.08**	0.03	0.001
	3L to 5L crosswalk											
NL	3L value set	(23)	Moderate osteoarthritis	Medium	0.08 (0.07; 0.08)	0.001 (−0.03; 0.01)	0.01	−499	0	−0.002	−0.001	0
	3L to 5L crosswalk											
US	3L value set				0.11 (0.11; 0.12)	0.05 (0.05; 0.06)	−0.02	1708	0	−0.01	−0.001	0
	3L to 5L crosswalk											
JP	3L value set				−0.02 (−0.02; −0.02)	0.02 (0.02; 0.03)	0.002	−240	0	−0.002	−0.001	0
	3L to 5L crosswalk											
NL	3L value set	(29)	Mild cancer	Medium	0.05 (0.04; 0.05)	0.01 (0.01; 0.02)	0.003	−132	0	0.01	0.003	0
	3L to 5L crosswalk											
US	3L value set				0.02 (0.02; 0.03)	0.01 (0.01; 0.01)	0.01	−455	0	0.03	0.01	0.001
	3L to 5L crosswalk											
JP	3L value set				0.02 (0.01; 0.02)	0.02 (0.02; 0.02)	0.03	−1085	0	**0.06**	0.02	0.003
	3L to 5L crosswalk											

Δ differences; *3Lvs* EQ-5D-3L value set; *cw* crosswalk; *NL* the Netherlands; *US* United States; *JP* Japan; *CI* confidence interval; *ICER* incremental cost-effectiveness ratio; *pCE(0)* probability of cost-effectiveness at a zero willingness-to-pay per QALY gained; *pCE(10,000)* probability of cost-effectiveness at a willingness-to-pay per QALY gained of 10,000 euros

Scenario 9 represents the largest difference in incremental QALYs between 3L value sets and 3L to 5L crosswalks across all scenarios (i.e., 0.06 in bold)

Scenarios 1, 11, 19, 21, and 29 were presented to illustrate the impact of crosswalks on ICERs and probabilities of cost-effectiveness for small, medium, and large treatment effect sizes

**Table 6:** Overview of cost–utility outcomes: differences between 5L value sets and 5L to 3L crosswalks

Country	Scoring method	Scenario	Patient population	Effect size	Δ utilities 5Lvs–5L to 3L cw (95% CI)	Δ QALYs 5Lvs–5L to 3L cw (95% CI)	Δ incremental QALYs 5Lvs–5L to 3L cw	Δ ICER, €/point 5Lvs – 5L to 3L cw	Δ Probability of cost-effectiveness			
									p_CE_ (0)	p_CE_ (20,000)	p_CE_ (30,000)	p_CE_ (50,000)
NL	5L value set	(1)	Mild depression	Small	−0.03 (−0.03; −0.01)	0.001 (−0.003; 0.005)	0.001	−385	0	−0.003	0.001	−0.01
	5L to 3L crosswalk											
US	5L value set				−0.06 (−0.08; −0.05)	−0.05 (−0.05; −0.04)	0.01	−2226	0	0.05	0.02	0.001
	5L to 3L crosswalk											
JP	5L value set				0.01 (0.005; 0.01)	0.01 (0.01; 0.02)	−0.01	7598	0	**−0.25**	**−0.26**	**−0.16**
	5L to 3L crosswalk											
NL	5L value set	(17)	Severe low back pain	Medium	−0.18 (−0.18; −0.16)	−0.07 (−0.08; −0.07)	0.02	−724	0	0	0	0
	5L to 3L crosswalk											
US	5L value set				−0.33 (−0.34; −0.31)	−0.18 (−0.19; −0.18)	0.03	−1741	0	0.003	0	0
	5L to 3L crosswalk											
JP	5L value set				−0.05 (−0.06; −0.04)	−0.15 (−0.15; −0.14)	0.01	−1315	0	**0.01**	0	0
	5L to 3L crosswalk											
NL	5L value set	(19)	Mild osteoarthritis	Small	−0.001 (−0.01; 0.004)	0.01 (0.01; 0.02)	−0.002	181	0	−0.03	−0.03	−0.03
	5L to 3L crosswalk											
US	5L value set				−0.006 (−0.02; 0.01)	−0.01 (−0.02; −0.01)	0.01	−980	0	0.04	0.02	0.01
	5L to 3L crosswalk											
JP	5L value set				0.06 (0.05; 0.06)	0.04 (0.04; 0.05)	0.004	−644	0	0.001	−0.02	−0.02
	5L to 3L crosswalk											
NL	5L value set	(24)	Moderate osteoarthritis	Large	−0.002 (−0.01; 0.002)	0.01 (0.01; 0.01)	**−0.01**	−206	0	0	0	0
	5L to 3L crosswalk											
US	5L value set				−0.03 (−0.04; −0.03)	−0.02 (−0.03; −0.02)	0.02	390	0	0	0	0
	5L to 3L crosswalk											
JP	5L value set				0.06 (0.05; 0.06)	0.04 (0.04; 0.05)	0.01	165	0	0	0	0
	5L to 3L crosswalk											
NL	5L value set	(28)	Mild cancer	Small	−0.01 (−0.01; −0.003)	0.01 (0.001; 0.01)	0.003	−4262	0	0.05	0.05	0.07
	5L to 3L crosswalk											
US	5L value set				−0.03 (−0.04; −0.02)	−0.03 (−0.04; −0.02)	0.01	−14,380	0	**0.18**	**0.18**	**0.15**
	5L to 3L crosswalk											
JP	5L value set				0.01 (0.01; 0.02)	0.02 (0.02; 0.03)	−0.01	3523	0	**−0.12**	**−0.12**	**−0.10**
	5L to 3L crosswalk											
NL	5L value set	(31)	Moderate cancer	Small	−0.03 (−0.03; −0.02)	0.001 (−0.003; 0.01)	−0.02	1746	0	−0.11	−0.08	−0.06
	5L to 3L crosswalk											
US	5L value set				−0.06 (−0.07; −0.05)	−0.04 (−0.05; −0.04)	**0.01**	−1799	0	0.03	−0.0003	−0.01
	5L to 3L crosswalk											
JP	5L value set				0.01 (0.01; 0.02)	0.01 (0.01; 0.02)	0.06	−66,527	0	**0.63**	0.59	**0.54**
	5L to 3L crosswalk											
NL	5L value set	(33)	Moderate cancer	Large	−0.03 (−0.03; −0.02)	−0.03 (−0.03; −0.02)	0.03	96	0	0	0	0
	5L to 3L crosswalk											
US	5L value set				−0.06 (−0.07; −0.05)	−0.07 (−0.08; −0.07)	**0.08**	354	0	0	0	0
	5L to 3L crosswalk											
JP	5L value set				0.01 (0.01; 0.02)	0.001 (−0.005; 0.01)	−0.001	−17	0	0	0	0
	5L to 3L crosswalk											

Δ differences; *5Lvs* EQ-5D-5L value set; *cw* crosswalk; *NL* the Netherlands; *US* United States; *JP* Japan; *CI* confidence interval; *ICER* incremental cost-effectiveness ratio; *pCE(0)* probability of cost-effectiveness at a zero willingness-to-pay per QALY gained; *pCE(10,000)* probability of cost-effectiveness at a willingness-to-pay per QALY gained of 10,000 euros

Scenario 33 represents the largest difference in incremental QALYs between 5L value sets and 5L to 3L crosswalks across all scenarios (i.e., 0.08 in bold)

Scenarios 1, 17, 19, 24 28 and 31 were presented to illustrate the impact of crosswalks on ICERs and probabilities of cost-effectiveness for small, medium, and large treatment effect sizes

#### ICER

The largest differences in ICERs between crosswalks and EQ-5D value sets were found in scenarios with small effect sizes, particularly those with mild health states regardless to the health condition (Table 5, scenarios 1 and 19; Table 6 scenarios 1, 19, 28, 31). Depending on the country, the magnitude of the difference in ICERs was so large that it could in turn impact the decision of whether an intervention is cost-effective or not (i.e., whether the ICER lies below a country’s WTP per QALY gained). For example, in the scenario 1, ICERs estimated by 3L to 5L crosswalk, and the Japanese 3L value set differed tremendously, with the biggest difference being €11,063/QALY gained for the 3L to 5L crosswalk and €855,681/QALY gained for the Japanese 3L value set (Appendix 3). The differences in ICERs were generally larger for the EQ-5D-3L compared with the EQ-5D-5L and were most pronounced for Japan. Detailed information on ICERs can be found in Appendix 4.

#### Probabilities of cost-effectiveness

Larger differences between crosswalks and EQ-5D value sets were found in scenarios with small treatment effect sizes, while this was less evident for scenarios with medium and large ones. For example, for small effect sizes, at a WTP of €20,000/QALY gained, the largest differences in the probability of cost-effectiveness between EQ-5D value sets and crosswalks were found for mild depression (difference between 3L value set and 3L to 5L crosswalk = 0.42, Table 5, scenario 1) and moderate cancer (difference between 5L value set and 5L to 3L crosswalk = 0.63, Table 6, scenario 31) using Japanese valuations. For medium effect sizes, at the same WTP threshold, the largest differences were found for mild cancer (difference between 3L value set and 3L to 5L crosswalk = 0.06, Table 5, scenario 29) and for severe low back pain (difference between 5L value set and 5L to 3L crosswalk = 0.01, Table 6, scenario 17) using Japanese valuations. For large effect sizes, the largest difference was found for mild osteoarthritis using Japanese valuations (difference between 3L value set and 3L to 5L crosswalk = 0.08, Table 5, scenario 21) and no differences were found between 5L value sets and 5L to 3L crosswalks. At a WTP of €50,000/QALY gained, the largest differences were found in scenarios including small effect sizes for mild depression (difference between 3L value set and 3L to 5L crosswalk = 0.47, Table 5, scenario 1) and moderate cancer (difference between 5L value set and 5L to 3L crosswalk = 0.54, Table 6, scenario 31) using Japanese valuations, while no differences were found in all scenarios with medium and large effect sizes, except for severe osteoarthritis using Dutch valuations (difference between 3L value set and 3L to 5L crosswalk = 0.01, Table 5, scenario 26).

## Discussion

### Main findings

The aim of the current study was to evaluate the impact of using crosswalks or EQ-5D value sets on reimbursement decisions in a wide variety of simulated trial-based economic evaluations for the Netherlands, the United States, and Japan. Results showed that differences exist in means and distributions of utilities, incremental QALYs, and ICER point estimates between scoring methods in all simulated scenarios and countries. In our study, this only affected reimbursement decisions in scenarios with small treatment effect sizes, especially in mild health states regardless of the health condition. This impact was more pronounced in the United States and Japan than in the Netherlands. In scenarios with medium and large effect sizes, the impact on the probability of cost-effectiveness was relatively small in all countries. Our findings suggest that caution is warranted when using crosswalks, especially when treatment effect sizes are small and in countries that were not included in the crosswalk development studies (i.e., all countries except Denmark, England, Italy, the Netherlands, Poland, and Scotland).

### Interpretation of the findings and comparison with the literature

In line with previous studies [18, 24–29], our study found that different EQ-5D scoring methods resulted in different utilities estimates, which in turn resulted in different incremental QALY and ICER estimates. Differences in utilities and QALYs between EQ-5D scoring methods in certain scenarios and conditions may be due to differences in utility decrements between health dimensions in the different value sets but also to the probability of observing certain response levels within conditions (e.g., low back pain patients have a high probability of scoring severe response levels on the “pain/discomfort” dimension). The magnitude of the differences and their clinical relevance differed across countries, with differences generally being larger in the United States and Japan than in the Netherlands.

A previous study concluded that there was no impact on reimbursement decisions of the scoring method used [29]. In contrast, we now show that in some scenarios, particularly those with small treatment effect sizes, the use of crosswalks instead of country-specific EQ-5D value sets impacts cost–utility outcomes to such an extent that this may influence reimbursement decisions. The difference in findings and conclusion between our previous and current study may be explained by the fact that the interventions of the case studies used in our previous study were on average “less effective” and “more costly” than control. In the present study, we simulated scenarios with interventions that were “more effective” and “more costly”, which is a more likely scenario to occur in real-life reimbursement decisions. Our current findings also show that different EQ-5D scoring approaches were more likely to impact a reimbursement decision for countries that were not used in the development of the crosswalk. This may be due to the fact that the sample included in the crosswalk development study may not represent the preferences of other populations, particularly those with considerably different views on health-related quality of life.

### Strengths and limitations

One of the strengths of this study is that the impact on cost–utility outcomes was evaluated for three different countries, two of which were not used for the development of the crosswalk and differed considerably from the Dutch value set in terms of the utility decrements assigned to the different health dimensions of the EQ-5D [20, 22]. Another strength is our use of simulated data and a wide range of scenarios. These scenarios were based on empirical studies in chronic health conditions that have a high impact on populations’ health-related quality of life and/or life expectancy. Moreover, the simulated scenarios included different severity levels of the included health conditions and interventions with small, medium, and large impacts on health-related quality of life. Furthermore, full trial-based economic evaluations were performed including the assessment of uncertainty around ICER estimates.

A limitation of this study is that cost data were simulated in such a way that cost differences were not statistically significant, but we do not expect this to change our overall conclusion that caution is warranted when using crosswalks for estimating EQ-5D utilities, particularly when effect sizes are small. Additionally, only three countries were investigated, whereas EQ-5D value sets are available for many countries. However, we deliberately chose countries with considerably different utility decrements to include the full spectrum of preferences from other countries.

### Recommendations for research and practice

The current results indicate that the use of crosswalks may impact on reimbursement decisions in situations where treatment effect sizes are small, and interventions are more costly compared to control. Given the rigorous quality control protocols for the EQ-5D valuation studies, the most appropriate EQ-5D scoring method is the available country-specific value set developed using the most recent version of the EQ-VT protocol [15]. In case of multi-country randomized clinical-trials, researchers are recommended to check the HTA guidelines of the participating countries for the most appropriate choice. Nonetheless, there are cases in which the decision on which value set to use is more complex, such as when a value set is only available for one of the EQ-5D version, while data have been collected using the other version of the EQ-5D. In such situations, caution is needed when using crosswalks as they may impact cost–utility outcomes, particularly in countries that were not included in the developments of the crosswalks. For further details and guidance about the choice of scoring methods, researchers are advised to check EuroQol recommendations [23].

It is important to note that health economic models submitted to HTA agencies rarely use directly measured utilities, and that there is considerable freedom in which utilities are used. Thus, the finding of this study that there are considerable differences between the different valuation approaches do not necessarily result in an impact on QALY estimates in these models.

## Conclusion

Crosswalks may be used when value sets are missing for a specific country or jurisdiction. However, our findings indicate that reimbursement decisions may change in situations with small effect sizes and countries that were not included in the development of the crosswalks. Therefore, when EQ-5D value sets are not available, researchers and decision-makers should be aware that the use of crosswalk is likely to impact decisions.

### Supplementary Information

Below is the link to the electronic supplementary material.

Supplementary file1 (DOCX 3625 KB)
