# Supplementary material for: To what extent does the use of crosswalks instead of EQ-5D value sets impact reimbursement decisions?: a simulation study
Source: Eur J Health Econ. 2022 Nov 13;24(8):1253–70. doi: 10.1007/s10198-022-01539-6 (PMC10533624; doi:10.1007/s10198-022-01539-6)
Supplement: Supplementary file 1 — Supplementary file1 (DOCX 3625 KB) [file 10198_2022_1539_MOESM1_ESM.docx]

**APPENDIX 1. Cut-off points of severity levels**

| **Health condition** | **Measure** | **Mild** | **Moderate** | **Severe** |
| --- | --- | --- | --- | --- |
| Depression | MADRS[30, 37] | 0–26 | 27–34 | 35–60 |
|  | IDS-SR[30, 38] | 0–38 | 39–48 | 49–84 |
|  | HADS-D[30, 39] | 0–19 | 20–25 | 26–52 |
|  | BDI-II[40] | 0–19 | 20–28 | 29–63 |
| Low back pain and osteoarthritis | NRS[41] | 0–3 | 4–6 | 7–10 |
| Cancer | MSI[42, 43] | 0–10 | 11–16 | 17–20 |
|  | ECOG[44] | 0-1 | 2 | 3-4 |

MADRS: Montgomery–Åsberg Depression Rating Scale. IDS-SR: The Inventory of Depressive Symptomatology, Clinician Rating and Self-Report. HADS: Hospital Anxiety and Depression Scale. BDI-II: Beck Depression Inventory-II. NRS: Numeric Rating Scale. MSI: Multidimensional Fatigue Inventory. ECOG: Eastern Cooperative Oncology Group performance status.

**APPENDIX 2. R script – Data generation**

# EQ-5D-3L Baseline Profile Generator for R

# Version 2.00 March 2019

# 1. load libraries

library(readr)

# 2. Set up a working diretory

setwd("<<directory path>>")

# 3. Load the function profGen.f to generate baseline profiles for the 3L version

profGen.f <<-function(cases){

dim.name <- c("Mobility","Self Care","Usual Act","Pain & Dis","Anx & Dep","Profile")

baseProf.m <<- matrix(0,nrow=cases, ncol=6)

colnames(baseProf.m) <<- dim.name

for (v1 in 1:cases) {

prof=0

for (v2 in 1:5) {

P=runif(1)

if(P <= prob.m[v2,1]) {

baseProf.m[v1,v2] <<- 1

}else{

if(P <=(prob.m[v2,1]+prob.m[v2,2])) {

baseProf.m[v1,v2] <<- 2

}else { baseProf.m[v1,v2] <<- 3 }

}

prof=prof+(baseProf.m[v1,v2]*(10^(5-v2)))

}

baseProf.m[v1,6] <<- prof

}

cat("\n","First 10 profiles","\n")

print(baseProf.m[1:10,1:6])

}

#*******************************************************************************

## DISEASE (d) [i.e., DEPRESSION, LOW BACK PAIN, OSTEOARTHRITIS, CANCER]

## BASELINE PROFILE – MILD

## Import the disease baseline probability matrix control group

prob.m <- read.csv("d_bpm_ml_t0.csv", row.names=1)

profGen.f(150) # generate baseline profiles

write.csv(baseProf.m, "d_bp_ml_t0.csv") # save baseline profiles

## Import the disease baseline probability matrix treatment group

prob.m <- read.csv("d_bpm_ml_t1.csv", row.names=1)

profGen.f(150)

write.csv(baseProf.m, "d_bp_ml_t1.csv")

## BASELINE PROFILE – MODERATE

## Import the disease baseline probability matrix control group

prob.m <- read.csv("d_bpm_mo_t0.csv", row.names=1)

profGen.f(150)

write.csv(baseProf.m, "d_bp_mo_t0.csv")

## Import the disease baseline probability matrix treatment group

prob.m <- read.csv("d_bpm_mo_t1.csv", row.names=1)

profGen.f(150)

write.csv(baseProf.m, "d_bp_mo_t1.csv")

## BASELINE PROFILE – SEVERE

## Import the disease baseline probability matrix control group

prob.m <- read.csv("d_bpm_se_t0.csv", row.names=1)

profGen.f(150)

write.csv(baseProf.m, "d_bp_se_t0.csv")

## Import the disease baseline probability matrix treatment group

prob.m <- read.csv("d_bpm_se_t1.csv", row.names=1)

profGen.f(150)

write.csv(baseProf.m, "d_bp_se_t1.csv")

################################################################################

# Package: EQSimLab3L

# Title: EQ-5D-3L Simulation Laboratory

# Version: 0.0.0.9000

# 1. Load libraries

library(readr)

library(eq5d)

library(foreign)

# 2. Set up a working diretory

setwd("<<directory path>>")

# 3. generate follow-up profiles by treatment group

## TREATMENT GOUP

eq_make_profile_change()

# Do you want to import probabilities from a data file? (y = yes) > y

# File Name? > 0-tp-mld3l-t1.txt

# # Base profile data file name ? > d_bp_mlt1.txt

# Matched outcome profile data file name ? > d_fp_mlt1.txt

## CONTROL GROUP - the same transition probabilities as mild depression small effect size

eq_make_profile_change()

# Do you want to import probabilities from a data file? (y = yes) > y

# File Name? > 0-tp-mld3l-t0.txt

# Base profile data file name ? > d_bp_mlt0.txt

# Matched outcome profile data file name ? > d_fp_mlt0.txt

# 4. Calculate baseline and follow-up utilities

# EQ-5D-5L country-specific value sets must be one of: Canada, China, Denmark, Egypt, England,

# Ethiopia, France, Germany, HongKong, Hungary, Indonesia, Ireland, Japan, Malaysia, Netherlands,

# Peru_cTTO, Peru_DCE, Poland, Portugal, SouthKorea, Spain, Sweden, Taiwan, Thailand, Uruguay,

# USA, Vietnam

# 4.1 baseline utilities control group

`baseProf.m.t0` <- read.csv("~/2020-EuroQol-call/EQ5D_laboratory/probabilities/d_bp_mlt0.txt", sep="")

names(baseProf.m.t0)[1] <- "id"

names(baseProf.m.t0)[2] <- "MO"

names(baseProf.m.t0)[3] <- "SC"

names(baseProf.m.t0)[4] <- "UA"

names(baseProf.m.t0)[5] <- "PD"

names(baseProf.m.t0)[6] <- "AD"

names(baseProf.m.t0)[7] <- "Profile.b"

baseProf.m.t0$trt <- 0

baseProf.m.t0$NL.utility.b <- eq5d(baseProf.m.t0, type="TTO", version="3L", country = "Netherlands", ignore.incomplete = TRUE)

baseProf.m.t0$US.utility.b <- eq5d(baseProf.m.t0, type="TTO", version="3L", country = "USA", ignore.incomplete = TRUE)

baseProf.m.t0$JP.utility.b <- eq5d(baseProf.m.t0, type="TTO", version="3L", country = "Japan", ignore.incomplete = TRUE)

# 4.2 follow-up utilities control group

`outProf.m.t0` <- read.csv("~/2020-EuroQol-call/EQ5D_laboratory/probabilities/d_fp_mlt0.txt", sep="")

names(outProf.m.t0)[1] <- "id"

names(outProf.m.t0)[2] <- "MO"

names(outProf.m.t0)[3] <- "SC"

names(outProf.m.t0)[4] <- "UA"

names(outProf.m.t0)[5] <- "PD"

names(outProf.m.t0)[6] <- "AD"

names(outProf.m.t0)[7] <- "Profile.f"

outProf.m.t0$trt <- 0

outProf.m.t0 <- data.frame(lapply(outProf.m.t0, function(x) as.numeric(as.character(x))))

outProf.m.t0$NL.utility.f <- eq5d(outProf.m.t0, type="TTO", version="3L", country = "Netherlands", ignore.incomplete = TRUE)

outProf.m.t0$US.utility.f <- eq5d(outProf.m.t0, type="TTO", version="3L", country = "USA", ignore.incomplete = TRUE)

outProf.m.t0$JP.utility.f <- eq5d(outProf.m.t0, type="TTO", version="3L", country = "Japan", ignore.incomplete = TRUE)

# 4.3. Merge baseline and follow-up data control group

control <- merge(baseProf.m.t0, outProf.m.t0, by = "id")

# 4.4 baseline utilities treatment group

`baseProf.m.t1` <- read.csv("~/2020-EuroQol-call/EQ5D_laboratory/probabilities/dep_bp_mlt1.txt", sep="")

names(baseProf.m.t1)[1] <- "id"

names(baseProf.m.t1)[2] <- "MO"

names(baseProf.m.t1)[3] <- "SC"

names(baseProf.m.t1)[4] <- "UA"

names(baseProf.m.t1)[5] <- "PD"

names(baseProf.m.t1)[6] <- "AD"

names(baseProf.m.t1)[7] <- "Profile.b"

baseProf.m.t1$trt <- 1

baseProf.m.t1$NL.utility.b <- eq5d(baseProf.m.t1, type="TTO", version="3L", country = "Netherlands", ignore.incomplete = TRUE)

baseProf.m.t1$US.utility.b <- eq5d(baseProf.m.t1, type="TTO", version="3L", country = "USA", ignore.incomplete = TRUE)

baseProf.m.t1$JP.utility.b <- eq5d(baseProf.m.t1, type="TTO", version="3L", country = "Japan", ignore.incomplete = TRUE)

# 4.5 follow-up utilities treatment group

`outProf.m.t1` <- read.csv("~/2020-EuroQol-call/EQ5D_laboratory/probabilities/dep_fp_mlt1.txt", sep="")

names(outProf.m.t1)[1] <- "id"

names(outProf.m.t1)[2] <- "MO"

names(outProf.m.t1)[3] <- "SC"

names(outProf.m.t1)[4] <- "UA"

names(outProf.m.t1)[5] <- "PD"

names(outProf.m.t1)[6] <- "AD"

names(outProf.m.t1)[7] <- "Profile.f"

outProf.m.t1$trt <- 0

outProf.m.t1 <- data.frame(lapply(outProf.m.t1, function(x) as.numeric(as.character(x))))

outProf.m.t1$NL.utility.f <- eq5d(outProf.m.t1, type="TTO", version="3L", country = "Netherlands", ignore.incomplete = TRUE)

outProf.m.t1$US.utility.f <- eq5d(outProf.m.t1, type="TTO", version="3L", country = "USA", ignore.incomplete = TRUE)

outProf.m.t1$JP.utility.f <- eq5d(outProf.m.t1, type="TTO", version="3L", country = "Japan", ignore.incomplete = TRUE)

# 4.6. Merge baseline and follow-up data treatment group

treatment <- merge(baseProf.m.t1, outProf.m.t1, by = "id")

# 5. Merge treatment and control

small.eff.size <- rbind(treatment, control)

# 6. Calculate cohen's d = mean difference between groups/ sd

small.eff.size$NL.QALY <- (0.5*(small.eff.size$NL.utility.b + small.eff.size$NL.utility.f))

small.eff.size$US.QALY <- (0.5*(small.eff.size$US.utility.b + small.eff.size$US.utility.f))

small.eff.size$JP.QALY <- (0.5*(small.eff.size$US.utility.b + small.eff.size$JP.utility.f))

# 7. Prepare data to save in .dta

colnames(small.eff.size)[colnames(small.eff.size)=="trt.x"] <- "trt"

colnames(small.eff.size)[colnames(small.eff.size)=="MO.x"] <- "MO_BASELINE"

colnames(small.eff.size)[colnames(small.eff.size)=="SC.x"] <- "SC_BASELINE"

colnames(small.eff.size)[colnames(small.eff.size)=="UA.x"] <- "UA_BASELINE"

colnames(small.eff.size)[colnames(small.eff.size)=="PD.x"] <- "PD_BASELINE"

colnames(small.eff.size)[colnames(small.eff.size)=="AD.x"] <- "AD_BASELINE"

colnames(small.eff.size)[colnames(small.eff.size)=="MO.y"] <- "MO_T1"

colnames(small.eff.size)[colnames(small.eff.size)=="SC.y"] <- "SC_T1"

colnames(small.eff.size)[colnames(small.eff.size)=="UA.y"] <- "UA_T1"

colnames(small.eff.size)[colnames(small.eff.size)=="PD.y"] <- "PD_T1"

colnames(small.eff.size)[colnames(small.eff.size)=="AD.y"] <- "AD_T1"

small.eff.size$trt.y <- NULL

# 8. Check effect size NL, US, JP

NL.lm <-lm(NL.QALY ~ trt, data = small.eff.size)

summary(NL.lm)

sd.NL <- sd(small.eff.size$NL.QALY)

cohen.d.NL <- NL.lm[["coefficients"]][["trt"]]/sd.NL

cohen.d.NL

US.lm <-lm(US.QALY ~ trt, data = small.eff.size)

summary(US.lm)

sd.US <- sd(small.eff.size$US.QALY)

cohen.d.US <-US.lm[["coefficients"]][["trt"]]/sd.US

cohen.d.US

JP.lm <-lm(JP.QALY ~ trt, data = small.eff.size)

summary(JP.lm)

sd.JP <- sd(small.eff.size$JP.QALY)

cohen.d.JP <- JP.lm[["coefficients"]][["trt"]]/sd.JP

cohen.d.JP

small.eff.size <<- cbind(Case =c(1:nrow(small.eff.size)),small.eff.size)

small.eff.size$id <- NULL

colnames(small.eff.size)[colnames(small.eff.size)=="Case"] <- "id"

write.dta(small.eff.size, file = "<<directory path>>/ml-small-effsize.dta")

################################################################################

# Generate age, gender, and costs

# 1. Load libraries

library(haven)

library(simstudy)

library(foreign)

# 2. Import EQ-5D dataset and prepare to merge with simulated dataset

setwd("<<directory path>>")

dataset <- read_dta("ml-small-effsize.dta") # replace the name of EQ-5D dataset here

# 3. Generate baseline characteristics

def <- defData(varname = "age", dist="uniformInt", formula="25;75", id="id")

def <- defData(def, varname = "gender", formula = 0.19, dist = "binary", id="id")

simulatie <- genData(300, def)

# 4. Merge baseline characteristics and EQ-5D dataset

simulatie <- merge(simulatie,dataset, by ="id")

# 5. Generate correlated costs and QALYs

def1 <- defDataAdd(varname = "costs", formula = "2000 + 250*trt", variance = 1, dist = "gamma")

simulatie <- addColumns(def1, simulatie)

simulatie <- addCorFlex(simulatie, def1, rho = 0.75, corstr = "cs")

# 6. Check correlation

correlation <- simulatie[,cor(NL_QALY, i.costs)]

correlation

# 7. Save data in .dta

write.dta(simulatie, file = "<<directory path>>/ml-small-effsize.dta")

################################################################################

# Package: EQSimLab5L

# Title: EQ-5D-5L Simulation Laboratory

# Version: 0.0.0.9000

# 1. Load libraries

library(EQSimLab5L)

library(readr)

library(dplyr)

library(eq5d)

library(foreign)

# 2. Set up a working diretory

setwd("<<directory path>>")

# 3. Generate baseline profiles per treatment group

## TREATMENT GOUP

eq_make_profile_data(150)

#Do you want to import probabilities? (y = yes) > n

# These are the probabilities that you have specified:

# Level 1 Level 2 Level 3 Level 4 Level 5

# Mobility 0.00 0.25 0.50 0.25 0

# Self Care 0.50 0.25 0.25 0.00 0

# Usual Activities 0.00 0.25 0.25 0.50 0

# Pain & Discomfort 0.00 0.00 0.50 0.50 0

# Anxiety & Depression 0.25 0.50 0.00 0.25 0

# Save probability matrix? (y = yes) > y

# Save File Name? > d_bpm_ml5l_t1.txt

# Do you want to randomise by Profile or Dimension? (Choose p for Profile) > p

# Save File Name? > d_bp_ml5l_t1.txt

## CONTROL GROUP

eq_make_profile_data(150)

#Do you want to import probabilities? (y = yes) > n

# These are the probabilities that you have specified:

# Level 1 Level 2 Level 3 Level 4 Level 5

# Mobility 0.00 0.00 0.75 0.25 0

# Self Care 0.25 0.50 0.25 0.00 0

# Usual Activities 0.25 0.00 0.25 0.50 0

# Pain & Discomfort 0.00 0.00 0.75 0.25 0

# Anxiety & Depression 0.25 0.25 0.50 0.00 0

# Save probability matrix? (y = yes) > y

# Save File Name? > d_bpm_ml5l_t0.txt

# Do you want to randomise by Profile or Dimension? (Choose p for Profile) > p

# Save File Name? > d_bp_ml5l_t0.txt

# 4. Generate follow-up profiles per treatment group

## TREATMENT GOUP

eq_make_profile_change()

# Do you want to import probabilities from a data file? (y = yes) > y

# File Name? > d_tp_ml5l_t1.txt

# Base profile data file name ? > d_bp_ml5l_t1.txt

# Matched outcome profile data file name ? > d_fp_ml5l_t1.txt

## CONTROL GROUP

eq_make_profile_change()

# Do you want to import probabilities from a data file? (y = yes) > y

# File Name? > d_tp_ml5l_t0.txt

# File name ? > d_bp_ml5l_t0.txt

# Matched outcome profile data file name ? > d_fp_ml5l_t0.txt

# 5. Calculate baseline and follow-up utilities

# 5.1 baseline utilities control group

`baseProf.m.t0` <- read.csv("<<directory path>>/d_fp_ml5l_t0.txt", sep="")

names(baseProf.m.t0)[1] <- "id"

names(baseProf.m.t0)[2] <- "MO"

names(baseProf.m.t0)[3] <- "SC"

names(baseProf.m.t0)[4] <- "UA"

names(baseProf.m.t0)[5] <- "PD"

names(baseProf.m.t0)[6] <- "AD"

names(baseProf.m.t0)[7] <- "Profile.b"

baseProf.m.t0$trt <- 0

baseProf.m.t0$NL.utility.b <- eq5d(baseProf.m.t0, type="VT", version="5L", country = "Netherlands", ignore.incomplete = TRUE)

baseProf.m.t0$US.utility.b <- eq5d(baseProf.m.t0, type="VT", version="5L", country = "USA", ignore.incomplete = TRUE)

baseProf.m.t0$JP.utility.b <- eq5d(baseProf.m.t0, type="VT", version="5L", country = "Japan", ignore.incomplete = TRUE)

# 5.2 follow-up utilities control group

`outProf.m.t0` <- read.csv("<<directory path>>/d_fp_ml5l_t0.txt", sep="")

names(outProf.m.t0)[1] <- "id"

names(outProf.m.t0)[2] <- "MO"

names(outProf.m.t0)[3] <- "SC"

names(outProf.m.t0)[4] <- "UA"

names(outProf.m.t0)[5] <- "PD"

names(outProf.m.t0)[6] <- "AD"

names(outProf.m.t0)[7] <- "Profile.f"

outProf.m.t0$trt <- 0

outProf.m.t0 <- data.frame(lapply(outProf.m.t0, function(x) as.numeric(as.character(x))))

outProf.m.t0$NL.utility.f <- eq5d(outProf.m.t0, type="VT", version="5L", country = "Netherlands", ignore.incomplete = TRUE)

outProf.m.t0$US.utility.f <- eq5d(outProf.m.t0, type="VT", version="5L", country = "USA", ignore.incomplete = TRUE)

outProf.m.t0$JP.utility.f <- eq5d(outProf.m.t0, type="VT", version="5L", country = "Japan", ignore.incomplete = TRUE)

# 5.3. Merge baseline and follow-up data control group

control <- merge(baseProf.m.t0, outProf.m.t0, by = "id")

# 5.4 baseline utilities treatment group

`baseProf.m.t1` <- read.csv("<<directory path>>/d_fp_ml5l_t1.txt", sep="")

names(baseProf.m.t1)[1] <- "id"

names(baseProf.m.t1)[2] <- "MO"

names(baseProf.m.t1)[3] <- "SC"

names(baseProf.m.t1)[4] <- "UA"

names(baseProf.m.t1)[5] <- "PD"

names(baseProf.m.t1)[6] <- "AD"

names(baseProf.m.t1)[7] <- "Profile.b"

baseProf.m.t1$trt <- 1

baseProf.m.t1$NL.utility.b <- eq5d(baseProf.m.t1, type="VT", version="5L", country = "Netherlands", ignore.incomplete = TRUE)

baseProf.m.t1$US.utility.b <- eq5d(baseProf.m.t1, type="VT", version="5L", country = "USA", ignore.incomplete = TRUE)

baseProf.m.t1$JP.utility.b <- eq5d(baseProf.m.t1, type="VT", version="5L", country = "Japan", ignore.incomplete = TRUE)

# 5.5 follow-up utilities treatment group

`outProf.m.t1` <- read.csv("<<directory path>>/d_fp_ml5l_t1.txt", sep="")

names(outProf.m.t1)[1] <- "id"

names(outProf.m.t1)[2] <- "MO"

names(outProf.m.t1)[3] <- "SC"

names(outProf.m.t1)[4] <- "UA"

names(outProf.m.t1)[5] <- "PD"

names(outProf.m.t1)[6] <- "AD"

names(outProf.m.t1)[7] <- "Profile.f"

outProf.m.t1$trt <- 0

outProf.m.t1 <- data.frame(lapply(outProf.m.t1, function(x) as.numeric(as.character(x))))

outProf.m.t1$NL.utility.f <- eq5d(outProf.m.t1, type="VT", version="5L", country = "Netherlands", ignore.incomplete = TRUE)

outProf.m.t1$US.utility.f <- eq5d(outProf.m.t1, type="VT", version="5L", country = "USA", ignore.incomplete = TRUE)

outProf.m.t1$JP.utility.f <- eq5d(outProf.m.t1, type="VT", version="5L", country = "Japan", ignore.incomplete = TRUE)

# 5.6. Merge baseline and follow-up data treatment group

treatment <- merge(baseProf.m.t1, outProf.m.t1, by = "id")

# 6. Merge treatment and control

small.eff.size <- rbind(treatment, control)

# 7. Calculate QALY

small.eff.size$NL.QALY <- (0.5*(small.eff.size$NL.utility.b + small.eff.size$NL.utility.f))

small.eff.size$JP.QALY <- (0.5*(small.eff.size$US.utility.b + small.eff.size$JP.utility.f))

small.eff.size$US.QALY <- (0.5*(small.eff.size$US.utility.b + small.eff.size$US.utility.f))

# 8. Check effect size (cohen's d = mean difference bewteen groups/ sd)

colnames(small.eff.size)[colnames(small.eff.size)=="trt.x"] <- "trt"

NL.lm <-lm(NL.QALY ~ trt, data = small.eff.size)

summary(NL.lm)

sd.NL <- sd(small.eff.size$NL.QALY)

cohen.d.NL <- NL.lm[["coefficients"]][["trt"]]/sd.NL

cohen.d.NL

US.lm <-lm(US.QALY ~ trt, data = small.eff.size)

summary(US.lm)

sd.US <- sd(small.eff.size$US.QALY)

cohen.d.US <-US.lm[["coefficients"]][["trt"]]/sd.US

cohen.d.US

JP.lm <-lm(JP.QALY ~ trt, data = small.eff.size)

summary(JP.lm)

sd.JP <- sd(small.eff.size$JP.QALY)

cohen.d.JP <- JP.lm[["coefficients"]][["trt"]]/sd.JP

cohen.d.JP

# 9. Prepare data to save in .dta

colnames(small.eff.size)[colnames(small.eff.size)=="MO.x"] <- "MO_BASELINE"

colnames(small.eff.size)[colnames(small.eff.size)=="SC.x"] <- "SC_BASELINE"

colnames(small.eff.size)[colnames(small.eff.size)=="UA.x"] <- "UA_BASELINE"

colnames(small.eff.size)[colnames(small.eff.size)=="PD.x"] <- "PD_BASELINE"

colnames(small.eff.size)[colnames(small.eff.size)=="AD.x"] <- "AD_BASELINE"

colnames(small.eff.size)[colnames(small.eff.size)=="MO.y"] <- "MO_T1"

colnames(small.eff.size)[colnames(small.eff.size)=="SC.y"] <- "SC_T1"

colnames(small.eff.size)[colnames(small.eff.size)=="UA.y"] <- "UA_T1"

colnames(small.eff.size)[colnames(small.eff.size)=="PD.y"] <- "PD_T1"

colnames(small.eff.size)[colnames(small.eff.size)=="AD.y"] <- "AD_T1"

small.eff.size$trt.y <- NULL

write.dta(small.eff.size, file = "<<directory path>>/ml-small-effsize.dta")

################################################################################

# Generate age, gender, and costs

# 1. Load libraries

library(haven)

library(simstudy)

library(foreign)

# 2. Import EQ-5D dataset and prepare to merge with simulated dataset

dataset <- read_dta("<<directory path>>/ml-small-effsize.dta") # replace the name of EQ-5D dataset here

dataset <- as.data.frame(dataset)

dataset <- remove_label(dataset)

# 3. Generate baseline characteristics

def <- defData(varname = "age", dist="uniformInt", formula="25;75", id="id")

def <- defData(def, varname = "gender", formula = 0.19, dist = "binary", id="id")

simulatie <- genData(300, def)

# 4. Merge baseline characteristics and EQ-5D dataset

simulatie <- cbind(simulatie, dataset)

names(simulatie)[4] <- "id.x"

simulatie$id.x <- NULL

# 5. Generate correlated costs and QALYs

def1 <- defDataAdd(varname = "costs", formula = "2000 + 250*trt", variance = 1, dist = "gamma")

simulatie <- addColumns(def1, simulatie)

# 6. Check correlation

correlation <- simulatie[,cor(NL_QALY, costs)]

correlation

# 7. Save data in .dta

write.dta(simulatie, file = "<<directory path>>/ml-small-effsize.dta")

**APPENDIX 3. Kernel density histograms**

Kernel density histograms comparing utility distributions of the 3L value sets and 3L to 5L crosswalks.

**MILD DEPRESSION**

**
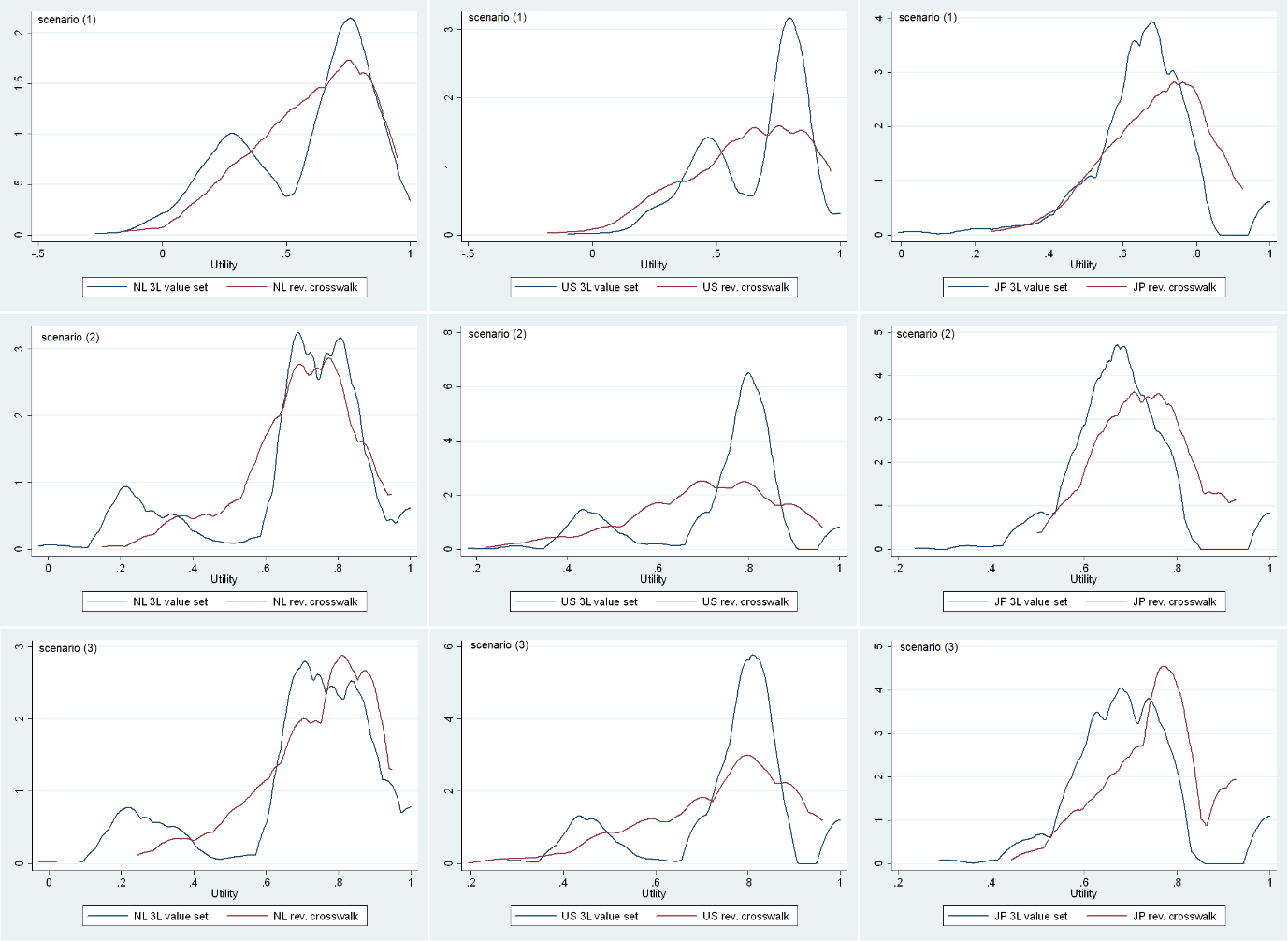
**

Scenario (1): mild depression and small treatment effect size.

Scenario (2): mild depression and medium treatment effect size.

Scenario (3): mild depression and large treatment effect size.

3L value set: EQ-5D-3L value set. rev. crosswalk: 3L to 5L crosswalk

NL: the Netherlands. US: the United States. JP: Japan.

**MODERATE DEPRESSION**

**
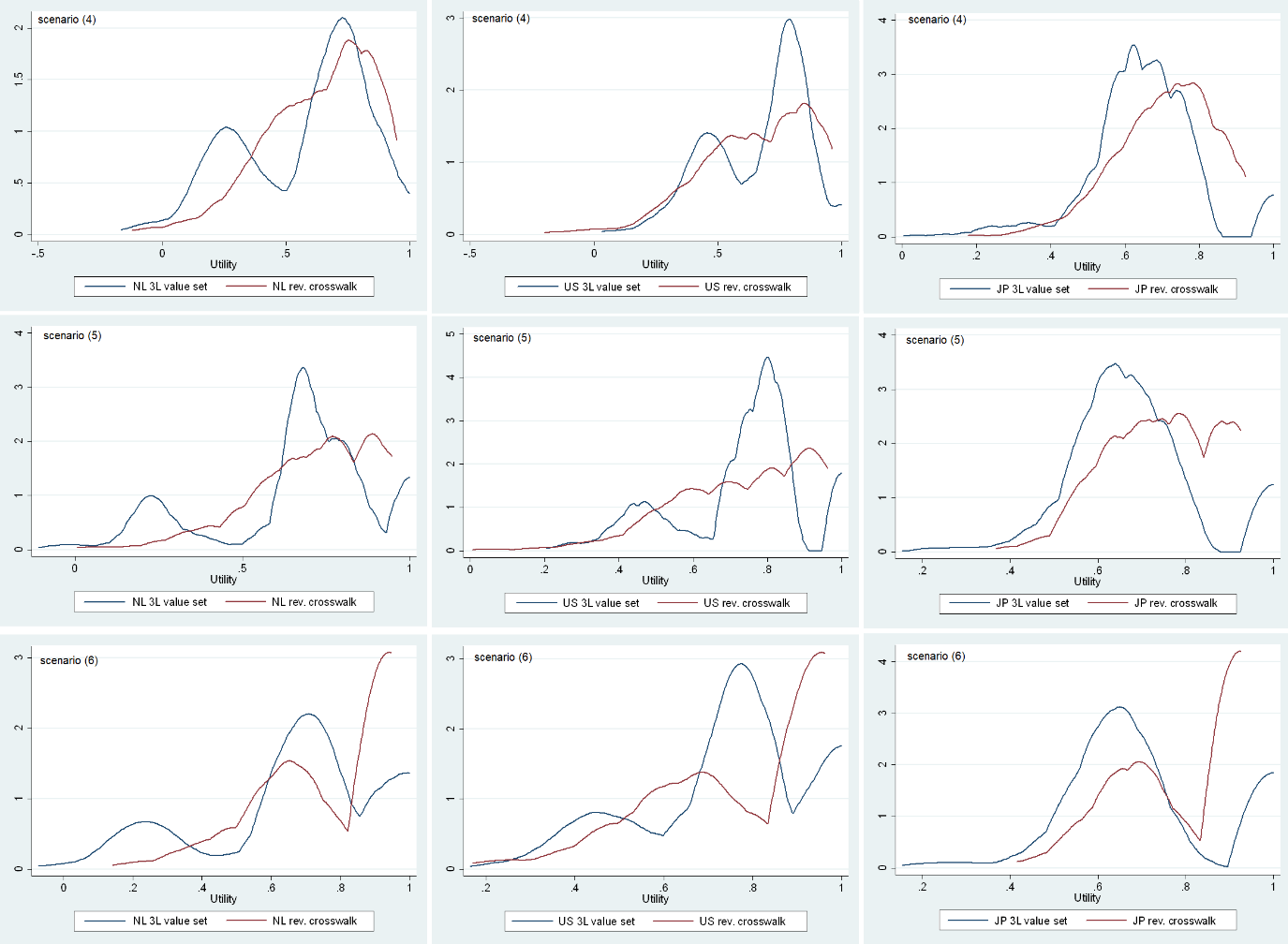
**

Scenario (4): moderate depression and small treatment effect size.

Scenario (5): moderate depression and medium treatment effect size.

Scenario (6): moderate depression and large treatment effect size.

3L value set: EQ-5D-3L value set. rev. crosswalk: 5L to 3L crosswalk

NL: the Netherlands. US: the United States. JP: Japan.

**SEVERE DEPRESSION**

**
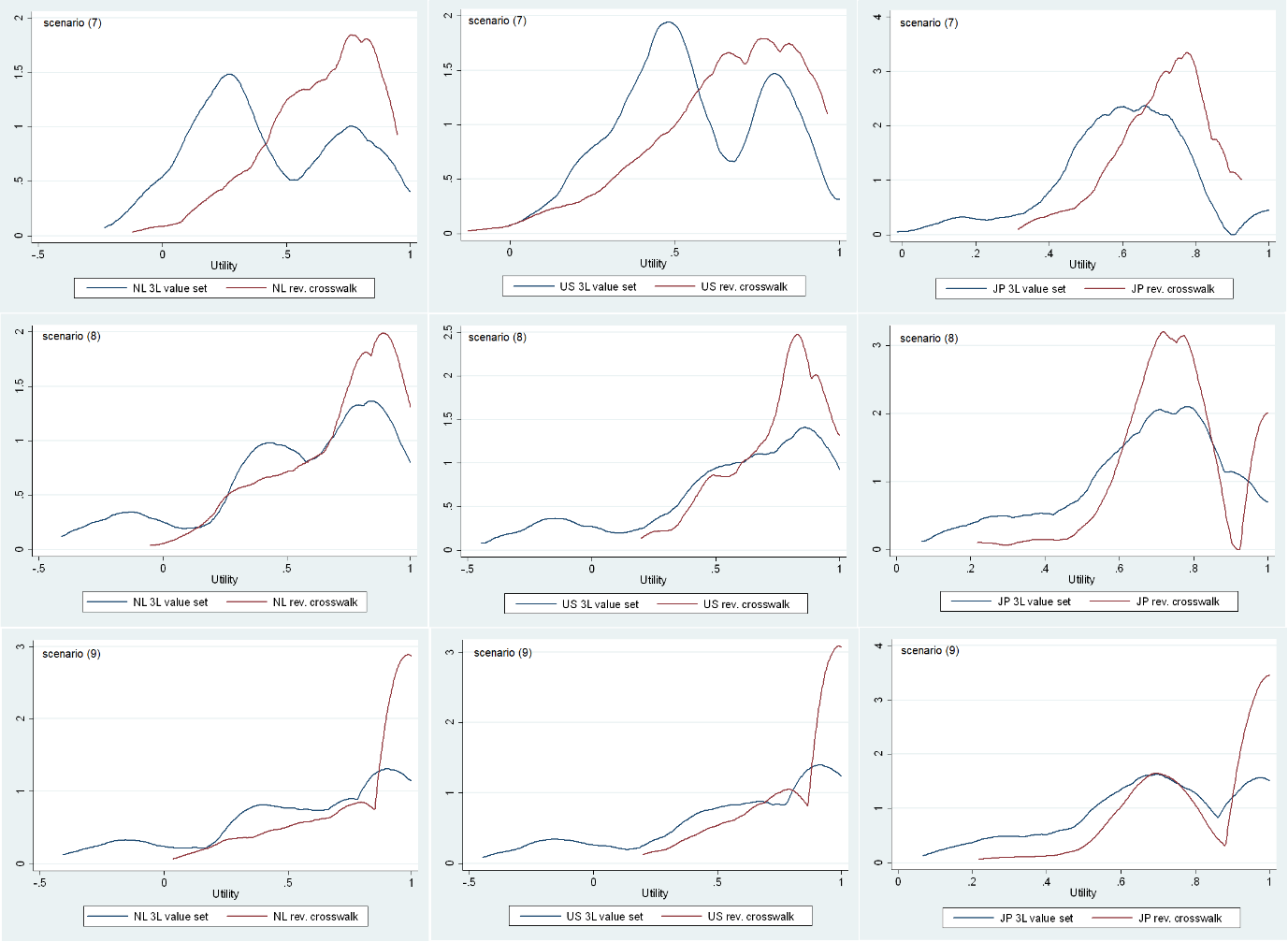
**

Scenario (7): severe depression and small treatment effect size.

Scenario (8): severe depression and medium treatment effect size.

Scenario (9): severe depression and large treatment effect size.

3L value set: EQ-5D-3L value set. rev. crosswalk: 5L to 3L crosswalk

NL: the Netherlands. US: the United States. JP: Japan.

**MILD LOW BACK PAIN**

**
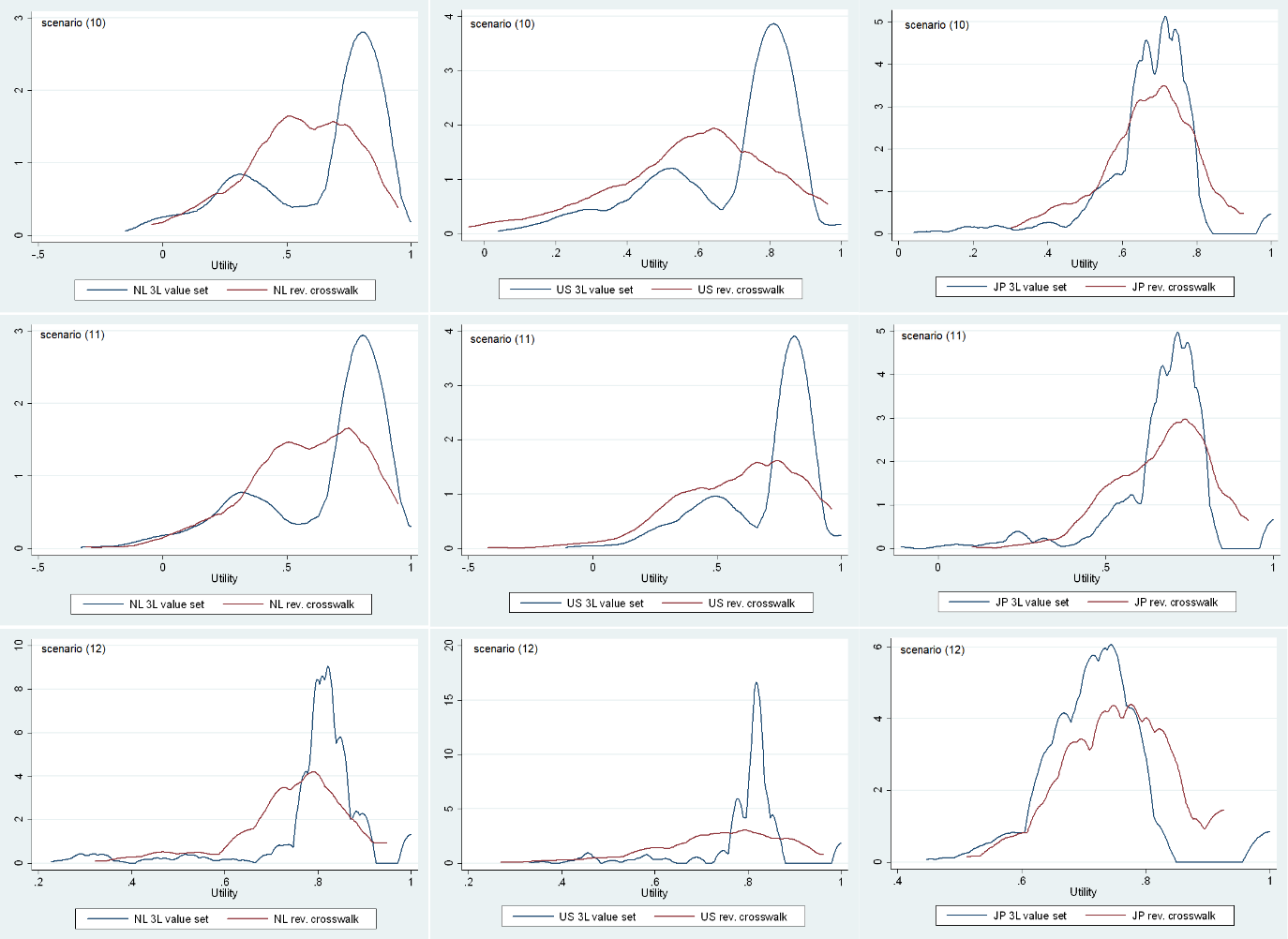
**

Scenario (10): mild low back pain and small treatment effect size.

Scenario (11): mild low back pain and medium treatment effect size.

Scenario (12): mild low back pain and large treatment effect size.

3L value set: EQ-5D-3L value set. rev. crosswalk: 5L to 3L crosswalk

NL: the Netherlands. US: the United States. JP: Japan.

**MODERATE LOW BACK PAIN**

**
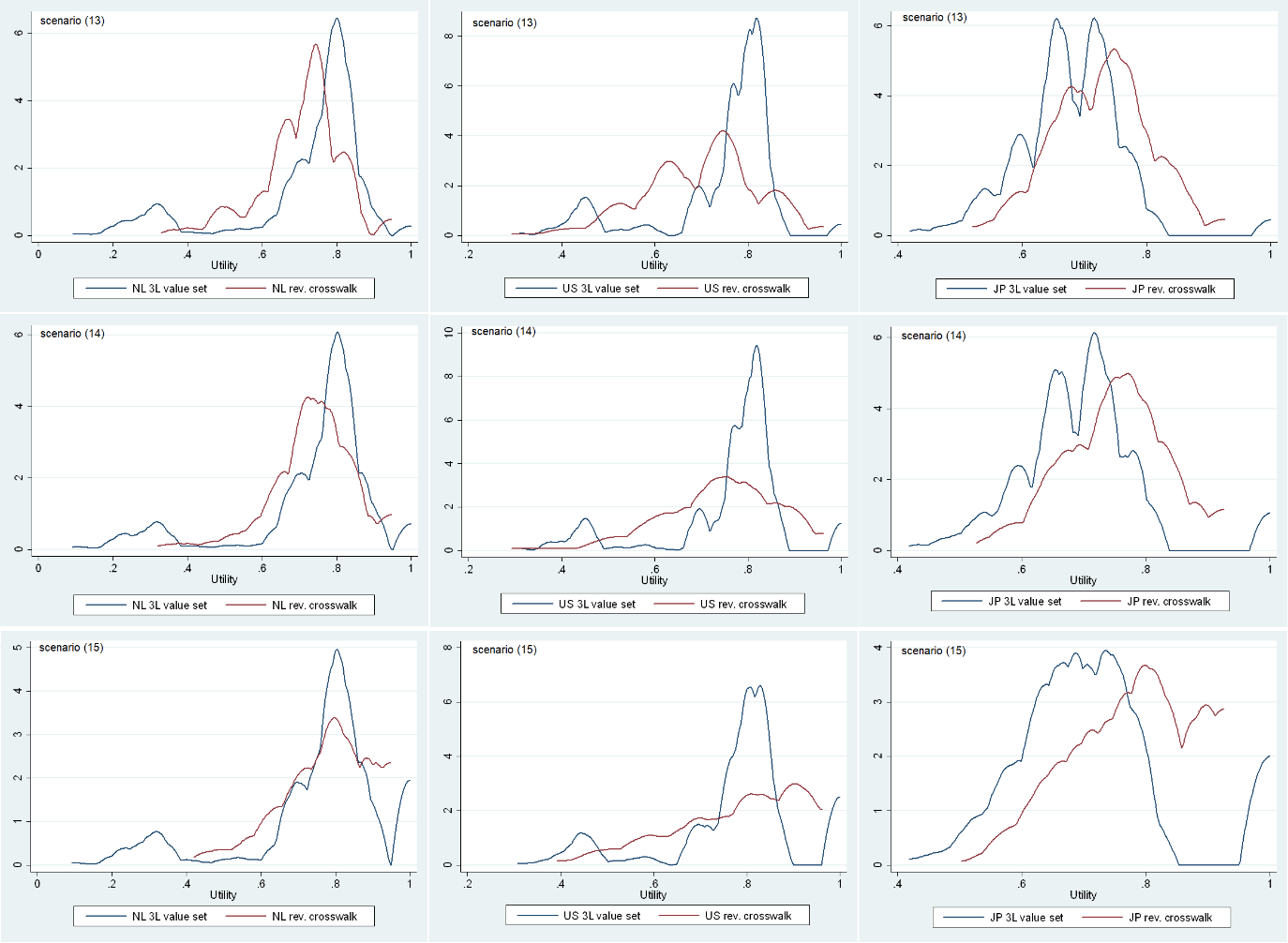
**

Scenario (13): moderate low back pain and small treatment effect size.

Scenario (14): moderate low back pain and medium treatment effect size.

Scenario (15): moderate low back pain and large treatment effect size.

3L value set: EQ-5D-3L value set. rev. crosswalk: 5L to 3L crosswalk

NL: the Netherlands. US: the United States. JP: Japan.

**SEVERE LOW BACK PAIN**

**
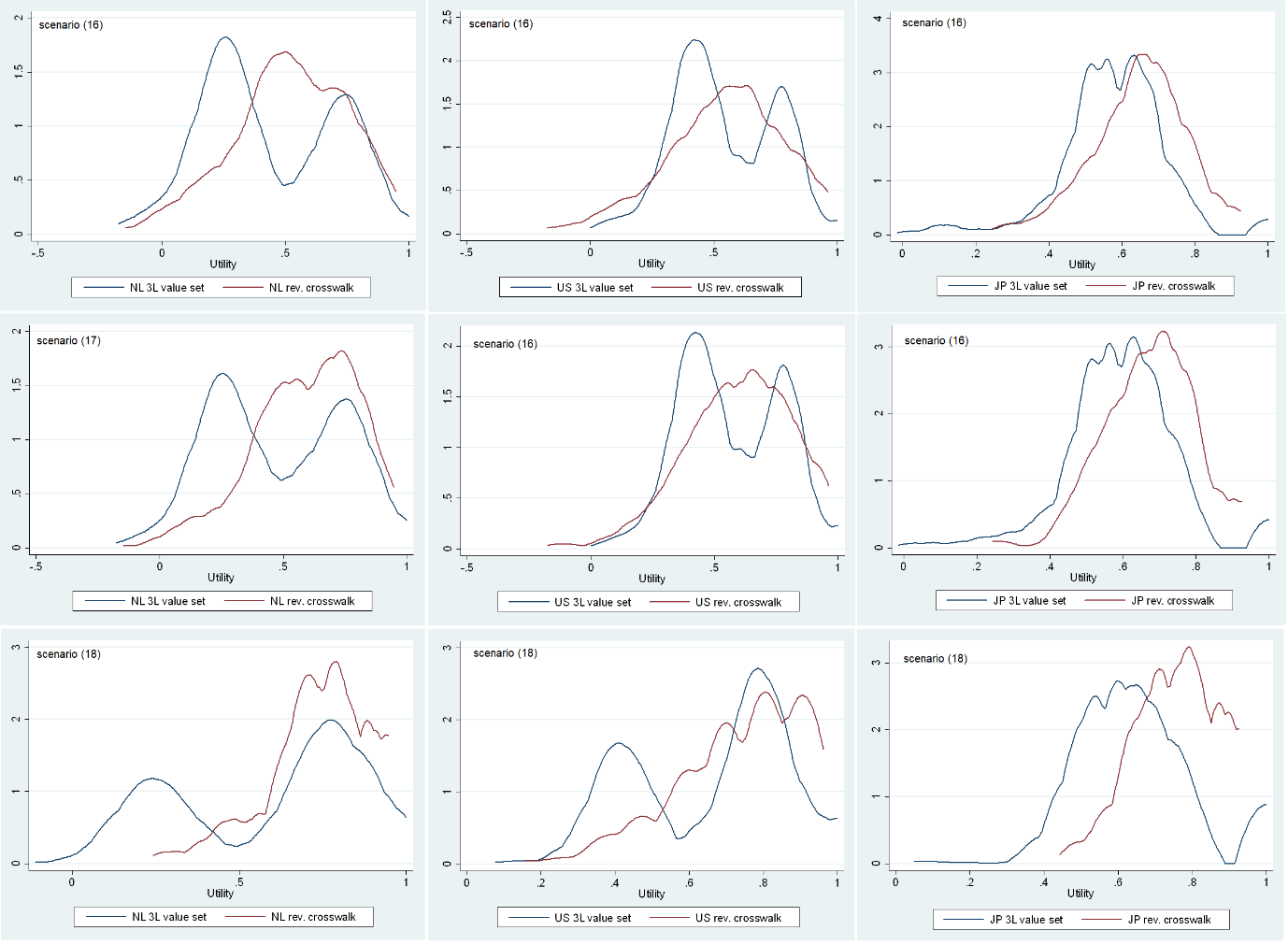
**

Scenario (16): severe low back pain and small treatment effect size.

Scenario (17): severe low back pain and medium treatment effect size.

Scenario (18): severe low back pain and large treatment effect size.

3L value set: EQ-5D-3L value set. rev. crosswalk: 5L to 3L crosswalk

NL: the Netherlands. US: the United States. JP: Japan.

**MILD OSTEOARTHRITIS**

**
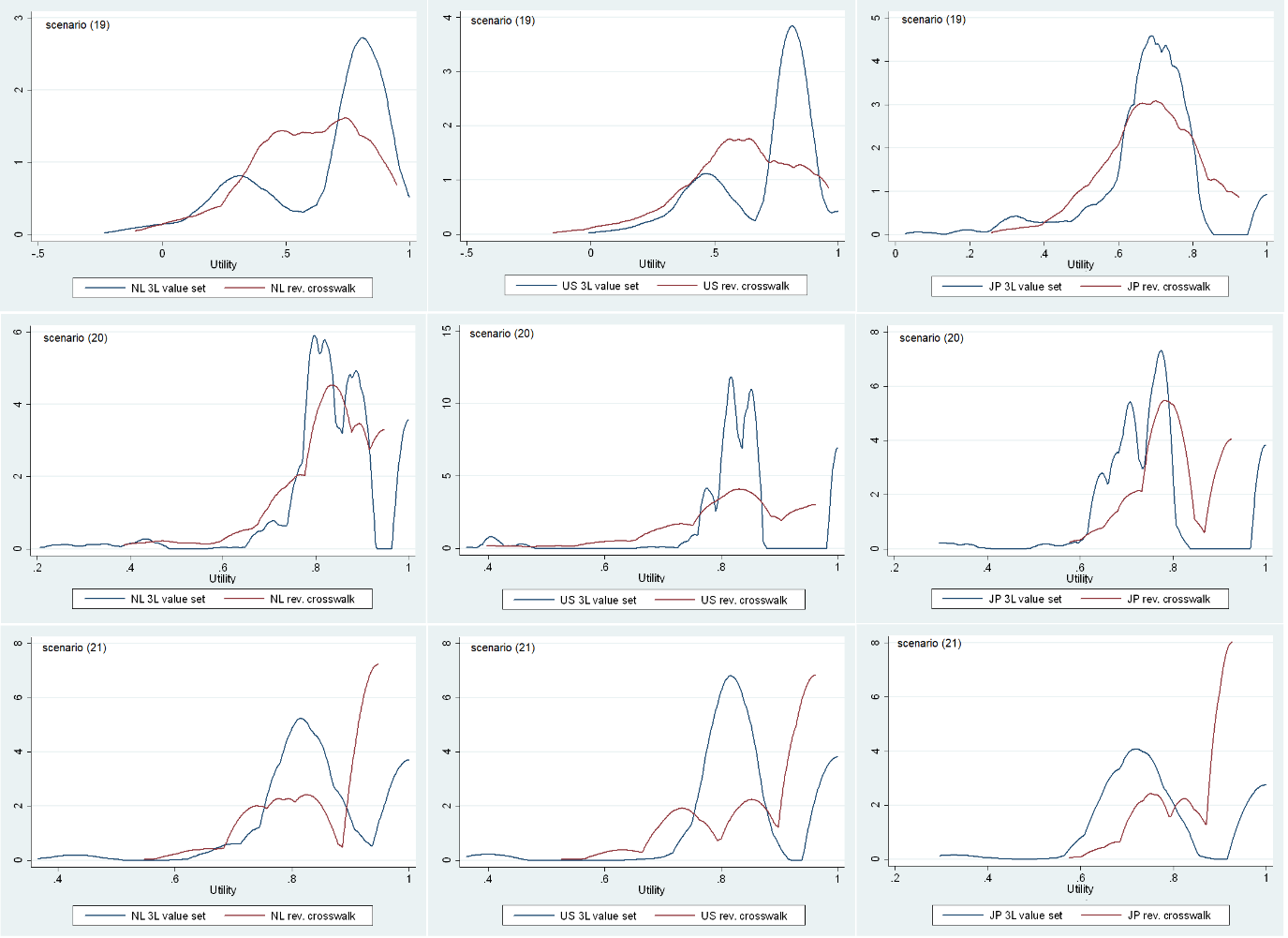
**

Scenario (19): mild osteoarthritis and small treatment effect size.

Scenario (20): mild osteoarthritis and medium treatment effect size.

Scenario (21): mild osteoarthritis pain and large treatment effect size.

3L value set: EQ-5D-3L value set. rev. crosswalk: 5L to 3L crosswalk

NL: the Netherlands. US: the United States. JP: Japan.

**MODERATE OSTEOARTHRITIS**

**
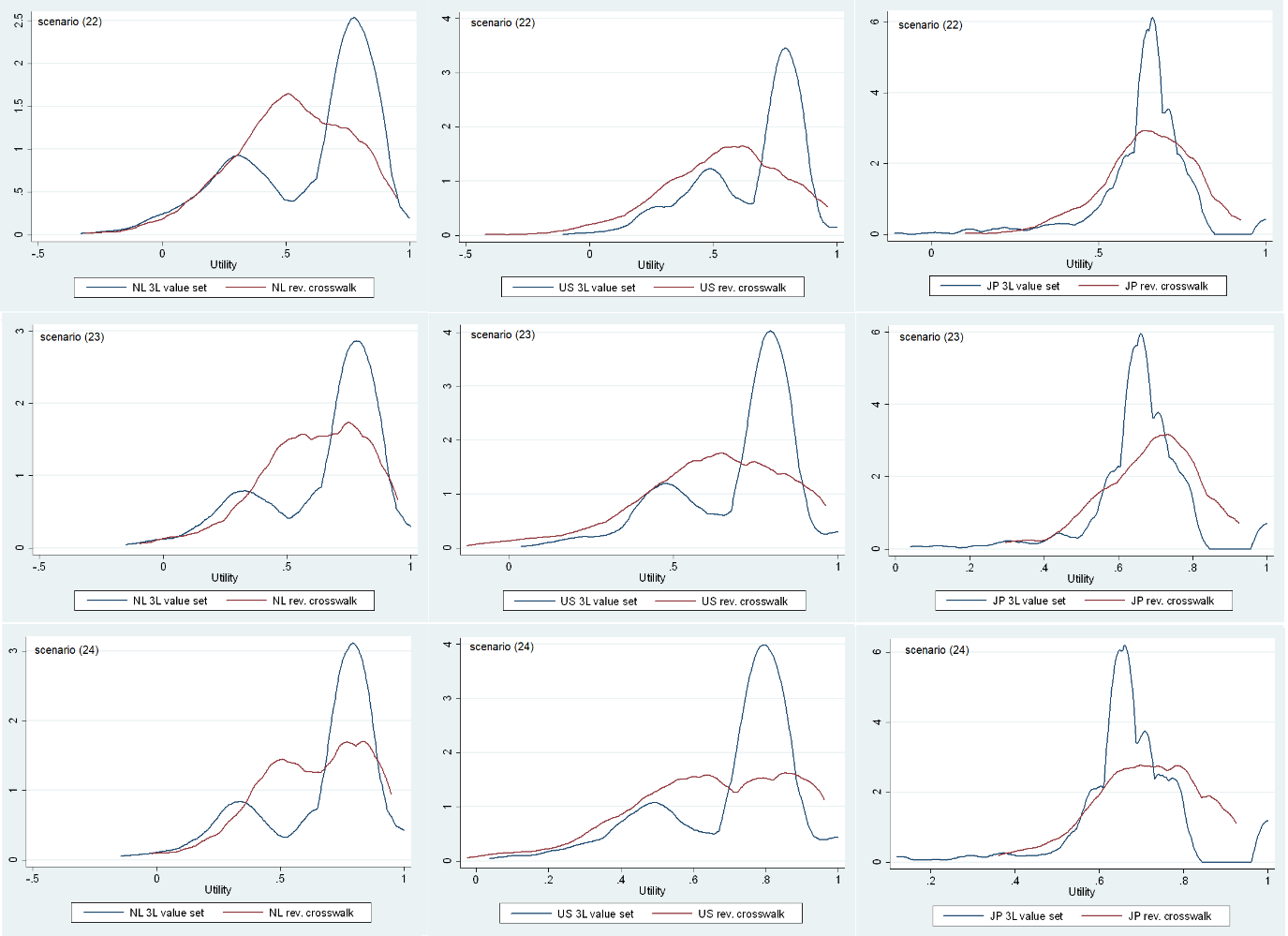
**

Scenario (22): moderate osteoarthritis and small treatment effect size.

Scenario (23): moderate osteoarthritis and medium treatment effect size.

Scenario (24): moderate osteoarthritis pain and large treatment effect size.

3L value set: EQ-5D-3L value set. rev. crosswalk: 5L to 3L crosswalk

NL: the Netherlands. US: the United States. JP: Japan.

**SEVERE OSTEOARTHRITIS**


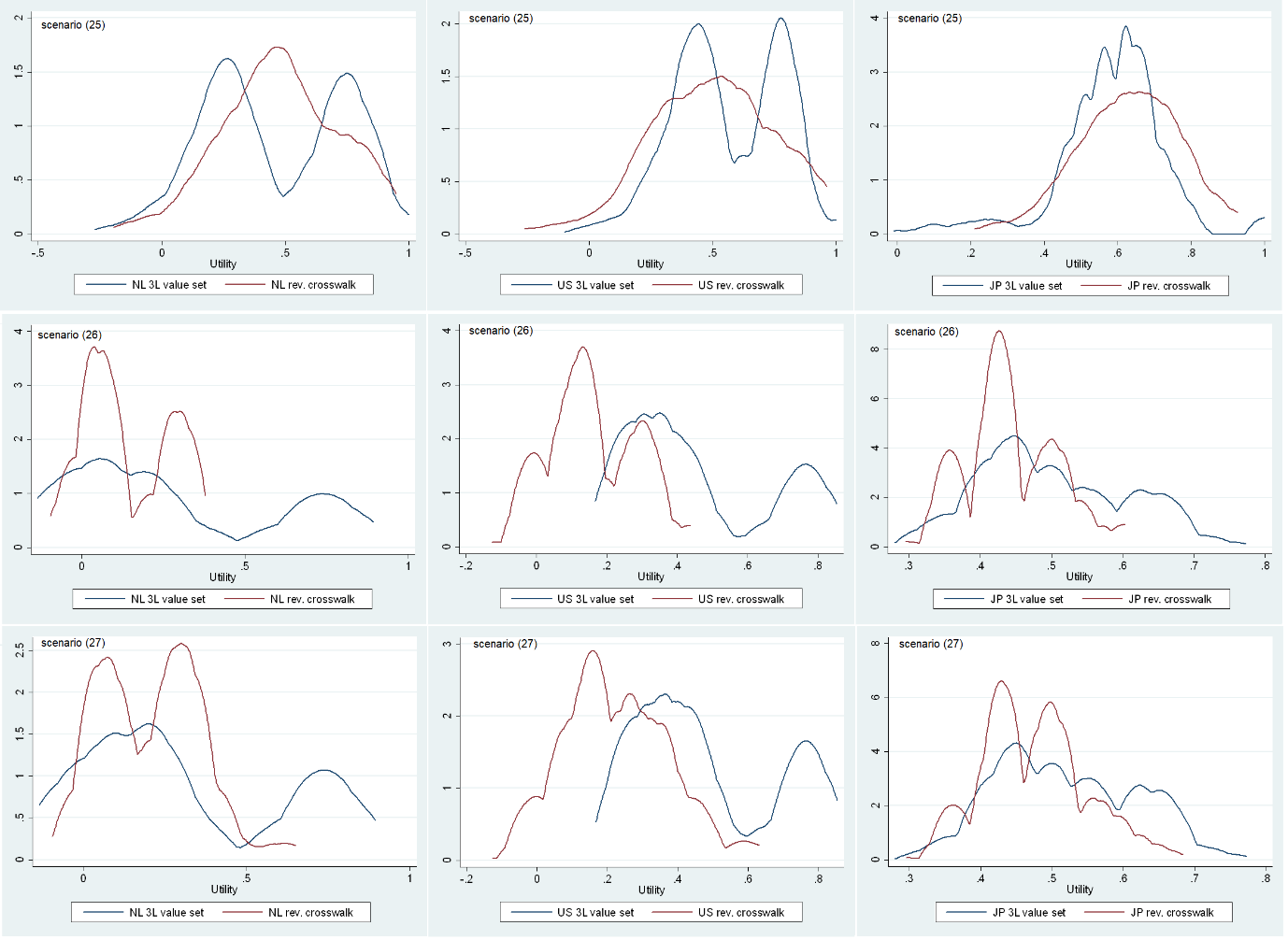


Scenario (25): severe osteoarthritis and small treatment effect size.

Scenario (26): severe osteoarthritis and medium treatment effect size.

Scenario (27): severe osteoarthritis pain and large treatment effect size.

3L value set: EQ-5D-3L value set. rev. crosswalk: 5L to 3L crosswalk

NL: the Netherlands. US: the United States. JP: Japan.

**MILD CANCER**


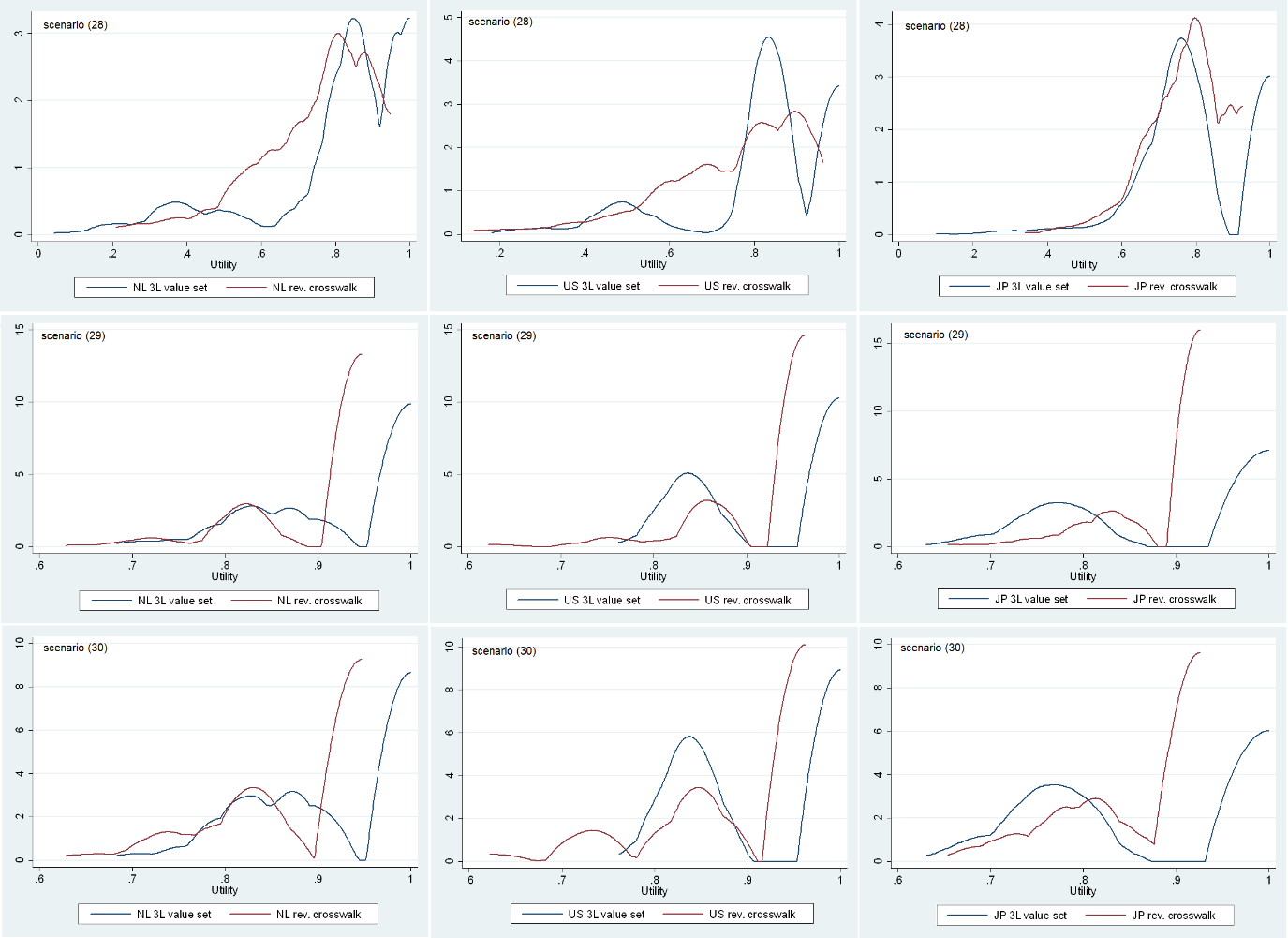


Scenario (28): mild cancer and small treatment effect size.

Scenario (29): mild cancer and medium treatment effect size.

Scenario (30): mild cancer and large treatment effect size.

3L value set: EQ-5D-3L value set. rev. crosswalk: 5L to 3L crosswalk

NL: the Netherlands. US: the United States. JP: Japan.

**MODERATE CANCER**


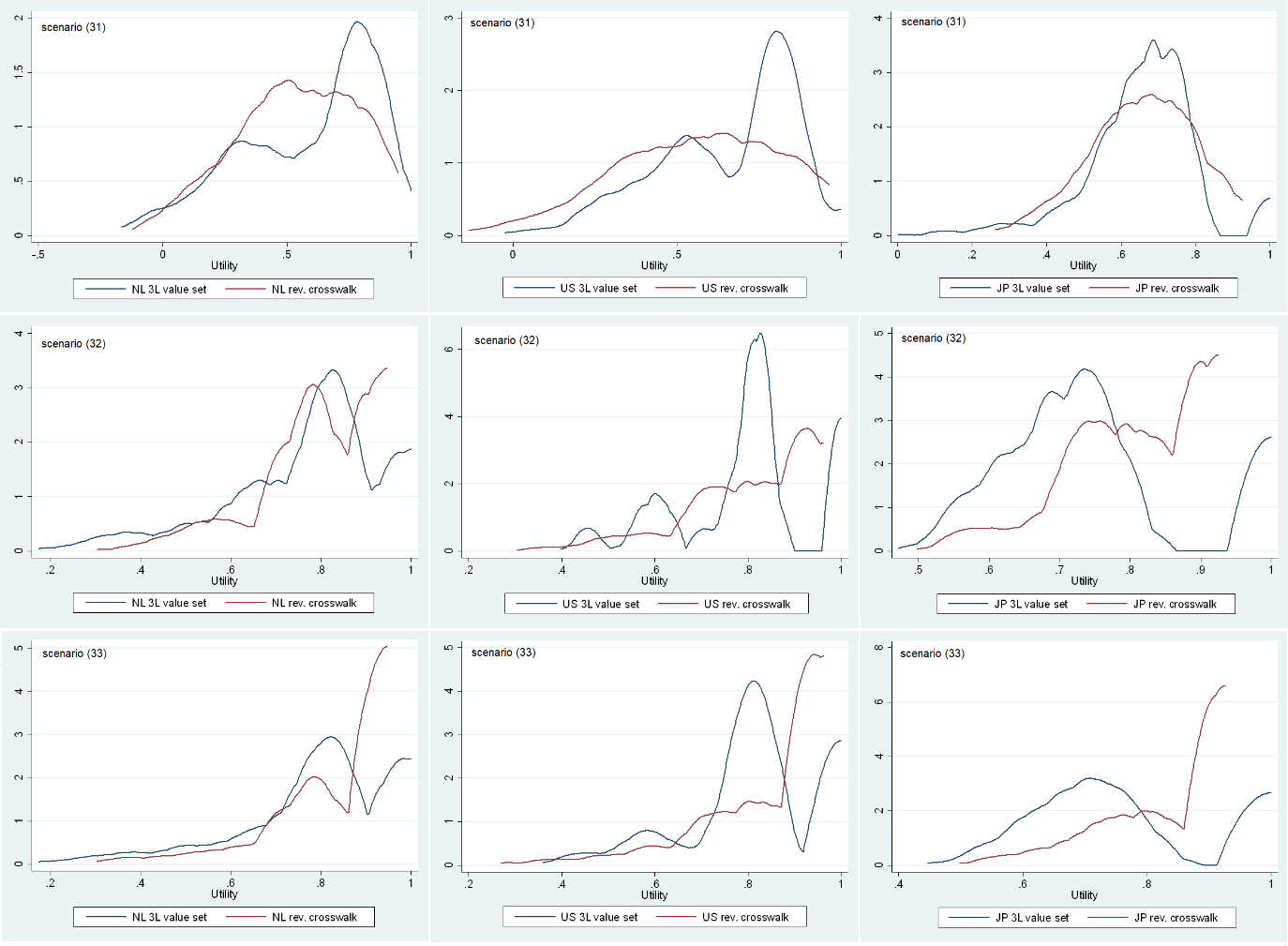


Scenario (31): moderate cancer and small treatment effect size.

Scenario (32): moderate cancer and medium treatment effect size.

Scenario (33): moderate cancer and large treatment effect size.

3L value set: EQ-5D-3L value set. rev. crosswalk: 5L to 3L crosswalk

NL: the Netherlands. US: the United States. JP: Japan.

**SEVERE CANCER**


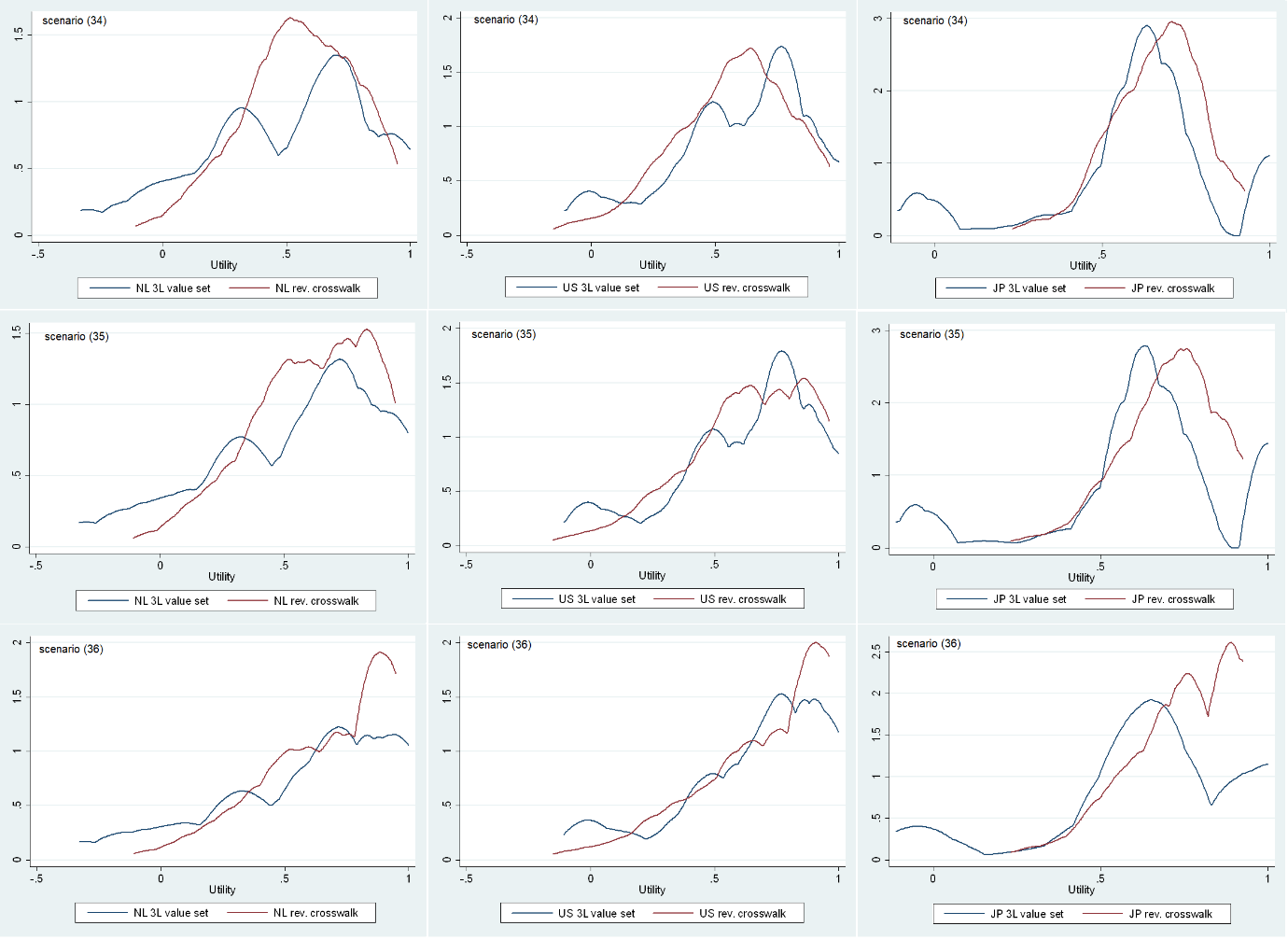


Scenario (34): severe cancer and small treatment effect size.

Scenario (35): severe cancer and medium treatment effect size.

Scenario (36): severe cancer and large treatment effect size.

3L value set: EQ-5D-3L value set. rev. crosswalk: 5L to 3L crosswalk

NL: the Netherlands. US: the United States. JP: Japan.

Kernel density histograms comparing utility distributions of the 5L value sets and 5L to 3L crosswalks.

**MILD DEPRESSION**

**
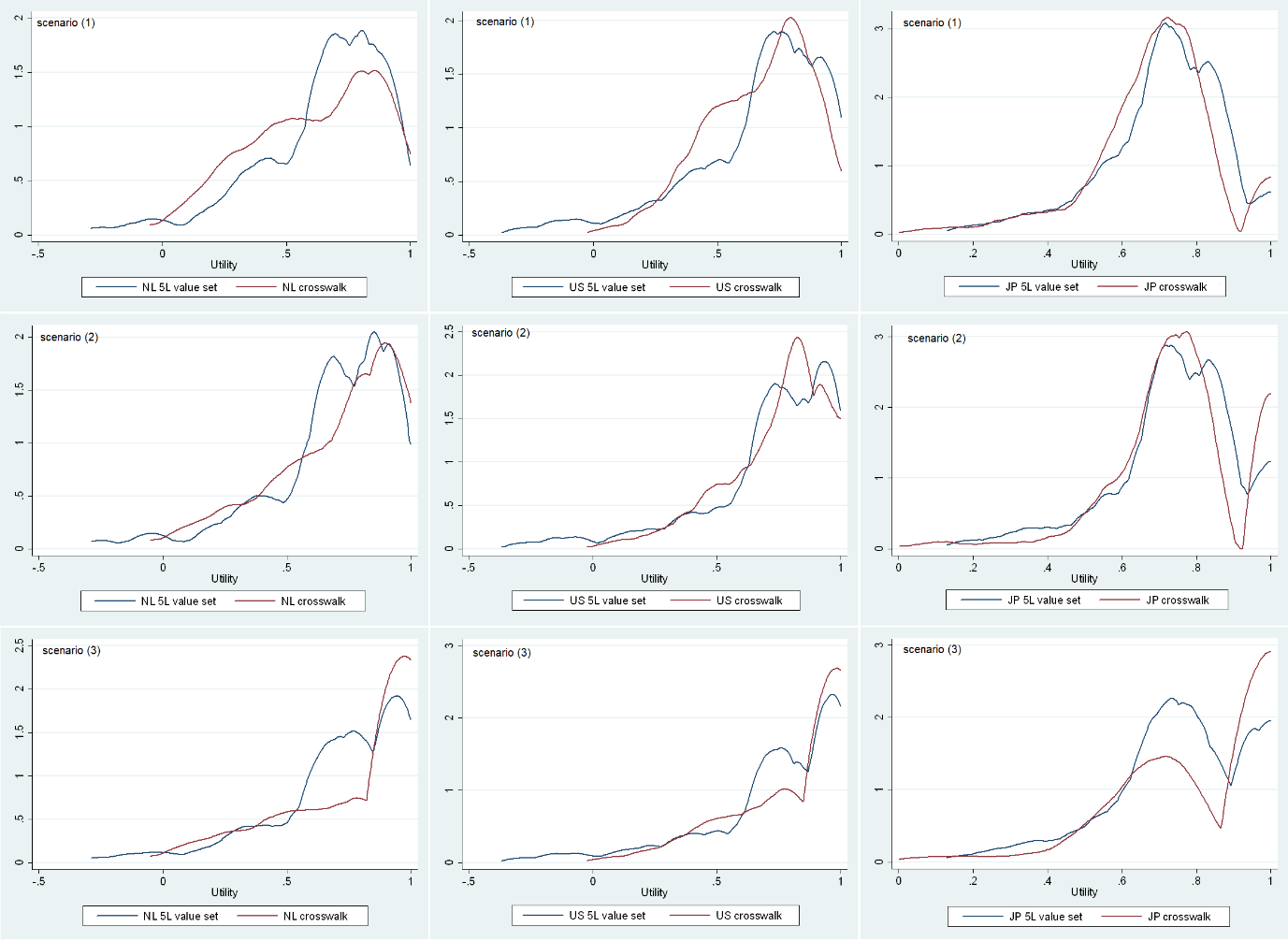
**

Scenario (1): mild depression and small treatment effect size.

Scenario (2): mild depression and medium treatment effect size.

Scenario (3): mild depression and large treatment effect size.

5L value set: EQ-5D-5L value set. crosswalk: 5L to 3L crosswalk

NL: the Netherlands. US: the United States. JP: Japan.

**MODERATE DEPRESSION**

**
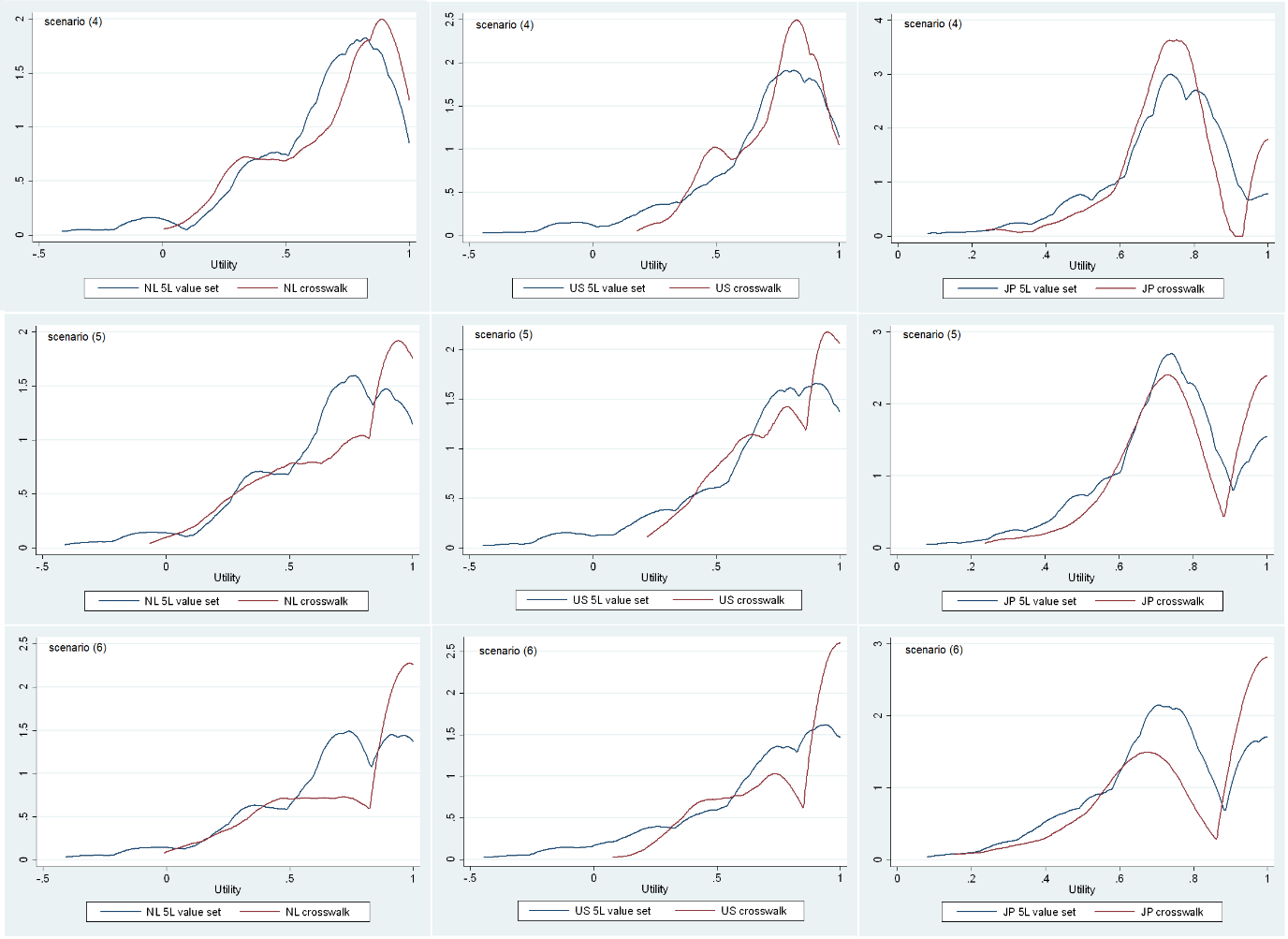
**

Scenario (4): moderate depression and small treatment effect size.

Scenario (5): moderate depression and medium treatment effect size.

Scenario (6): moderate depression and large treatment effect size.

5L value set: EQ-5D-5L value set.

NL: the Netherlands. US: the United States. JP: Japan.

**SEVERE DEPRESSION**

**
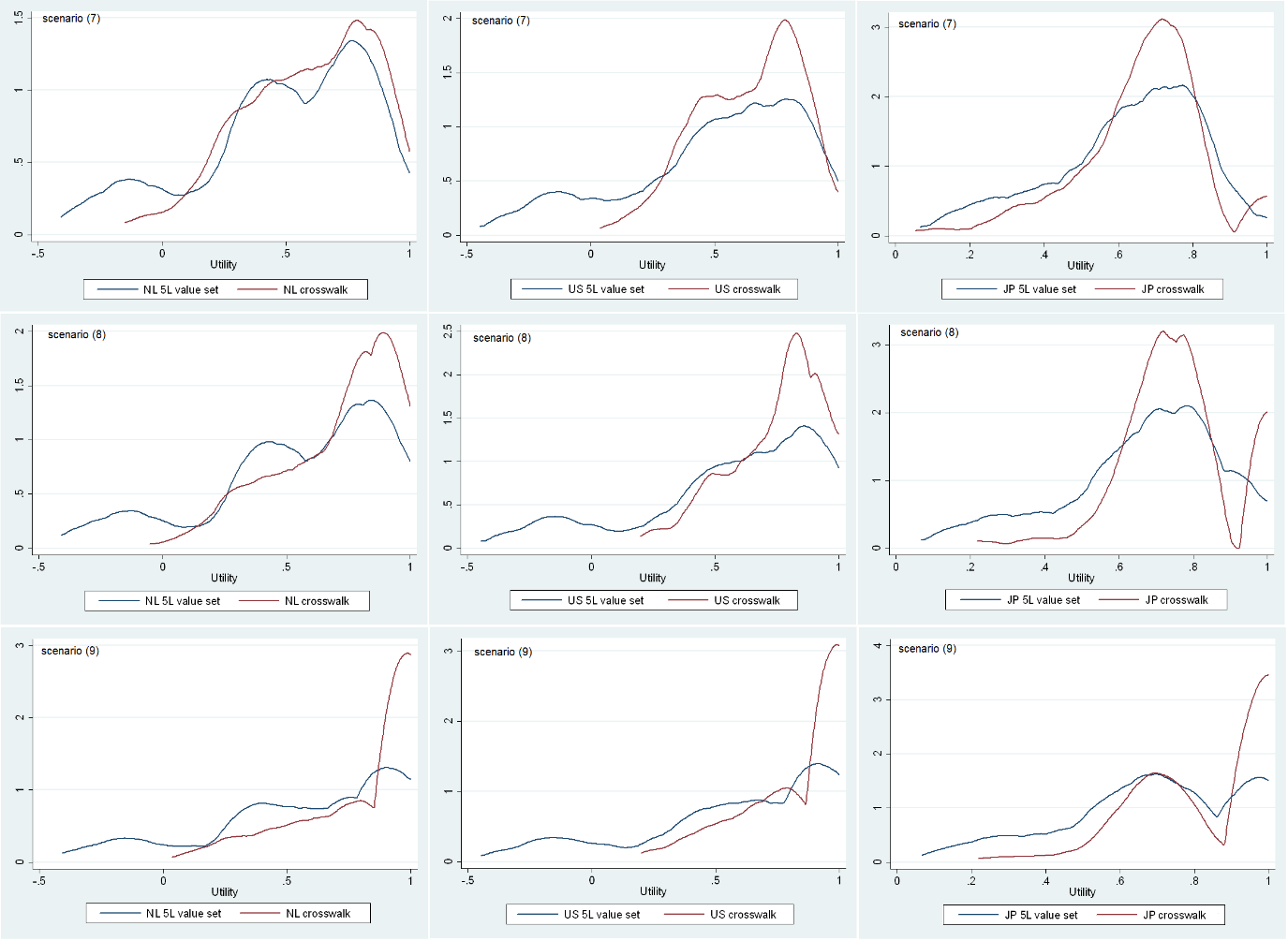
**

Scenario (7): severe depression and small treatment effect size.

Scenario (8): severe depression and medium treatment effect size.

Scenario (9): severe depression and large treatment effect size.

5L value set: EQ-5D-5L value set.

NL: the Netherlands. US: the United States. JP: Japan.

**MILD LOW BACK PAIN**


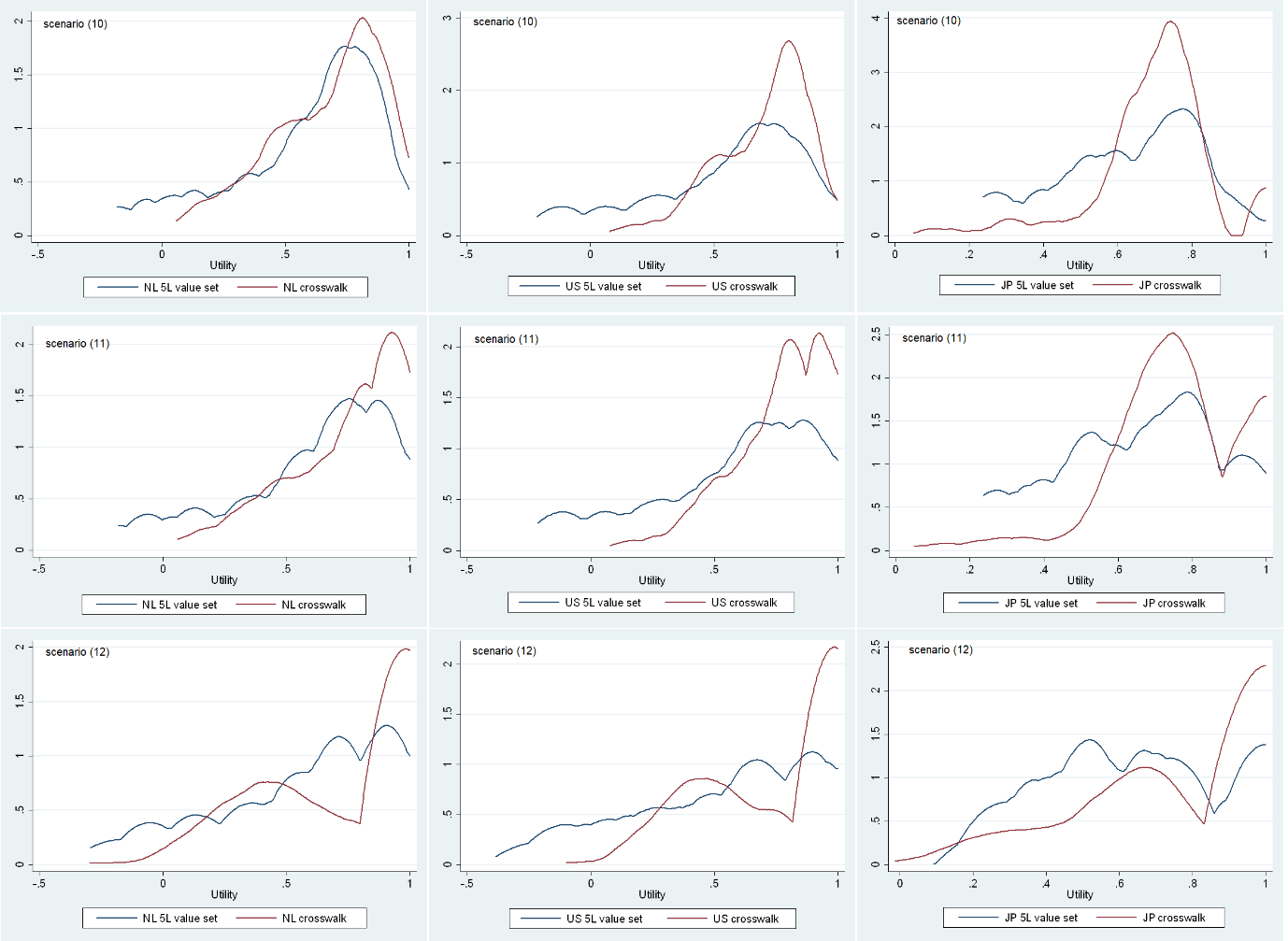


Scenario (10): mild low back pain and small treatment effect size.

Scenario (11): mild low back pain and medium treatment effect size.

Scenario (12): mild low back pain and large treatment effect size.

5L value set: EQ-5D-5L value set.

NL: the Netherlands. US: the United States. JP: Japan.

**MODERATE LOW BACK PAIN**

**
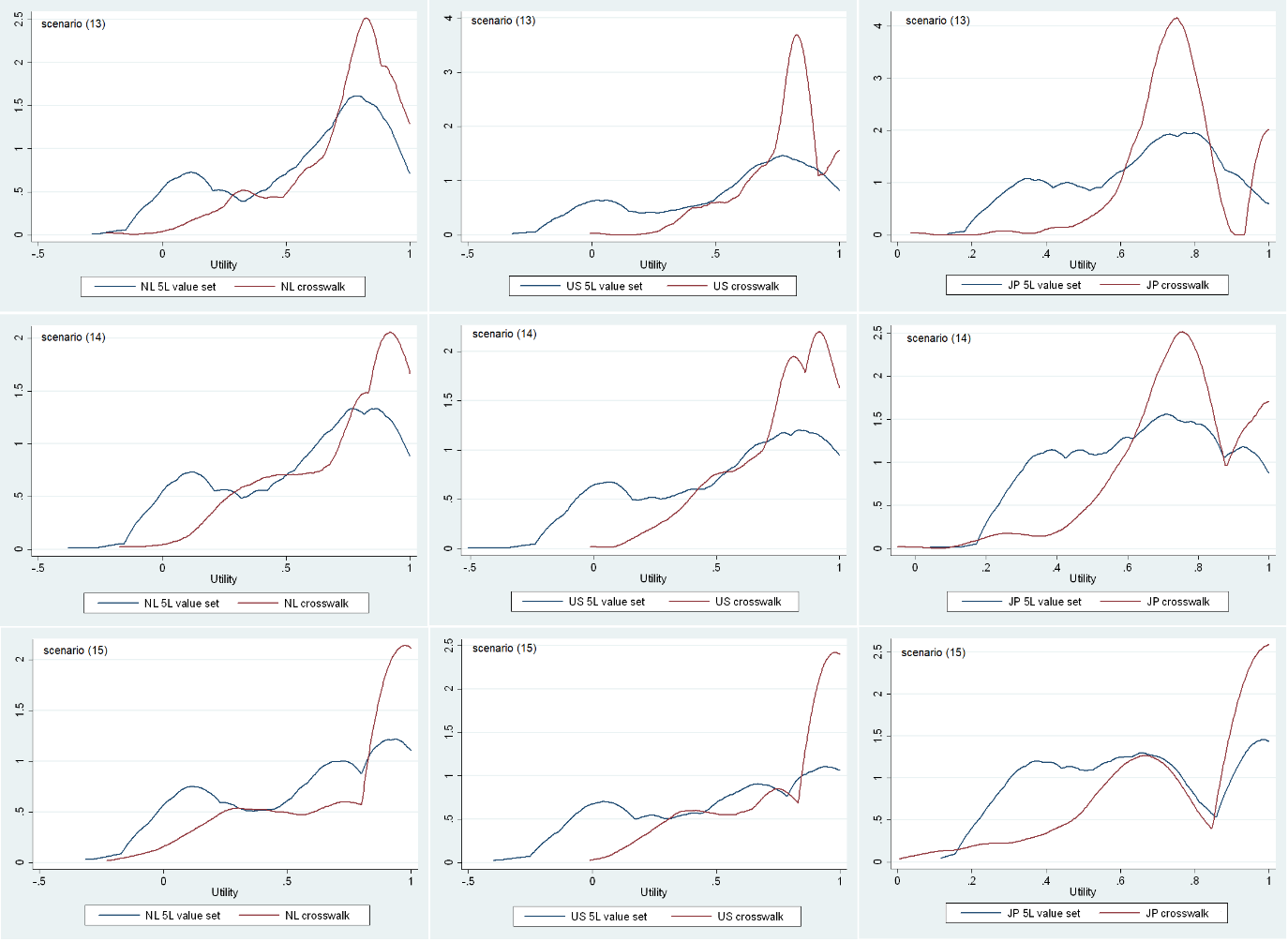
**

Scenario (13): moderate low back pain and small treatment effect size.

Scenario (14): moderate low back pain and medium treatment effect size.

Scenario (15): moderate low back pain and treatment large effect size.

5L value set: EQ-5D-5L value set.

NL: the Netherlands. US: the United States. JP: Japan.

**SEVERE LOW BACK PAIN**

**
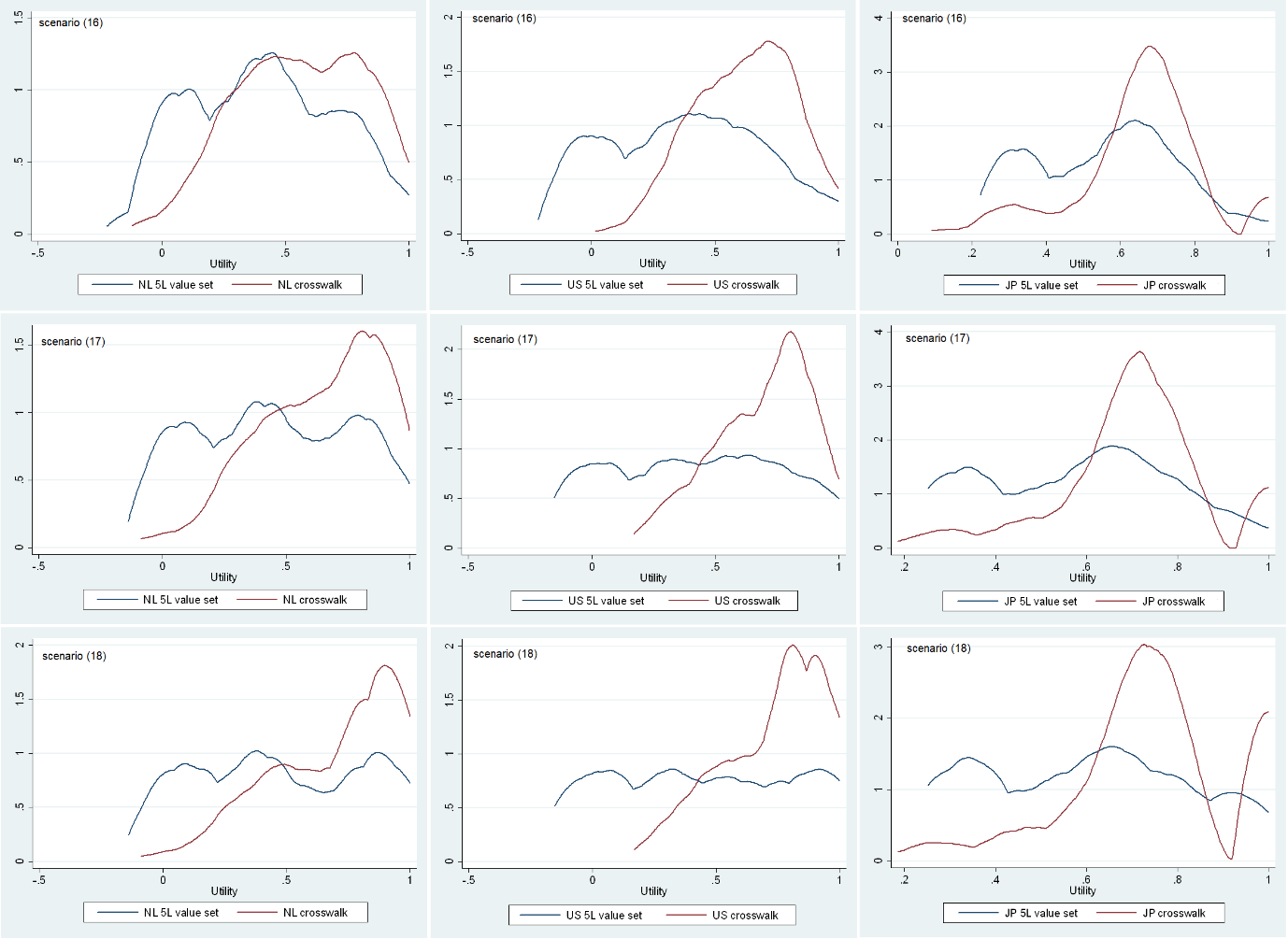
**

Scenario (16): severe low back pain and small treatment effect size.

Scenario (17): severe low back pain and medium treatment effect size.

Scenario (18): severe low back pain and large treatment effect size.

5L value set: EQ-5D-5L value set.

NL: the Netherlands. US: the United States. JP: Japan.

**MILD OSTEOARTHRITIS**

**
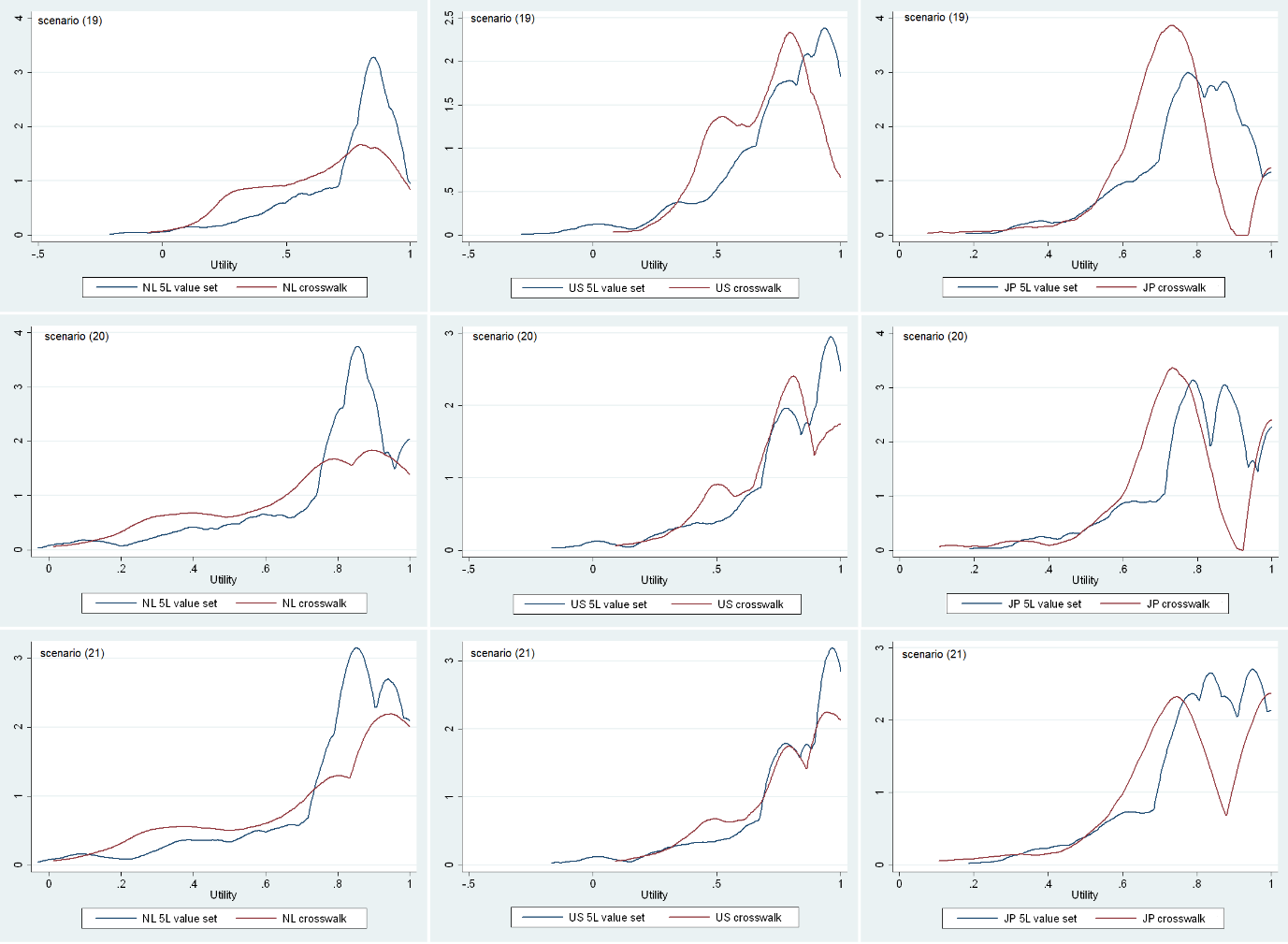
**

Scenario (19): mild osteoarthritis and small treatment effect size.

Scenario (20): mild osteoarthritis and medium treatment effect size.

Scenario (21): mild osteoarthritis pain and large treatment effect size.

5L value set: EQ-5D-5L value set.

NL: the Netherlands. US: the United States. JP: Japan.

**MODERATE OSTEOARTHRITIS**

**
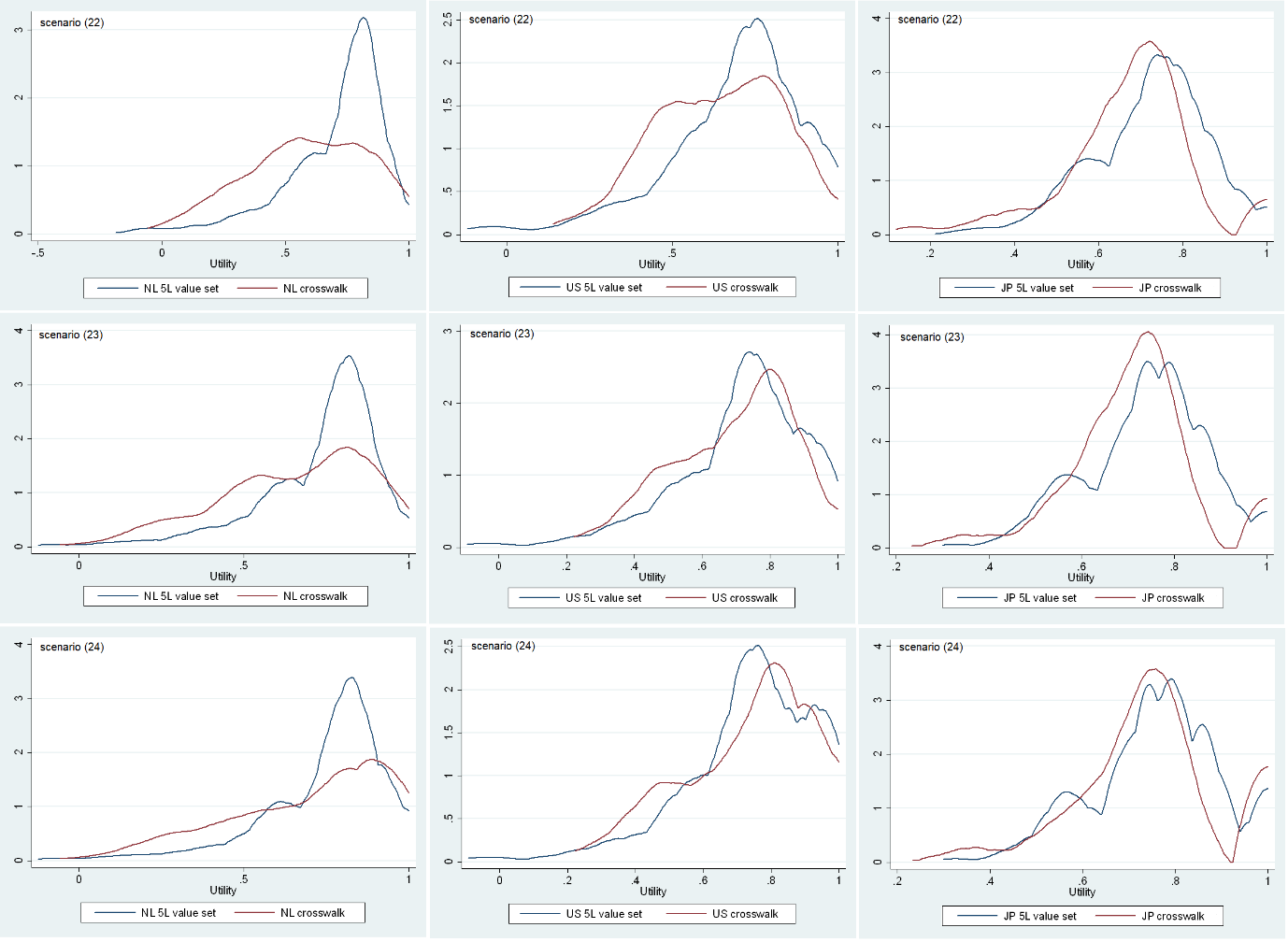
**

Scenario (22): moderate osteoarthritis and small treatment effect size.

Scenario (23): moderate osteoarthritis and medium treatment effect size.

Scenario (24): moderate osteoarthritis pain and large treatment effect size.

5L value set: EQ-5D-5L value set.

NL: the Netherlands. US: the United States. JP: Japan.

**SEVERE OSTEOARTHRITIS**

**
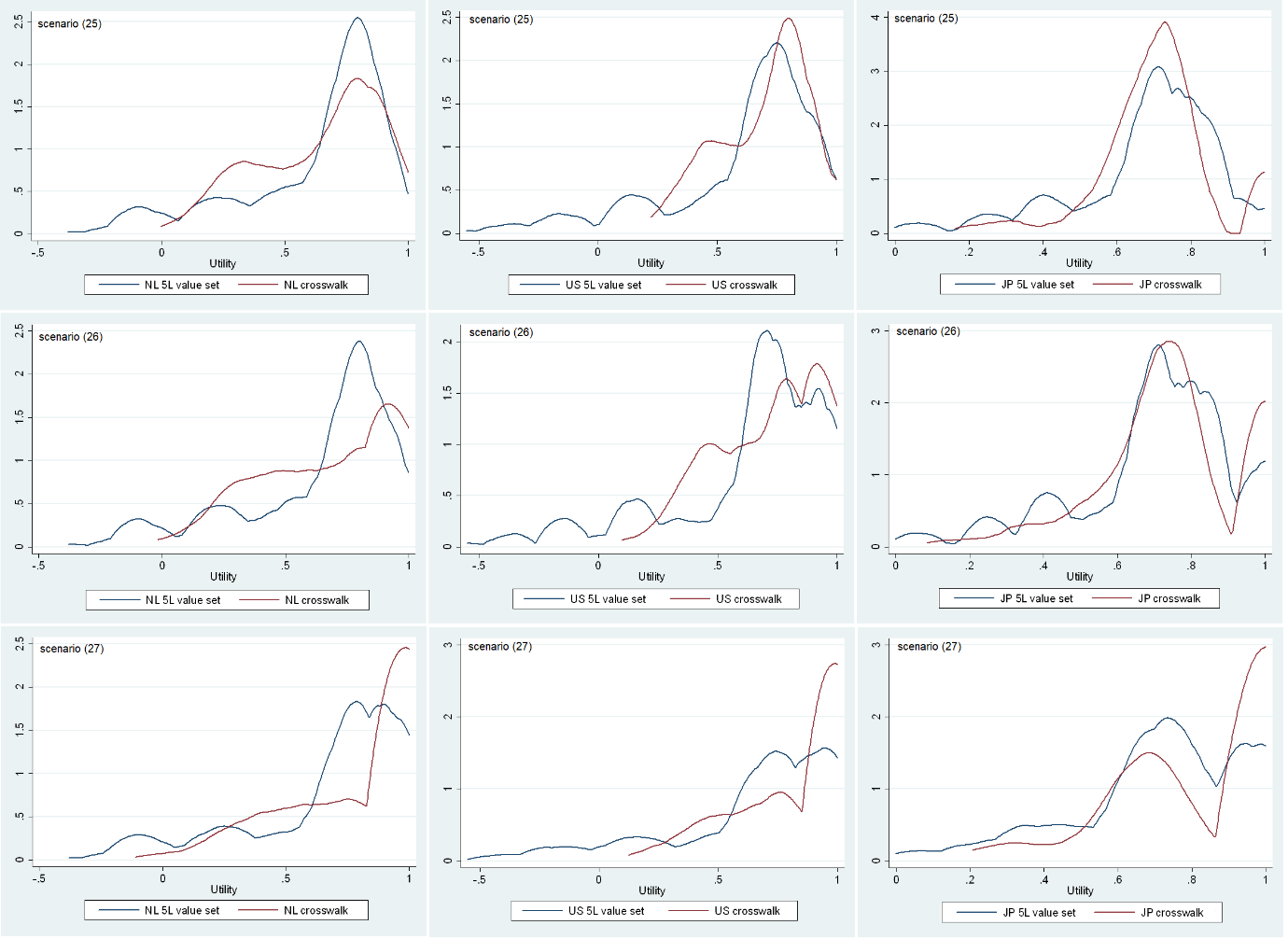
**

Scenario (25): severe osteoarthritis and small treatment effect size.

Scenario (26): severe osteoarthritis and medium treatment effect size.

Scenario (27): severe osteoarthritis pain and large treatment effect size.

5L value set: EQ-5D-5L value set.

NL: the Netherlands. US: the United States. JP: Japan.

**MILD CANCER**

**
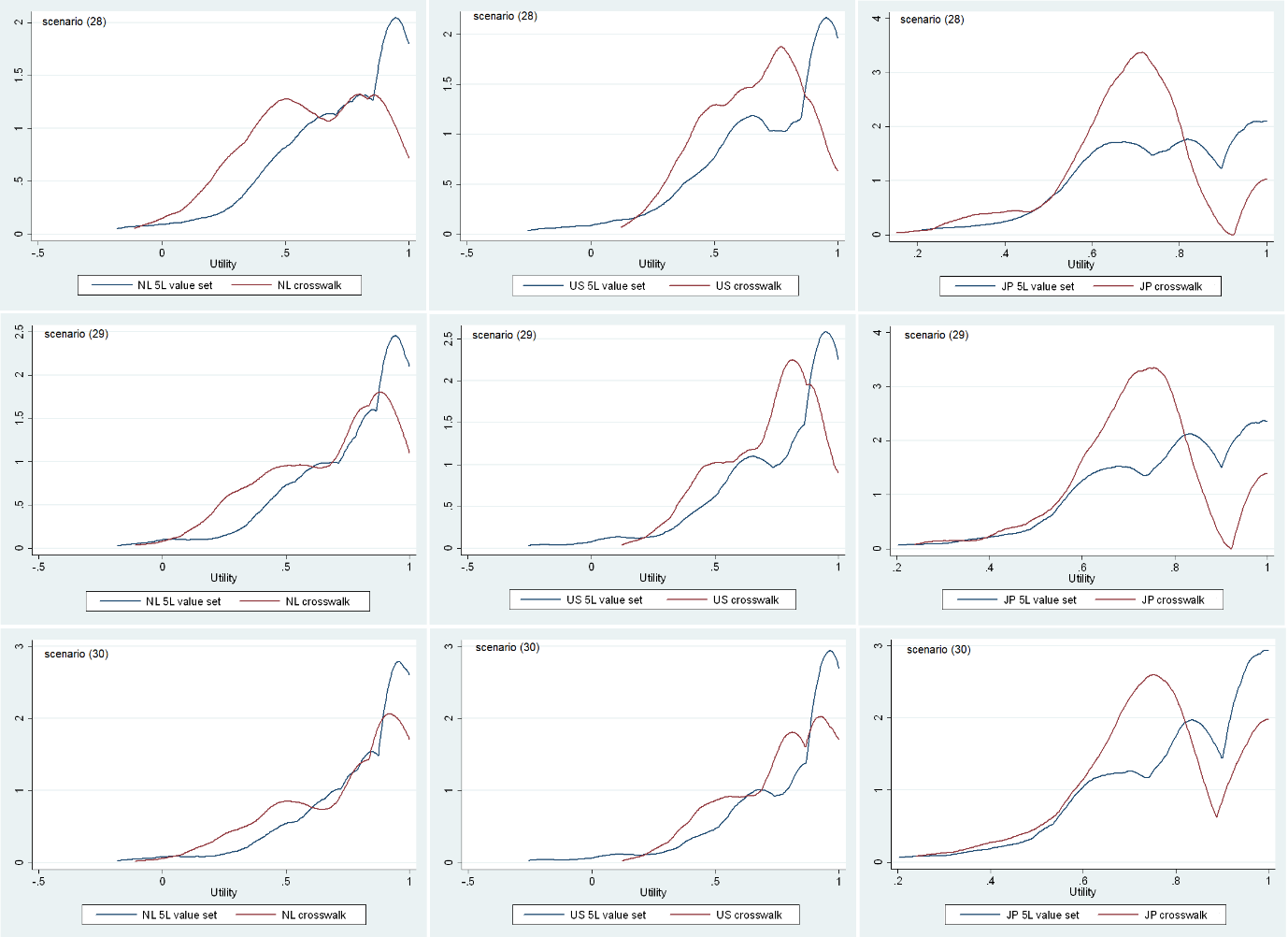
**

Scenario (28): mild cancer and small treatment effect size.

Scenario (29): mild cancer and medium treatment effect size.

Scenario (30): mild cancer and large treatment effect size.

5L value set: EQ-5D-5L value set.

NL: the Netherlands. US: the United States. JP: Japan.

**MODERATE CANCER**

**
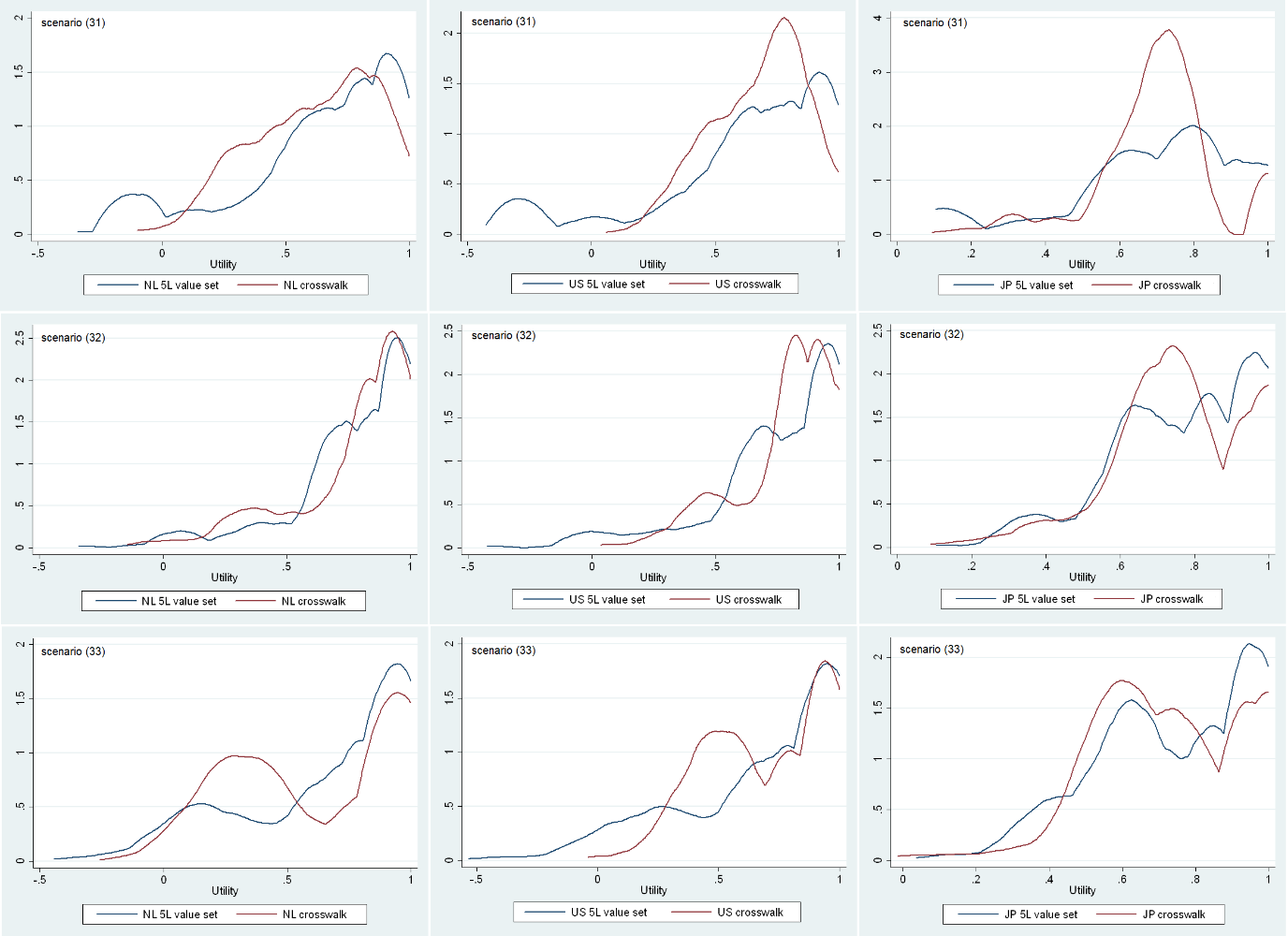
**

Scenario (31): moderate cancer and small treatment effect size.

Scenario (32): moderate cancer and medium treatment effect size.

Scenario (33): moderate cancer and large treatment effect size.

5L value set: EQ-5D-5L value set.

NL: the Netherlands. US: the United States. JP: Japan.

**SEVERE CANCER**

**
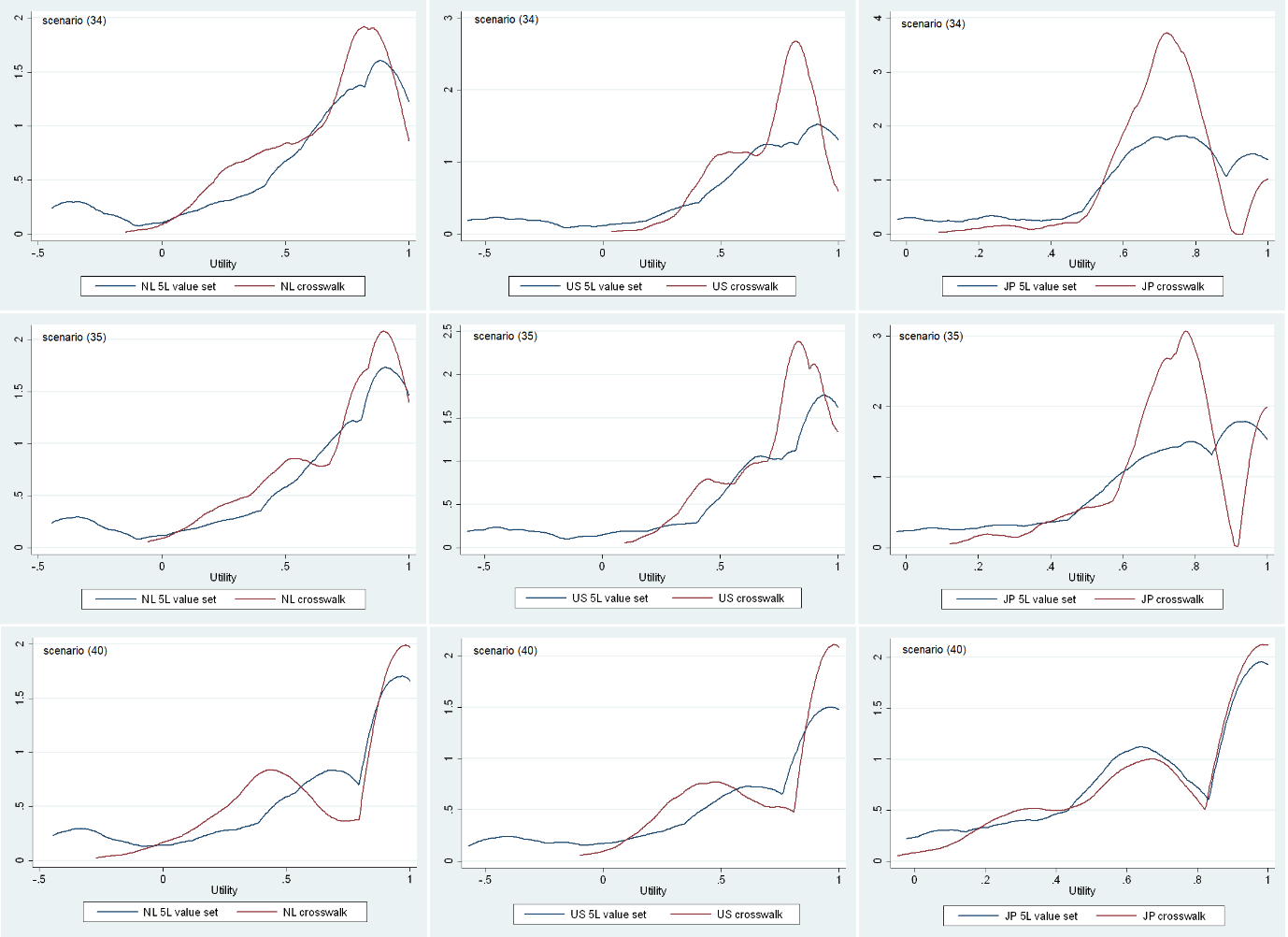
**

Scenario (34): severe cancer and small treatment effect size.

Scenario (35): severe cancer and medium treatment effect size.

Scenario (36): severe cancer and large treatment effect size.

5L value set: EQ-5D-5L value set.

NL: the Netherlands. US: the United States. JP: Japan.

**APPENDIX 4. Results of cost-utility analyses**

**Supplementary Table 4.1. Cost-utility analysis results for 3L value set and 3L to 5L crosswalk per country**

| **Country** | **Scoring method** | **Scenario** | **Patient population** | **Effect size** | **Incremental QALYs**  **IQ (95% CI)** | **Incremental costs**  **€** **(95% CI)** | **ICER**  **€/point** | **Distribution CE-plane (%)** | | | | **Probability of cost-effectiveness** | | | |
| --- | --- | --- | --- | --- | --- | --- | --- | --- | --- | --- | --- | --- | --- | --- | --- |
|  |  |  |  |  |  |  |  | **NE** | **SE** | **SW** | **NW** | **p_CE_(0)** | **p_CE_(20000)** | **p_CE_(30000)** | **p_CE_(50000)** |
| NL | 3L value set | (1) | Mild depression | Small | 0.05 (0.01; 0.09) | 214 (-266; 654) | 4656 | 81% | 17% | 0% | 2% | 0.173 | 0.911 | 0.949 | 0.968 |
|  | 3L to 5L crosswalk |  |  |  | 0.03 (-0.004; 0.06) | 214 (-266; 654) | 7284 | 80% | 17% | 1% | 2% | 0.173 | 0.813 | 0.883 | 0.933 |
| US | 3L value set |  |  |  | 0.02 (-0.01; 0.06) | 214 (-266; 654) | 8781 | 76% | 17% | 1% | 6% | 0.173 | 0.731 | **0.814** | **0.866** |
|  | 3L to 5L crosswalk |  |  |  | 0.02 (-0.02; 0.05) | 214 (-266; 654) | 13971 | 68% | 15% | 2% | 15% | 0.173 | 0.583 | **0.667** | **0.734** |
| JP | 3L value set |  |  |  | 0.02 (-0.01; 0.05) | 214 (-266; 654) | 11063 | 75% | 17% | 0% | 8% | 0.173 | 0.661 | 0.757 | **0.830** |
|  | 3L to 5L crosswalk |  |  |  | 0.0002 (-0.02; 0.02) | 214 (-266; 654) | 855681 | 40% | 10% | 7% | 42% | 0.173 | 0.242 | 0.290 | **0.358** |
| NL | 3L value set | (2) | Mild depression | Medium | 0.08 (0.05; 0.12) | -27 (-619; 458) | -327 | 47% | 53% | 0% | 0% | .526 | 1 | 1 | 1 |
|  | 3L to 5L crosswalk |  |  |  | 0.07 (0.04; 0.09) | -27 (-619; 458) | -407 | 47% | 53% | 0% | 0% | .526 | 1 | 1 | 1 |
| US | 3L value set |  |  |  | 0.05 (0.03; 0.08) | -27 (-619; 458) | -503 | 47% | 53% | 0% | 0% | .526 | .997 | .998 | .999 |
|  | 3L to 5L crosswalk |  |  |  | 0.06 (0.03; 0.09) | -27 (-619; 458) | -459 | 47% | 53% | 0% | 0% | .526 | .999 | .999 | 1 |
| JP | 3L value set |  |  |  | 0.06 (0.03; 0.08) | -27 (-619; 458) | -481 | 47% | 53% | 0% | 0% | .526 | .999 | 1 | 1 |
|  | 3L to 5L crosswalk |  |  |  | 0.04 (0.02; 0.05) | -27 (-619; 458) | -737 | 47% | 53% | 0% | 0% | .526 | .988 | .998 | 1 |
| NL | 3L value set | (3) | Mild depression | Large | 0.14 (0.10; 0.17) | -18 (-478; 442) | -139 | 47% | 53% | 0% | 0% | 0.530 | 1 | 1 | 1 |
|  | 3L to 5L crosswalk |  |  |  | 0.11 (0.08; 0.13) | -18 (-478; 442) | -178 | 47% | 53% | 0% | 0% | 0.530 | 1 | 1 | 1 |
| US | 3L value set |  |  |  | 0.09 (0.07; 0.12) | -18 (-478; 442) | -203 | 47% | 53% | 0% | 0% | 0.530 | 1 | 1 | 1 |
|  | 3L to 5L crosswalk |  |  |  | 0.09 (0.07; 0.12) | -18 (-478; 442) | -205 | 47% | 53% | 0% | 0% | 0.530 | 1 | 1 | 1 |
| JP | 3L value set |  |  |  | 0.09 (0.07; 0.12) | -18 (-478; 442) | -203 | 47% | 53% | 0% | 0% | 0.530 | 1 | 1 | 1 |
|  | 3L to 5L crosswalk |  |  |  | 0.06 (0.04; 0.07) | -18 (-478; 442) | -330 | 47% | 53% | 0% | 0% | 0.530 | 1 | 1 | 1 |
| NL | 3L value set | (4) | Moderate depression | Small | 0.04 (0.0002; 0.08) | 165 (-391; 651) | 4129 | 73% | 24% | 0% | 3% | 0.246 | 0.893 | 0.930 | 0.960 |
|  | 3L to 5L crosswalk |  |  |  | 0.04 (0.01; 0.07) | 165 (-391; 651) | 4520 | 75% | 24% | 0% | 1% | 0.246 | 0.908 | 0.958 | 0.978 |
| US | 3L value set |  |  |  | 0.04 (0.01; 0.07) | 165 (-391; 651) | 4613 | 74% | 24% | 1% | 1% | 0.246 | 0.895 | 0.945 | 0.972 |
|  | 3L to 5L crosswalk |  |  |  | 0.04 (0.01; 0.07) | 165 (-391; 651) | 4289 | 75% | 24% | 0% | 1% | 0.246 | 0.922 | 0.963 | 0.981 |
| JP | 3L value set |  |  |  | 0.02 (-0.003; 0.05) | 165 (-391; 651) | 7193 | 72% | 24% | 0% | 4% | 0.246 | 0.773 | 0.860 | 0.910 |
|  | 3L to 5L crosswalk |  |  |  | 0.03 (0.01; 0.04) | 165 (-391; 651) | 6440 | 75% | 24% | 0% | 1% | 0.246 | 0.844 | 0.935 | 0.977 |
| NL | 3L value set | (5) | Moderate depression | Medium | 0.09 (0.05; 0.12) | -152 (-724; 427) | -1753 | 29% | 71% | 0% | 0% | 0.709 | 0.999 | 1 | 1 |
|  | 3L to 5L crosswalk |  |  |  | 0.09 (0.07; 0.12) | -152 (-724; 427) | -1619 | 29% | 71% | 0% | 0% | 0.709 | 1 | 1 | 1 |
| US | 3L value set |  |  |  | 0.06 (0.03; 0.08) | -152 (-724; 427) | -2565 | 29% | 71% | 0% | 0% | 0.709 | 0.999 | 0.999 | 0.999 |
|  | 3L to 5L crosswalk |  |  |  | 0.09 (0.06; 0.11) | -152 (-724; 427) | -1710 | 29% | 71% | 0% | 0% | 0.709 | 1 | 1 | 1 |
| JP | 3L value set |  |  |  | 0.07 (0.04; 0.09) | -152 (-724; 427) | -2301 | 29% | 71% | 0% | 0% | 0.709 | 0.999 | 0.999 | 0.999 |
|  | 3L to 5L crosswalk |  |  |  | 0.07 (0.05; 0.08) | -152 (-724; 427) | -2260 | 29% | 71% | 0% | 0% | 0.709 | 1 | 1 | 1 |
| NL | 3L value set | (6) | Moderate depression | Large | 0.13 (0.10; 0.16) | 351 (-30; 738) | 2705 | 96% | 4% | 0% | 0% | 0.036 | 1 | 1 | 1 |
|  | 3L to 5L crosswalk |  |  |  | 0.12 (0.10; 0.15) | 351 (-30; 738) | 2853 | 96% | 4% | 0% | 0% | 0.036 | 1 | 1 | 1 |
| US | 3L value set |  |  |  | 0.11 (0.08; 0.13) | 351 (-30; 738) | 3240 | 96% | 4% | 0% | 0% | 0.036 | 1 | 1 | 1 |
|  | 3L to 5L crosswalk |  |  |  | 0.12 (0.1; 0.15) | 351 (-30; 738) | 2901 | 96% | 4% | 0% | 0% | 0.036 | 1 | 1 | 1 |
| JP | 3L value set |  |  |  | 0.14 (0.11; 0.16) | 351 (-30; 738) | 2583 | 96% | 4% | 0% | 0% | 0.036 | 1 | 1 | 1 |
|  | 3L to 5L crosswalk |  |  |  | 0.10 (0.08; 0.11) | 351 (-30; 738) | 3526 | 96% | 4% | 0% | 0% | 0.036 | 1 | 1 | 1 |
| NL | 3L value set | (7) | Severe depression | Small | 0.05 (0.01; 0.10) | 96 (-310; 475) | 1805 | 69% | 31% | 0% | 0% | 0.307 | 0.975 | 0.984 | 0.991 |
|  | 3L to 5L crosswalk |  |  |  | 0.04 (0.01; 0.08) | 96 (-310; 475) | 2208 | 69% | 31% | 0% | 0% | 0.307 | 0.975 | 0.987 | 0.993 |
| US | 3L value set |  |  |  | 0.03 (0.002; 0.07) | 96 (-310; 475) | 2757 | 68% | 31% | 0% | 1% | 0.307 | 0.930 | 0.953 | 0.968 |
|  | 3L to 5L crosswalk |  |  |  | 0.04 (0.01; 0.07) | 96 (-310; 475) | 2405 | 69% | 31% | 0% | 0% | 0.307 | 0.965 | 0.980 | 0.987 |
| JP | 3L value set |  |  |  | 0.03 (-0.0004; 0.05) | 96 (-310; 475) | 3497 | 67% | 31% | 0% | 2% | 0.307 | 0.896 | 0.932 | 0.955 |
|  | 3L to 5L crosswalk |  |  |  | 0.02 (0.01; 0.04) | 96 (-310; 475) | 4060 | 69% | 31% | 0% | 0% | 0.307 | 0.912 | 0.963 | 0.984 |
| NL | 3L value set | (8) | Severe depression | Medium | 0.11 (0.06; 0.15) | -157 (-624; 292) | -1434 | 26% | 74% | 0% | 0% | 0.740 | 1 | 1 | 1 |
|  | 3L to 5L crosswalk |  |  |  | 0.08 (0.04; 0.11) | -157 (-624; 292) | -1980 | 26% | 74% | 0% | 0% | 0.740 | 1 | 1 | 1 |
| US | 3L value set |  |  |  | 0.08 (0.05; 0.12) | -157 (-624; 292) | -1878 | 26% | 74% | 0% | 0% | 0.740 | 1 | 1 | 1 |
|  | 3L to 5L crosswalk |  |  |  | 0.08 (0.04; 0.11) | -157 (-624; 292) | -2076 | 26% | 74% | 0% | 0% | 0.740 | 1 | 1 | 1 |
| JP | 3L value set |  |  |  | 0.08 (0.04; 0.11) | -157 (-624; 292) | -2059 | 26% | 74% | 0% | 0% | 0.740 | 1 | 1 | 1 |
|  | 3L to 5L crosswalk |  |  |  | 0.05 (0.03; 0.07) | -157 (-624; 292) | -3031 | 26% | 74% | 0% | 0% | 0.740 | 1 | 1 | 1 |
| NL | 3L value set | (9) | Severe depression | Large | 0.24 (0.20; 0.29) | 123 (-331; 592) | 503 | 68% | 32% | 0% | 0% | 0.319 | 1 | 1 | 1 |
|  | 3L to 5L crosswalk |  |  |  | 0.18 (0.15; 0.22) | 123 (-331; 592) | 672 | 68% | 32% | 0% | 0% | 0.319 | 1 | 1 | 1 |
| US | 3L value set |  |  |  | 0.17 (0.14; 0.21) | 123 (-331; 592) | 713 | 68% | 32% | 0% | 0% | 0.319 | 1 | 1 | 1 |
|  | 3L to 5L crosswalk |  |  |  | 0.17 (0.13; 0.21) | 123 (-331; 592) | 729 | 68% | 32% | 0% | 0% | 0.319 | 1 | 1 | 1 |
| JP | 3L value set |  |  |  | 0.14 (0.11; 0.17) | 123 (-331; 592) | 895 | 68% | 32% | 0% | 0% | 0.319 | 1 | 1 | 1 |
|  | 3L to 5L crosswalk |  |  |  | 0.11 (0.09; 0.13) | 123 (-331; 592) | 1161 | 68% | 32% | 0% | 0% | 0.319 | 1 | 1 | 1 |

**Cont. Supplementary Table 4.1. Cost-utility analysis results for 3L value set and 3L to 5L crosswalk per country**

| **Country** | **Scoring method** | **Scenario** | **Patient population** | **Effect size** | **Incremental QALYs**  **IQ (95% CI)** | **Incremental costs**  **€ (95% CI)** | **ICER**  **€/point** | **Distribution CE-plane (%)** | | | | **Probability of cost-effectiveness** | | | |
| --- | --- | --- | --- | --- | --- | --- | --- | --- | --- | --- | --- | --- | --- | --- | --- |
|  |  |  |  |  |  |  |  | **NE** | **SE** | **SW** | **NW** | **p_CE_(0)** | **p_CE_(20000)** | **p_CE_(30000)** | **p_CE_(50000)** |
| NL | 3L value set | (10) | Mild low back pain | Small | 0.03 (-0.01; 0.06) | 131 (-386; 652) | 4680 | 65% | 30% | 1% | 4% | 0.310 | 0.810 | 0.867 | 0.903 |
|  | 3L to 5L crosswalk |  |  |  | 0.03 (-0.0002; 0.05) | 131 (-386; 652) | 5057 | 67% | 31% | 0% | 2% | 0.310 | 0.827 | 0.893 | 0.936 |
| US | 3L value set |  |  |  | 0.02 (-0.004; 0.05) | 131 (-386; 652) | 6159 | 64% | 31% | 1% | 4% | 0.310 | **0.770** | 0.833 | 0.886 |
|  | 3L to 5L crosswalk |  |  |  | 0.04 (0.02; 0.07) | 131 (-386; 652) | 3069 | 69% | 31% | 0% | 0% | 0.310 | **0.960** | 0.986 | 0.997 |
| JP | 3L value set |  |  |  | 0.02 (0.002; 0.04) | 131 (-386; 652) | 6193 | 67% | 31% | 1% | 1% | 0.310 | **0.798** | 0.877 | 0.942 |
|  | 3L to 5L crosswalk |  |  |  | 0.03 (0.02; 0.05) | 131 (-386; 652) | 4158 | 69% | 31% | 0% | 0% | 0.310 | **0.934** | 0.984 | 0.999 |
| NL | 3L value set | (11) | Mild low back pain | Medium | 0.10 (0.06; 0.13) | 215 (-291; 713) | 2263 | 80% | 20% | 0% | 0% | 0.197 | 1 | 1 | 1 |
|  | 3L to 5L crosswalk |  |  |  | 0.07 (0.04; 0.10) | 215 (-291; 713) | 3024 | 80% | 20% | 0% | 0% | 0.197 | 1 | 1 | 1 |
| US | 3L value set |  |  |  | 0.07 (0.04; 0.10) | 215 (-291; 713) | 3083 | 80% | 20% | 0% | 0% | 0.197 | 0.998 | 0.999 | 1 |
|  | 3L to 5L crosswalk |  |  |  | 0.08 (0.05; 0.11) | 215 (-291; 713) | 2551 | 80% | 20% | 0% | 0% | 0.197 | 1 | 1 | 1 |
| JP | 3L value set |  |  |  | 0.06 (0.03; 0.08) | 215 (-291; 713) | 3907 | 80% | 20% | 0% | 0% | 0.197 | 0.991 | 0.999 | 1 |
|  | 3L to 5L crosswalk |  |  |  | 0.05 (0.03; 0.07) | 215 (-291; 713) | 4158 | 80% | 20% | 0% | 0% | 0.197 | 0.994 | 1 | 1 |
| NL | 3L value set | (12) | Mild low back pain | Large | 0.09 (0.07; 0.12) | 380 (-90; 900) | 4115 | 94% | 6% | 0% | 0% | 0.063 | 1 | 1 | 1 |
|  | 3L to 5L crosswalk |  |  |  | 0.07 (0.05; 0.09) | 380 (-90; 900) | 5476 | 94% | 6% | 0% | 0% | 0.063 | 1 | 1 | 1 |
| US | 3L value set |  |  |  | 0.07 (0.06; 0.09) | 380 (-90; 900) | 5148 | 94% | 6% | 0% | 0% | 0.063 | 1 | 1 | 1 |
|  | 3L to 5L crosswalk |  |  |  | 0.09 (0.08; 0.12) | 380 (-90; 900) | 4010 | 94% | 6% | 0% | 0% | 0.063 | 1 | 1 | 1 |
| JP | 3L value set |  |  |  | 0.06 (0.04; 0.07) | 380 (-90; 900) | 6663 | 94% | 6% | 0% | 0% | 0.063 | 0.996 | 1 | 1 |
|  | 3L to 5L crosswalk |  |  |  | 0.06 (0.05; 0.07) | 380 (-90; 900) | 6385 | 94% | 6% | 0% | 0% | 0.063 | 0.998 | 1 | 1 |
| NL | 3L value set | (13) | Moderate low back pain | Small | 0.02 (-0.01; 0.05) | -97 (-668; 378) | -4147 | 35% | 58% | 4% | 3% | 0.620 | 0.919 | 0.936 | 0.941 |
|  | 3L to 5L crosswalk |  |  |  | 0.02 (0.001; 0.04) | -97 (-668; 378) | -4490 | 37% | 60% | 1% | 2% | 0.620 | 0.945 | 0.966 | 0.975 |
| US | 3L value set |  |  |  | 0.02 (-0.004; 0.04) | -97 (-668; 378) | -5768 | 35% | 58% | 4% | 3% | 0.620 | 0.898 | 0.925 | 0.939 |
|  | 3L to 5L crosswalk |  |  |  | 0.03 (0.004; 0.05) | -97 (-668; 378) | -3418 | 37% | 61% | 1% | 1% | 0.620 | 0.973 | 0.981 | 0.988 |
| JP | 3L value set |  |  |  | 0.02 (-0.003; 0.04) | -97 (-668; 378) | -5847 | 36% | 58% | 4% | 2% | 0.620 | 0.908 | 0.943 | 0.953 |
|  | 3L to 5L crosswalk |  |  |  | 0.02 (0.004; 0.04) | -97 (-668; 378) | -4926 | 38% | 61% | 1% | 0% | 0.620 | 0.952 | 0.980 | 0.993 |
| NL | 3L value set | (14) | Moderate low back pain | Medium | 0.07 (0.04; 0.10) | 291 (-165; 726) | 4405 | 89% | 11% | 0% | 0% | 0.105 | 0.996 | 0.999 | 1 |
|  | 3L to 5L crosswalk |  |  |  | 0.06 (0.04; 0.08) | 291 (-165; 726) | 4904 | 89% | 11% | 0% | 0% | 0.105 | 0.998 | 1 | 1 |
| US | 3L value set |  |  |  | 0.05 (0.03; 0.07) | 291 (-165; 726) | 5582 | 89% | 11% | 0% | 0% | 0.105 | 0.994 | 0.999 | 1 |
|  | 3L to 5L crosswalk |  |  |  | 0.08 (0.05; 0.10) | 291 (-165; 726) | 3873 | 89% | 11% | 0% | 0% | 0.105 | 1 | 1 | 1 |
| JP | 3L value set |  |  |  | 0.05 (0.03; 0.07) | 291 (-165; 726) | 5769 | 89% | 11% | 0% | 0% | 0.105 | 0.994 | 0.999 | 1 |
|  | 3L to 5L crosswalk |  |  |  | 0.05 (0.03; 0.06) | 291 (-165; 726) | 6009 | 89% | 11% | 0% | 0% | 0.105 | 0.996 | 1 | 1 |
| NL | 3L value set | (15) | Moderate low back pain | Large | 0.11 (0.08; 0.14) | 98 (-327; 559) | 936 | 67% | 33% | 0% | 0% | 0.324 | 1 | 1 | 1 |
|  | 3L to 5L crosswalk |  |  |  | 0.1 (0.09; 0.12) | 98 (-327; 559) | 943 | 67% | 33% | 0% | 0% | 0.324 | 1 | 1 | 1 |
| US | 3L value set |  |  |  | 0.09 (0.07; 0.11) | 98 (-327; 559) | 1111 | 67% | 33% | 0% | 0% | 0.324 | 1 | 1 | 1 |
|  | 3L to 5L crosswalk |  |  |  | 0.12 (0.10; 0.14) | 98 (-327; 559) | 809 | 67% | 33% | 0% | 0% | 0.324 | 1 | 1 | 1 |
| JP | 3L value set |  |  |  | 0.11 (0.09; 0.13) | 98 (-327; 559) | 921 | 67% | 33% | 0% | 0% | 0.324 | 1 | 1 | 1 |
|  | 3L to 5L crosswalk |  |  |  | 0.08 (0.07; 0.10) | 98 (-327; 559) | 1162 | 67% | 33% | 0% | 0% | 0.324 | 1 | 1 | 1 |
| NL | 3L value set | (16) | Severe low back pain | Small | 0.04 (-0.004; 0.09) | 132 (-398; 646) | 3096 | 66% | 31% | 0% | 3% | 0.315 | 0.898 | 0.926 | 0.944 |
|  | 3L to 5L crosswalk |  |  |  | 0.03 (-0.01; 0.06) | 132 (-398; 646) | 5272 | 63% | 30% | 1% | 6% | 0.315 | 0.791 | 0.850 | 0.890 |
| US | 3L value set |  |  |  | 0.03 (-0.004; 0.07) | 132 (-398; 646) | 4183 | 66% | 31% | 0% | 3% | 0.315 | 0.857 | 0.908 | 0.939 |
|  | 3L to 5L crosswalk |  |  |  | 0.03 (-0.003; 0.06) | 132 (-398; 646) | 4390 | 65% | 31% | 0% | 3% | 0.315 | 0.850 | 0.901 | 0.934 |
| JP | 3L value set |  |  |  | 0.04 (0.01; 0.07) | 132 (-398; 646) | 3561 | 68% | 31% | 0% | 1% | 0.315 | 0.928 | 0.969 | 0.984 |
|  | 3L to 5L crosswalk |  |  |  | 0.02 (0.002; 0.04) | 132 (-398; 646) | 6839 | 67% | 31% | 0% | 2% | 0.315 | 0.776 | 0.870 | 0.939 |
| NL | 3L value set | (17) | Severe low back pain | Medium | 0.13 (0.09; 0.18) | -39 (-481; 371) | -297 | 44% | 56% | 0% | 0% | 0.561 | 1 | 1 | 1 |
|  | 3L to 5L crosswalk |  |  |  | 0.10 (0.07; 0.13) | -39 (-481; 371) | -386 | 44% | 56% | 0% | 0% | 0.561 | 1 | 1 | 1 |
| US | 3L value set |  |  |  | 0.08 (0.05; 0.12) | -39 (-481; 371) | -464 | 44% | 56% | 0% | 0% | 0.561 | 1 | 1 | 1 |
|  | 3L to 5L crosswalk |  |  |  | 0.09 (0.06; 0.12) | -39 (-481; 371) | -451 | 44% | 56% | 0% | 0% | 0.561 | 1 | 1 | 1 |
| JP | 3L value set |  |  |  | 0.07 (0.04; 0.10) | -39 (-481; 371) | -562 | 44% | 56% | 0% | 0% | 0.561 | 1 | 1 | 1 |
|  | 3L to 5L crosswalk |  |  |  | 0.05 (0.03; 0.06) | -39 (-481; 371) | -863 | 44% | 56% | 0% | 0% | 0.561 | 1 | 1 | 1 |
| NL | 3L value set | (18) | Severe low back pain | Large | 0.14 (0.11; 0.18) | 113 (-371; 604) | 780 | 67% | 33% | 0% | 0% | 0.324 | 1 | 1 | 1 |
|  | 3L to 5L crosswalk |  |  |  | 0.12 (0.10; 0.15) | 113 (-371; 604) | 936 | 67% | 33% | 0% | 0% | 0.324 | 1 | 1 | 1 |
| US | 3L value set |  |  |  | 0.12 (0.09; 0.14) | 113 (-371; 604) | 976 | 67% | 33% | 0% | 0% | 0.324 | 1 | 1 | 1 |
|  | 3L to 5L crosswalk |  |  |  | 0.13 (0.11; 0.16) | 113 (-371; 604) | 837 | 67% | 33% | 0% | 0% | 0.324 | 1 | 1 | 1 |
| JP | 3L value set |  |  |  | 0.13 (0.10; 0.15) | 113 (-371; 604) | 903 | 67% | 33% | 0% | 0% | 0.324 | 1 | 1 | 1 |
|  | 3L to 5L crosswalk |  |  |  | 0.08 (0.07; 0.10) | 113 (-371; 604) | 1352 | 67% | 33% | 0% | 0% | 0.324 | 1 | 1 | 1 |

**Cont. Supplementary Table 4.1. Cost-utility analysis results for 3L value set and 3L to 5L crosswalk per country**

| **Country** | **Scoring method** | **Scenario** | **Patient population** | **Effect size** | **Incremental QALYs**  **IQ (95% CI)** | **Incremental costs**  **€ (95% CI)** | **ICER**  **€/point** | **Distribution CE-plane (%)** | | | | **Probability of cost-effectiveness** | | | |
| --- | --- | --- | --- | --- | --- | --- | --- | --- | --- | --- | --- | --- | --- | --- | --- |
|  |  |  |  |  |  |  |  | **NE** | **SE** | **SW** | **NW** | **p_CE_(0)** | **p_CE_(20000)** | **p_CE_(30000)** | **p_CE_(50000)** |
| NL | 3L value set | (19) | Mild osteoarthritis | Small | 0.03 (-0.01; 0.07) | 127 (-314; 613) | 3973 | 67% | 29% | 1% | 3% | 0.301 | 0.864 | 0.908 | 0.940 |
|  | 3L to 5L crosswalk |  |  |  | 0.03 (0.002; 0.06) | 127 (-314; 613) | 4506 | 68% | 30% | 0% | 2% | 0.301 | 0.874 | 0.928 | 0.965 |
| US | 3L value set |  |  |  | 0.02 (-0.01; 0.05) | 127 (-314; 613) | 5589 | 65% | 29% | 1% | 5% | 0.301 | **0.798** | **0.853** | 0.893 |
|  | 3L to 5L crosswalk |  |  |  | 0.01 (-0.01; 0.04) | 127 (-314; 613) | 8765 | 58% | 27% | 3% | 2% | 0.301 | **0.662** | **0.726** | 0.785 |
| JP | 3L value set |  |  |  | 0.02 (-0.001; 0.04) | 127 (-314; 613) | 5956 | 68% | 29% | 1% | 2% | 0.301 | **0.800** | **0.880** | 0.932 |
|  | 3L to 5L crosswalk |  |  |  | 0.003 (-0.01; 0.02) | 127 (-314; 613) | 44792 | 43% | 21% | 9% | 27% | 0.301 | **0.426** | **0.726** | 0.785 |
| NL | 3L value set | (20) | Mild osteoarthritis | Medium | 0.04 (0.03; 0.06) | -258 (-788; 193) | -5973 | 16% | 84% | 0% | 0% | 0.845 | 0.999 | 1 | 1 |
|  | 3L to 5L crosswalk |  |  |  | 0.04 (0.02; 0.05) | -258 (-788; 193) | -6848 | 16% | 84% | 0% | 0% | 0.845 | 0.999 | 1 | 1 |
| US | 3L value set |  |  |  | 0.04 (0.02; 0.05) | -258 (-788; 193) | -6948 | 16% | 84% | 0% | 0% | 0.845 | 0.999 | 1 | 1 |
|  | 3L to 5L crosswalk |  |  |  | 0.03 (0.01; 0.05) | -258 (-788; 193) | -9061 | 16% | 84% | 0% | 0% | 0.845 | 0.995 | 0.997 | 0.998 |
| JP | 3L value set |  |  |  | 0.05 (0.03; 0.07) | -258 (-788; 193) | -5126 | 16% | 84% | 0% | 0% | 0.845 | 1 | 1 | 1 |
|  | 3L to 5L crosswalk |  |  |  | 0.02 (0.01; 0.03) | -258 (-788; 193) | -13888 | 16% | 84% | 0% | 0% | 0.845 | 0.984 | 0.992 | 0.996 |
| NL | 3L value set | (21) | Mild osteoarthritis | Large | 0.06 (0.04; 0.07) | 366 (-61; 806) | 6286 | 95% | 5% | 0% | 0% | 0.045 | 0.998 | 1 | 1 |
|  | 3L to 5L crosswalk |  |  |  | 0.06 (0.04; 0.07) | 366 (-61; 806) | 6497 | 95% | 5% | 0% | 0% | 0.045 | 0.999 | 1 | 1 |
| US | 3L value set |  |  |  | 0.06 (0.04; 0.07) | 366 (-61; 806) | 6593 | 95% | 5% | 0% | 0% | 0.045 | 0.997 | 1 | 1 |
|  | 3L to 5L crosswalk |  |  |  | 0.04 (0.03; 0.06) | 366 (-61; 806) | 8754 | 95% | 5% | 0% | 0% | 0.045 | 0.952 | 0.998 | 1 |
| JP | 3L value set |  |  |  | 0.09 (0.08; 0.11) | 366 (-61; 806) | 4001 | 95% | 5% | 0% | 0% | 0.045 | 1 | 1 | 1 |
|  | 3L to 5L crosswalk |  |  |  | 0.04 (0.02; 0.05) | 366 (-61; 806) | 9955 | 95% | 5% | 0% | 0% | 0.045 | 0.919 | 0.966 | 0.999 |
| NL | 3L value set | (22) | Moderate osteoarthritis | Small | 0.02 (-0.01; 0.05) | -97 (-668; 378) | -4147 | 35% | 58% | 4% | 3% | 0.620 | 0.919 | 0.936 | 0.941 |
|  | 3L to 5L crosswalk |  |  |  | 0.02 (0.001; 0.04) | -97 (-668; 378) | -4490 | 37% | 60% | 2% | 1% | 0.620 | 0.945 | 0.966 | 0.975 |
| US | 3L value set |  |  |  | 0.02 (-0.004; 0.04) | -97 (-668; 378) | -5768 | 35% | 58% | 4% | 3% | 0.620 | 0.898 | 0.925 | 0.939 |
|  | 3L to 5L crosswalk |  |  |  | 0.03 (0.004; 0.05) | -97 (-668; 378) | -3418 | 37% | 61% | 1% | 1% | 0.620 | 0.973 | 0.981 | 0.988 |
| JP | 3L value set |  |  |  | 0.02 (-0.003; 0.04) | -97 (-668; 378) | -5847 | 36% | 58% | 4% | 2% | 0.620 | 0.908 | 0.943 | 0.953 |
|  | 3L to 5L crosswalk |  |  |  | 0.02 (0.004; 0.04) | -97 (-668; 378) | -4926 | 38% | 61% | 1% | 0% | 0.620 | 0.952 | 0.980 | 0.993 |
| NL | 3L value set | (23) | Moderate osteoarthritis | Medium | 0.07 (0.04; 0.10) | 291 (-165; 726) | 4405 | 89% | 11% | 0% | 0% | 0.105 | 0.996 | 0.999 | 1 |
|  | 3L to 5L crosswalk |  |  |  | 0.06 (0.04; 0.08) | 291 (-165; 726) | 4904 | 89% | 11% | 0% | 0% | 0.105 | 0.998 | 1 | 1 |
| US | 3L value set |  |  |  | 0.05 (0.03; 0.07) | 291 (-165; 726) | 5581 | 89% | 11% | 0% | 0% | 0.105 | 0.994 | 0.999 | 1 |
|  | 3L to 5L crosswalk |  |  |  | 0.08 (0.05; 0.10) | 291 (-165; 726) | 3873 | 89% | 11% | 0% | 0% | 0.105 | 1 | 1 | 1 |
| JP | 3L value set |  |  |  | 0.05 (0.03; 0.07) | 291 (-165; 726) | 5769 | 89% | 11% | 0% | 0% | 0.105 | 0.994 | 0.999 | 1 |
|  | 3L to 5L crosswalk |  |  |  | 0.05 (0.03; 0.06) | 291 (-165; 726) | 6009 | 89% | 11% | 0% | 0% | 0.105 | 0.996 | 1 | 1 |
| NL | 3L value set | (24) | Moderate osteoarthritis | Large | 0.14 (0.11; 0.17) | 422 (-22; 890) | 3048 | 96% | 4% | 0% | 0% | 0.035 | 1 | 1 | 1 |
|  | 3L to 5L crosswalk |  |  |  | 0.11 (0.09; 0.14) | 422 (-22; 890) | 3741 | 96% | 4% | 0% | 0% | 0.035 | 1 | 1 | 1 |
| US | 3L value set |  |  |  | 0.12 (0.09; 0.14) | 422 (-22; 890) | 3597 | 96% | 4% | 0% | 0% | 0.035 | 1 | 1 | 1 |
|  | 3L to 5L crosswalk |  |  |  | 0.13 (0.11; 0.16) | 422 (-22; 890) | 3141 | 96% | 4% | 0% | 0% | 0.035 | 1 | 1 | 1 |
| JP | 3L value set |  |  |  | 0.10 (0.08; 0.12) | 422 (-22; 890) | 4337 | 96% | 4% | 0% | 0% | 0.035 | 1 | 1 | 1 |
|  | 3L to 5L crosswalk |  |  |  | 0.09 (0.07; 0.10) | 422 (-22; 890) | 4945 | 96% | 4% | 0% | 0% | 0.035 | 1 | 1 | 1 |
| NL | 3L value set | (25) | Severe osteoarthritis | Small | 0.04 (-0.004; 0.09) | 132 (-399; 646) | 3097 | 66% | 31% | 0% | 3% | 0.315 | 0.898 | 0.926 | 0.945 |
|  | 3L to 5L crosswalk |  |  |  | 0.03 (-0.01; 0.06) | 132 (-399; 646) | 5272 | 63% | 30% | 1% | 6% | 0.315 | 0.791 | 0.850 | 0.890 |
| US | 3L value set |  |  |  | 0.03 (-0.004; 0.07) | 132 (-399; 646) | 4183 | 66% | 31% | 0% | 3% | 0.315 | 0.858 | 0.908 | 0.939 |
|  | 3L to 5L crosswalk |  |  |  | 0.03 (-0.003; 0.06) | 132 (-399; 646) | 4389 | 65% | 32% | 0% | 3% | 0.315 | 0.850 | 0.901 | 0.934 |
| JP | 3L value set |  |  |  | 0.04 (0.01; 0.07) | 132 (-399; 646) | 3561 | 68% | 31% | 0% | 1% | 0.315 | 0.928 | 0.969 | 0.984 |
|  | 3L to 5L crosswalk |  |  |  | 0.02 (0.002; 0.04) | 132 (-399; 646) | 6839 | 67% | 31% | 0% | 2% | 0.315 | 0.776 | 0.870 | 0.939 |
| NL | 3L value set | (26) | Severe osteoarthritis | Medium | 0.07 (0.04; 0.09) | -430 (-890; -37) | -6329 | 2% | 98% | 0% | 0% | 0.982 | 1 | 1 | 1 |
|  | 3L to 5L crosswalk |  |  |  | 0.02 (-0.002; 0.03) | -430 (-890; -37) | -26515 | 1% | 94% | 5% | 0% | 0.982 | 0.994 | 0.993 | 0.988 |
| US | 3L value set |  |  |  | 0.05 (0.03; 0.07) | -430 (-890; -37) | -8861 | 2% | 98% | 0% | 0% | 0.982 | 1 | 1 | 1 |
|  | 3L to 5L crosswalk |  |  |  | 0.02 (0.01; 0.04) | -430 (-890; -37) | -17214 | 2% | 98% | 0% | 0% | 0.982 | 0.999 | 0.999 | 0.999 |
| JP | 3L value set |  |  |  | 0.05 (0.03; 0.07) | -430 (-890; -37) | -9060 | 2% | 98% | 0% | 0% | 0.982 | 1 | 1 | 1 |
|  | 3L to 5L crosswalk |  |  |  | 0.01 (0.005; 0.02) | -430 (-890; -37) | -31030 | 2% | 98% | 0% | 0% | 0.982 | 0.998 | 0.998 | 0.999 |
| NL | 3L value set | (27) | Severe osteoarthritis | Large | 0.13 (0.10; 0.17) | 357 (-95; 796) | 2650 | 95% | 5% | 0% | 0% | 0.050 | 1 | 1 | 1 |
|  | 3L to 5L crosswalk |  |  |  | 0.09 (0.06; 0.11) | 357 (-95; 796) | 4084 | 95% | 5% | 0% | 0% | 0.050 | 1 | 1 | 1 |
| US | 3L value set |  |  |  | 0.10 (0.08; 0.13) | 357 (-95; 796) | 3526 | 95% | 5% | 0% | 0% | 0.050 | 1 | 1 | 1 |
|  | 3L to 5L crosswalk |  |  |  | 0.10 (0.08; 0.12) | 357 (-95; 796) | 3595 | 95% | 5% | 0% | 0% | 0.050 | 1 | 1 | 1 |
| JP | 3L value set |  |  |  | 0.09 (0.06; 0.11) | 357 (-95; 796) | 4141 | 95% | 5% | 0% | 0% | 0.050 | 1 | 1 | 1 |
|  | 3L to 5L crosswalk |  |  |  | 0.05 (0.04; 0.06) | 357 (-95; 796) | 7222 | 95% | 5% | 0% | 0% | 0.050 | 0.989 | 1 | 1 |

**Cont. Supplementary Table 4.1. Cost-utility analysis results for 3L value set and 3L to 5L crosswalk per country**

| **Country** | **Scoring method** | **Scenario** | **Patient population** | **Effect size** | **Incremental QALYs**  **IQ (95% CI)** | **Incremental costs**  **€ (95% CI)** | **ICER**  **€/point** | **Distribution CE-plane (%)** | | | | **Probability of cost-effectiveness** | | | |
| --- | --- | --- | --- | --- | --- | --- | --- | --- | --- | --- | --- | --- | --- | --- | --- |
|  |  |  |  |  |  |  |  | **NE** | **SE** | **SW** | **NW** | **p_CE_(0)** | **p_CE_(20000)** | **p_CE_(30000)** | **p_CE_(50000)** |
| NL | 3L value set | (28) | Mild cancer | Small | 0.03 (-0.003; 0.05) | 178 (-293; 677) | 6517 | 74% | 23% | 10% | 3% | 0.236 | **0.826** | 0.889 | 0.930 |
|  | 3L to 5L crosswalk |  |  |  | 0.02 (-0.01; 0.04) | 178 (-293; 677) | 11279 | 71% | 22% | 2% | 5% | 0.236 | **0.659** | 0.759 | 0.845 |
| US | 3L value set |  |  |  | 0.03 (0.01; 0.05) | 178 (-293; 677) | 5804 | 76% | 23% | 0% | 1% | 0.236 | 0.882 | 0.944 | 0.969 |
|  | 3L to 5L crosswalk |  |  |  | 0.02 (0.003; 0.05) | 178 (-293; 677) | 7165 | 75% | 23% | 0% | 1% | 0.236 | 0.819 | 0.903 | 0.952 |
| JP | 3L value set |  |  |  | 0.02 (-0.0004; 0.04) | 178 (-293; 677) | 9007 | 74% | 23% | 1% | 2% | 0.236 | 0.743 | **0.842** | 0.916 |
|  | 3L to 5L crosswalk |  |  |  | 0.01 (-0.003; 0.03) | 178 (-293; 677) | 14832 | 71% | 22% | 2% | 5% | 0.236 | 0.573 | **0.699** | 0.816 |
| NL | 3L value set | (29) | Mild cancer | Medium | 0.03 (0.02; 0.04) | 41 (-401; 492) | 1408 | 56% | 44% | 0% | 0% | 0.436 | 0.979 | 0.995 | 0.999 |
|  | 3L to 5L crosswalk |  |  |  | 0.03 (0.01; 0.04) | 41 (-401; 492) | 1540 | 56% | 44% | 0% | 0% | 0.436 | 0.973 | 0.992 | 0.999 |
| US | 3L value set |  |  |  | 0.03 (0.02; 0.05) | 41 (-401; 492) | 1236 | 56% | 44% | 0% | 0% | 0.436 | 0.990 | 0.998 | 1 |
|  | 3L to 5L crosswalk |  |  |  | 0.02 (0.01; 0.04) | 41 (-401; 492) | 1681 | 56% | 44% | 0% | 0% | 0.436 | 0.963 | 0.990 | 0.999 |
| JP | 3L value set |  |  |  | 0.05 (0.03; 0.07) | 41 (-401; 492) | 821 | 56% | 44% | 0% | 0% | 0.436 | 0.999 | 1 | 1 |
|  | 3L to 5L crosswalk |  |  |  | 0.02 (0.01; 0.03) | 41 (-401; 492) | 1906 | 56% | 44% | 0% | 0% | 0.436 | 0.944 | 0.985 | 0.997 |
| NL | 3L value set | (30) | Mild cancer | Large | 0.05 (0.04; 0.06) | 53 (-396; 502) | 1100 | 58% | 42% | 0% | 0% | 0.421 | 1 | 1 | 1 |
|  | 3L to 5L crosswalk |  |  |  | 0.05 (0.04; 0.06) | 53 (-396; 502) | 1124 | 58% | 42% | 0% | 0% | 0.421 | 1 | 1 | 1 |
| US | 3L value set |  |  |  | 0.06 (0.04; 0.07) | 53 (-396; 502) | 964 | 58% | 42% | 0% | 0% | 0.421 | 1 | 1 | 1 |
|  | 3L to 5L crosswalk |  |  |  | 0.05 (0.04; 0.06) | 53 (-396; 502) | 1026 | 58% | 42% | 0% | 0% | 0.421 | 1 | 1 | 1 |
| JP | 3L value set |  |  |  | 0.09 (0.07; 0.10) | 53 (-396; 502) | 618 | 58% | 42% | 0% | 0% | 0.421 | 1 | 1 | 1 |
|  | 3L to 5L crosswalk |  |  |  | 0.05 (0.04; 0.06) | 53 (-396; 502) | 1156 | 58% | 42% | 0% | 0% | 0.421 | 1 | 1 | 1 |
| NL | 3L value set | (31) | Moderate cancer | Small | 0.03 (-0.004; 0.07) | 285 (-121; 712) | 8717 | 86% | 10% | 0% | 4% | 0.101 | 0.801 | 0.872 | 0.909 |
|  | 3L to 5L crosswalk |  |  |  | 0.05 (0.02; 0.08) | 285 (-121; 712) | 6083 | 90% | 10% | 0% | 0% | 0.101 | 0.952 | 0.984 | 0.995 |
| US | 3L value set |  |  |  | 0.03 (-0.003; 0.06) | 285 (-121; 712) | 10775 | 86% | 10% | 0% | 4% | 0.101 | **0.745** | 0.845 | 0.908 |
|  | 3L to 5L crosswalk |  |  |  | 0.06 (0.03; 0.09) | 285 (-121; 712) | 4768 | 90% | 10% | 0% | 0% | 0.101 | **0.989** | 0.997 | 1 |
| JP | 3L value set |  |  |  | 0.03 (0.002; 0.05) | 285 (-121; 712) | 11054 | 89% | 10% | 0% | 1% | 0.101 | **0.756** | 0.868 | 0.934 |
|  | 3L to 5L crosswalk |  |  |  | 0.04 (0.02; 0.06) | 285 (-121; 712) | 6609 | 90% | 10% | 0% | 0% | 0.101 | **0.978** | 0.998 | 1 |
| NL | 3L value set | (32) | Moderate cancer | Medium | 0.07 (0.04; 0.10) | -174 (-628; 281) | -2534 | 22% | 78% | 0% | 0% | 0.776 | 1 | 1 | 1 |
|  | 3L to 5L crosswalk |  |  |  | 0.08 (0.06; 0.11) | -174 (-628; 281) | -2053 | 22% | 78% | 0% | 0% | 0.776 | 1 | 1 | 1 |
| US | 3L value set |  |  |  | 0.06 (0.03; 0.08) | -174 (-628; 281) | -3111 | 22% | 78% | 0% | 0% | 0.776 | 0.999 | 1 | 1 |
|  | 3L to 5L crosswalk |  |  |  | 0.09 (0.06; 0.11) | -174 (-628; 281) | -1978 | 22% | 78% | 0% | 0% | 0.776 | 1 | 1 | 1 |
| JP | 3L value set |  |  |  | 0.08 (0.06; 0.11) | -174 (-628; 281) | -2062 | 22% | 78% | 0% | 0% | 0.776 | 1 | 1 | 1 |
|  | 3L to 5L crosswalk |  |  |  | 0.07 (0.05; 0.09) | -174 (-628; 281) | -2539 | 22% | 78% | 0% | 0% | 0.776 | 1 | 1 | 1 |
| NL | 3L value set | (33) | Moderate cancer | Large | 0.10 (0.08; 0.13) | 94 (-329; 514) | 940 | 68% | 32% | 0% | 0% | 0.314 | 1 | 1 | 1 |
|  | 3L to 5L crosswalk |  |  |  | 0.11 (0.09; 0.13) | 94 (-329; 514) | 885 | 68% | 32% | 0% | 0% | 0.314 | 1 | 1 | 1 |
| US | 3L value set |  |  |  | 0.09 (0.07; 0.11) | 94 (-329; 514) | 1035 | 68% | 32% | 0% | 0% | 0.314 | 1 | 1 | 1 |
|  | 3L to 5L crosswalk |  |  |  | 0.11 (0.09; 0.14) | 94 (-329; 514) | 830 | 68% | 32% | 0% | 0% | 0.314 | 1 | 1 | 1 |
| JP | 3L value set |  |  |  | 0.12 (0.10; 0.14) | 94 (-329; 514) | 797 | 68% | 32% | 0% | 0% | 0.314 | 1 | 1 | 1 |
|  | 3L to 5L crosswalk |  |  |  | 0.09 (0.08; 0.11) | 94 (-329; 514) | 1036 | 68% | 32% | 0% | 0% | 0.314 | 1 | 1 | 1 |
| NL | 3L value set | (34) | Severe cancer | Small | 0.05 (-0.002; 0.11) | 364 (-119; 965) | 6737 | 89% | 9% | 0% | 2% | 0.085 | 0.872 | 0.915 | 0.945 |
|  | 3L to 5L crosswalk |  |  |  | 0.05 (0.01; 0.10) | 364 (-119; 965) | 6868 | 90% | 9% | 0% | 1% | 0.085 | 0.891 | 0.942 | 0.967 |
| US | 3L value set |  |  |  | 0.04 (-0.003; 0.09) | 364 (-119; 965) | 8414 | 88% | 9% | 0% | 3% | 0.085 | 0.826 | 0.881 | 0.924 |
|  | 3L to 5L crosswalk |  |  |  | 0.06 (0.01; 0.11) | 364 (-119; 965) | 5948 | 91% | 8% | 0% | 1% | 0.085 | 0.920 | 0.957 | 0.975 |
| JP | 3L value set |  |  |  | 0.04 (-0.003; 0.08) | 364 (-119; 965) | 9082 | 88% | 8% | 0% | 4% | 0.085 | 0.800 | 0.870 | 0.916 |
|  | 3L to 5L crosswalk |  |  |  | 0.04 (0.01; 0.07) | 364 (-119; 965) | 9782 | 91% | 8% | 0% | 1% | 0.085 | 0.818 | 0.903 | 0.959 |
| NL | 3L value set | (35) | Severe cancer | Medium | 0.14 (0.09; 0.20) | 109 (-409; 617) | 775 | 66% | 34% | 0% | 0% | 0.336 | 1 | 1 | 1 |
|  | 3L to 5L crosswalk |  |  |  | 0.11 (0.07; 0.16) | 109 (-409; 617) | 958 | 66% | 34% | 0% | 0% | 0.336 | 1 | 1 | 1 |
| US | 3L value set |  |  |  | 0.11 (0.06; 0.16) | 109 (-409; 617) | 998 | 66% | 34% | 0% | 0% | 0.336 | 1 | 1 | 1 |
|  | 3L to 5L crosswalk |  |  |  | 0.13 (0.07; 0.18) | 109 (-409; 617) | 871 | 66% | 34% | 0% | 0% | 0.336 | 1 | 1 | 1 |
| JP | 3L value set |  |  |  | 0.10 (0.05; 0.14) | 109 (-409; 617) | 1143 | 66% | 34% | 0% | 0% | 0.336 | 1 | 1 | 1 |
|  | 3L to 5L crosswalk |  |  |  | 0.08 (0.05; 0.11) | 109 (-409; 617) | 1391 | 66% | 34% | 0% | 0% | 0.336 | 1 | 1 | 1 |
| NL | 3L value set | (36) | Severe cancer | Large | 0.24 (0.18; 0.29) | -217 (-690; 238) | -921 | 18% | 82% | 0% | 0% | 0.825 | 1 | 1 | 1 |
|  | 3L to 5L crosswalk |  |  |  | 0.18 (0.14; 0.23) | -217 (-690; 238) | -1180 | 18% | 82% | 0% | 0% | 0.825 | 1 | 1 | 1 |
| US | 3L value set |  |  |  | 0.19 (0.15; 0.24) | -217 (-690; 238) | -1134 | 18% | 82% | 0% | 0% | 0.825 | 1 | 1 | 1 |
|  | 3L to 5L crosswalk |  |  |  | 0.20 (0.15; 0.25) | -217 (-690; 238) | -1108 | 18% | 82% | 0% | 0% | 0.825 | 1 | 1 | 1 |
| JP | 3L value set |  |  |  | 0.16 (0.12; 0.21) | -217 (-690; 238) | -1323 | 18% | 82% | 0% | 0% | 0.825 | 1 | 1 | 1 |
|  | 3L to 5L crosswalk |  |  |  | 0.12 (0.09; 0.15) | -217 (-690; 238) | -1775 | 18% | 82% | 0% | 0% | 0.825 | 1 | 1 | 1 |

NL: the Netherlands. US: United States. JP: Japan. QALY: quality-adjusted life-year. IQ: incremental QALY. 95% CI: 95% confidence interval. ICER: Incremental Cost-Effectiveness Ratio. NE: northeast. SE: southeast. SW: southwest. NW: northwest. p_CE_: probability of the intervention being cost-effective compared to control.

**Supplementary Table 4.2. Cost-utility analysis results for 5L value set and 5L to 3L crosswalk per country**

| **Country** | **Scoring method** | **Scenario** | **Patient population** | **Effect size** | **Incremental QALYs**  **IQ (95% CI)** | **Incremental costs**  **€ (95% CI)** | **ICER**  **€/point** | **Distribution CE-plane (%)** | | | | **Probability of cost-effectiveness** | | | |
| --- | --- | --- | --- | --- | --- | --- | --- | --- | --- | --- | --- | --- | --- | --- | --- |
|  |  |  |  |  |  |  |  | **NE** | **SE** | **SW** | **NW** | **p_CE_(0)** | **p_CE_(20000)** | **p_CE_(30000)** | **p_CE_(50000)** |
| NL | 5L value set | (1) | Mild depression | Small | 0.02 (-0.02; 0.06) | 195 (-320; 742) | 8552 | 64% | 22% | 2% | 12% | 0.242 | 0.696 | 0.758 | 0.801 |
|  | 5L to 3L crosswalk |  |  |  | 0.02 (-0.01; 0.06) | 195 (-320; 742) | 8937 | 65% | 22% | 2% | 11% | 0.242 | 0.699 | 0.757 | 0.808 |
| US | 5L value set |  |  |  | 0.03 (-0.01; 0.08) | 195 (-320; 742) | 6221 | 69% | 23% | 1% | 7% | 0.242 | **0.790** | 0.833 | 0.868 |
|  | 5L to 3L crosswalk |  |  |  | 0.02 (-0.01; 0.05) | 195 (-320; 742) | 8447 | 70% | 23% | 1% | 6% | 0.242 | **0.743** | 0.817 | 0.867 |
| JP | 5L value set |  |  |  | 0.01 (-0.03; 0.05) | 195 (-320; 742) | 15705 | 54% | 19% | 5% | 22% | 0.242 | **0.537** | **0.600** | **0.658** |
|  | 5L to 3L crosswalk |  |  |  | 0.02 (-0.001; 0.05) | 195 (-320; 742) | 8107 | 73% | 23% | 1% | 3% | 0.242 | **0.782** | **0.861** | **0.821** |
| NL | 5L value set | (2) | Mild depression | Medium | 0.11 (0.08; 0.15) | -228 (-692; 295) | -2049 | 19% | 81% | 0% | 0% | 0.809 | 1 | 1 | 1 |
|  | 5L to 3L crosswalk |  |  |  | 0.12 (0.09; 0.15) | -228 (-692; 295) | -1902 | 19% | 81% | 0% | 0% | 0.809 | 1 | 1 | 1 |
| US | 5L value set |  |  |  | 0.13 (0.09; 0.17) | -228 (-692; 295) | -1800 | 19% | 81% | 0% | 0% | 0.809 | 1 | 1 | 1 |
|  | 5L to 3L crosswalk |  |  |  | 0.11 (0.08; 0.13) | -228 (-692; 295) | -2151 | 19% | 81% | 0% | 0% | 0.809 | 1 | 1 | 1 |
| JP | 5L value set |  |  |  | 0.08 (0.05; 0.12) | -228 (-692; 295) | -2858 | 19% | 81% | 0% | 0% | 0.809 | 1 | 1 | 1 |
|  | 5L to 3L crosswalk |  |  |  | 0.09 (0.07; 0.11) | -228 (-692; 295) | -2470 | 19% | 81% | 0% | 0% | 0.809 | 1 | 1 | 1 |
| NL | 5L value set | (3) | Mild depression | Large | 0.18 (0.14; 0.21) | 274 (-217; 724) | 1557 | 89% | 11% | 0% | 0% | 0.113 | 1 | 1 | 1 |
|  | 5L to 3L crosswalk |  |  |  | 0.19 (0.16; 0.22) | 274 (-217; 724) | 1440 | 89% | 11% | 0% | 0% | 0.113 | 1 | 1 | 1 |
| US | 5L value set |  |  |  | 0.18 (0.14; 0.22) | 274 (-217; 724) | 1511 | 89% | 11% | 0% | 0% | 0.113 | 1 | 1 | 1 |
|  | 5L to 3L crosswalk |  |  |  | 0.17 (0.14; 0.19) | 274 (-217; 724) | 1627 | 89% | 11% | 0% | 0% | 0.113 | 1 | 1 | 1 |
| JP | 5L value set |  |  |  | 0.14 (0.1; 0.17) | 274 (-217; 724) | 2018 | 89% | 11% | 0% | 0% | 0.113 | 1 | 1 | 1 |
|  | 5L to 3L crosswalk |  |  |  | 0.16 (0.14; 0.18) | 274 (-217; 724) | 1687 | 89% | 11% | 0% | 0% | 0.113 | 1 | 1 | 1 |
| NL | 5L value set | (4) | Moderate depression | Small | 0.05 (0.003; 0.08) | 43 (-448; 524) | 952 | 55% | 43% | 1% | 1% | 0.439 | 0.952 | 0.968 | 0.975 |
|  | 5L to 3L crosswalk |  |  |  | 0.07 (0.03; 0.10) | 43 (-448; 524) | 628 | 56% | 44% | 0% | 0% | 0.439 | 0.995 | 0.999 | 0.999 |
| US | 5L value set |  |  |  | 0.05 (0.003; 0.09) | 43 (-448; 524) | 898 | 55% | 43% | 1% | 1% | 0.439 | 0.960 | 0.972 | 0.979 |
|  | 5L to 3L crosswalk |  |  |  | 0.06 (0.03; 0.09) | 43 (-448; 524) | 713 | 56% | 44% | 0% | 0% | 0.439 | 0.997 | 1 | 1 |
| JP | 5L value set |  |  |  | 0.02 (-0.02; 0.06) | 43 (-448; 524) | 2236 | 47% | 38% | 6% | 9% | 0.439 | 0.772 | 0.806 | 0.827 |
|  | 5L to 3L crosswalk |  |  |  | 0.04 (0.02; 0.06) | 43 (-448; 524) | 1093 | 56% | 44% | 0% | 0% | 0.439 | 0.986 | 0.994 | 0.999 |
| NL | 5L value set | (5) | Moderate depression | Medium | 0.13 (0.09; 0.16) | -296 (-813; 193) | -2350 | 13% | 87% | 0% | 0% | 0.873 | 1 | 1 | 1 |
|  | 5L to 3L crosswalk |  |  |  | 0.14 (0.10; 0.17) | -296 (-813; 193) | -2141 | 13% | 87% | 0% | 0% | 0.873 | 1 | 1 | 1 |
| US | 5L value set |  |  |  | 0.13 (0.09; 0.17) | -296 (-813; 193) | -2248 | 13% | 87% | 0% | 0% | 0.873 | 1 | 1 | 1 |
|  | 5L to 3L crosswalk |  |  |  | 0.12 (0.09; 0.14) | -296 (-813; 193) | -2516 | 13% | 87% | 0% | 0% | 0.873 | 1 | 1 | 1 |
| JP | 5L value set |  |  |  | 0.08 (0.05; 0.12) | -296 (-813; 193) | -3526 | 13% | 87% | 0% | 0% | 0.873 | 1 | 1 | 1 |
|  | 5L to 3L crosswalk |  |  |  | 0.10 (0.08; 0.12) | -296 (-813; 193) | -2896 | 13% | 87% | 0% | 0% | 0.873 | 1 | 1 | 1 |
| NL | 5L value set | (6) | Moderate depression | Large | 0.19 (0.15; 0.22) | -110 (-493; 312) | -590 | 29% | 71% | 0% | 0% | 0.709 | 1 | 1 | 1 |
|  | 5L to 3L crosswalk |  |  |  | 0.20 (0.17; 0.23) | -110 (-493; 312) | -551 | 29% | 71% | 0% | 0% | 0.709 | 1 | 1 | 1 |
| US | 5L value set |  |  |  | 0.20 (0.16; 0.24) | -110 (-493; 312) | -559 | 29% | 71% | 0% | 0% | 0.709 | 1 | 1 | 1 |
|  | 5L to 3L crosswalk |  |  |  | 0.18 (0.15; 0.2) | -110 (-493; 312) | -622 | 29% | 71% | 0% | 0% | 0.709 | 1 | 1 | 1 |
| JP | 5L value set |  |  |  | 0.14 (0.1; 0.18) | -110 (-493; 312) | -770 | 29% | 71% | 0% | 0% | 0.709 | 1 | 1 | 1 |
|  | 5L to 3L crosswalk |  |  |  | 0.17 (0.15; 0.18) | -110 (-493; 312) | -664 | 29% | 71% | 0% | 0% | 0.709 | 1 | 1 | 1 |
| NL | 5L value set | (7) | Severe depression | Small | 0.04 (-0.01; 0.09) | 68 (-448; 571) | 1810 | 56% | 37% | 2% | 5% | 0.391 | 0.867 | 0.893 | 0.912 |
|  | 5L to 3L crosswalk |  |  |  | 0.03 (-0.01; 0.07) | 68 (-448; 571) | 2391 | 55% | 36% | 3% | 6% | 0.391 | 0.830 | 0.862 | 0.886 |
| US | 5L value set |  |  |  | 0.05 (-0.003; 0.10) | 68 (-448; 571) | 1373 | 58% | 38% | 1% | 3% | 0.391 | 0.927 | 0.941 | 0.952 |
|  | 5L to 3L crosswalk |  |  |  | 0.03 (-0.01; 0.06) | 68 (-448; 571) | 2618 | 57% | 37% | 2% | 4% | 0.391 | 0.848 | 0.886 | 0.914 |
| JP | 5L value set |  |  |  | 0.04 (-0.004; 0.09) | 68 (-448; 571) | 1523 | 58% | 38% | 1% | 3% | 0.391 | 0.922 | 0.937 | 0.949 |
|  | 5L to 3L crosswalk |  |  |  | 0.02 (-0.003; 0.05) | 68 (-448; 571) | 2883 | 58% | 38% | 1% | 3% | 0.391 | 0.848 | 0.901 | 0.932 |
| NL | 5L value set | (8) | Severe depression | Medium | 0.12 (0.07; 0.17) | -378 (-919; 140) | -3130 | 7% | 93% | 0% | 0% | .933 | 1 | 1 | 1 |
|  | 5L to 3L crosswalk |  |  |  | 0.10 (0.06; 0.14) | -378 (-919; 140) | -3619 | 7% | 93% | 0% | 0% | .933 | 1 | 1 | 1 |
| US | 5L value set |  |  |  | 0.14 (0.09; 0.19) | -378 (-919; 140) | -2726 | 7% | 93% | 0% | 0% | .933 | 1 | 1 | 1 |
|  | 5L to 3L crosswalk |  |  |  | 0.09 (0.06; 0.12) | -378 (-919; 140) | -4111 | 7% | 93% | 0% | 0% | .933 | 1 | 1 | 1 |
| JP | 5L value set |  |  |  | 0.11 (0.06; 0.15) | -378 (-919; 140) | -3546 | 7% | 93% | 0% | 0% | .933 | 1 | 1 | 1 |
|  | 5L to 3L crosswalk |  |  |  | 0.08 (0.05; 0.1) | -378 (-919; 140) | -4739 | 7% | 93% | 0% | 0% | .933 | 1 | 1 | 1 |
| NL | 5L value set | (9) | Severe depression | Large | 0.21 (0.16; 0.25) | 83 (-405; 581) | 406 | 65% | 35% | 0% | 0% | 0.354 | 1 | 1 | 1 |
|  | 5L to 3L crosswalk |  |  |  | 0.20 (0.17; 0.24) | 83 (-405; 581) | 413 | 65% | 35% | 0% | 0% | 0.354 | 1 | 1 | 1 |
| US | 5L value set |  |  |  | 0.21 (0.16; 0.26) | 83 (-405; 581) | 391 | 65% | 35% | 0% | 0% | 0.354 | 1 | 1 | 1 |
|  | 5L to 3L crosswalk |  |  |  | 0.18 (0.15; 0.2) | 83 (-405; 581) | 477 | 65% | 35% | 0% | 0% | 0.354 | 1 | 1 | 1 |
| JP | 5L value set |  |  |  | 0.18 (0.13; 0.22) | 83 (-405; 581) | 472 | 65% | 35% | 0% | 0% | 0.354 | 1 | 1 | 1 |
|  | 5L to 3L crosswalk |  |  |  | 0.17 (0.14; 0.19) | 83 (-405; 581) | 500 | 65% | 35% | 0% | 0% | 0.354 | 1 | 1 | 1 |

**Cont. Supplementary Table 4.2. Cost-utility analysis results for 5L value set and 5L to 3L crosswalk per country**

| **Country** | **Scoring method** | **Scenario** | **Patient population** | **Effect size** | **Incremental QALYs**  **IQ (95% CI)** | **Incremental costs**  **€ (95% CI)** | **ICER**  **€/point** | **Distribution CE-plane (%)** | | | | **Probability of cost-effectiveness** | | | |
| --- | --- | --- | --- | --- | --- | --- | --- | --- | --- | --- | --- | --- | --- | --- | --- |
|  |  |  |  |  |  |  |  | **NE** | **SE** | **SW** | **NW** | **p_CE_(0)** | **p_CE_(20000)** | **p_CE_(30000)** | **p_CE_(50000)** |
| NL | 5L value set | (10) | Mild low back pain | Small | 0.03 (-0.01; 0.78) | 460 (-6; 1041) | 13849 | 90% | 3% | 0% | 7% | 0.034 | 0.641 | 0.764 | 0.845 |
|  | 5L to 3L crosswalk |  |  |  | 0.03 (-0.002; 0.07) | 460 (-6; 1041) | 13646 | 93% | 3% | 1% | 3% | 0.034 | 0.678 | 0.814 | 0.895 |
| US | 5L value set |  |  |  | 0.05 (0.01; 0.10) | 460 (-6; 1041) | 9019 | 95% | 3% | 0% | 2% | 0.034 | 0.835 | 0.912 | 0.954 |
|  | 5L to 3L crosswalk |  |  |  | 0.04 (0.01; 0.07) | 460 (-6; 1041) | 12013 | 96% | 3% | 0% | 1% | 0.034 | 0.773 | 0.902 | 0.973 |
| JP | 5L value set |  |  |  | 0.03 (-0.01; 0.07) | 460 (-6; 1041) | 17604 | 85% | 3% | 1% | 11% | 0.034 | **0.549** | **0.668** | 0.775 |
|  | 5L to 3L crosswalk |  |  |  | 0.04 (0.02; 0.06) | 460 (-6; 1041) | 11345 | 96% | 4% | 0% | 0% | 0.034 | **0.841** | **0.958** | 0.996 |
| NL | 5L value set | (11) | Mild low back pain | Medium | 0.11 (0.06; 0.15) | 393 (-134; 953) | 3703 | 92% | 8% | 0% | 0% | 0.075 | 0.998 | 0.999 | 1 |
|  | 5L to 3L crosswalk |  |  |  | 0.12 (0.09; 0.15) | 393 (-134; 953) | 3277 | 92% | 8% | 0% | 0% | 0.075 | 1 | 1 | 1 |
| US | 5L value set |  |  |  | 0.13 (0.09; 0.18) | 393 (-134; 953) | 2944 | 92% | 8% | 0% | 0% | 0.075 | 1 | 1 | 1 |
|  | 5L to 3L crosswalk |  |  |  | 0.12 (0.09; 0.14) | 393 (-134; 953) | 3377 | 92% | 8% | 0% | 0% | 0.075 | 1 | 1 | 1 |
| JP | 5L value set |  |  |  | 0.10 (0.06; 0.14) | 393 (-134; 953) | 4138 | 92% | 8% | 0% | 0% | 0.075 | 0.997 | 1 | 1 |
|  | 5L to 3L crosswalk |  |  |  | 0.12 (0.10; 0.14) | 393 (-134; 953) | 3364 | 92% | 8% | 0% | 0% | 0.075 | 0.997 | 1 | 1 |
| NL | 5L value set | (12) | Mild low back pain | Large | 0.21 (0.17; 0.26) | 225 (-221; 756) | 1057 | 82% | 18% | 0% | 0% | 0.174 | 1 | 1 | 1 |
|  | 5L to 3L crosswalk |  |  |  | 0.25 (0.21; 0.28) | 225 (-221; 756) | 914 | 82% | 18% | 0% | 0% | 0.174 | 1 | 1 | 1 |
| US | 5L value set |  |  |  | 0.24 (0.20; 0.29) | 225 (-221; 756) | 933 | 82% | 18% | 0% | 0% | 0.174 | 1 | 1 | 1 |
|  | 5L to 3L crosswalk |  |  |  | 0.24 (0.21; 0.26) | 225 (-221; 756) | 951 | 82% | 18% | 0% | 0% | 0.174 | 1 | 1 | 1 |
| JP | 5L value set |  |  |  | 0.18 (0.14; 0.22) | 225 (-221; 756) | 1265 | 82% | 18% | 0% | 0% | 0.174 | 1 | 1 | 1 |
|  | 5L to 3L crosswalk |  |  |  | 0.22 (0.20; 0.24) | 225 (-221; 756) | 1039 | 82% | 18% | 0% | 0% | 0.174 | 1 | 1 | 1 |
| NL | 5L value set | (13) | Moderate low back pain | Small | 0.02 (-0.02; 0.06) | 233 (-186; 703) | 11662 | 70% | 13% | 2% | 15% | 0.152 | **0.642** | **0.711** | 0.764 |
|  | 5L to 3L crosswalk |  |  |  | 0.04 (0.01; 0.08) | 233 (-186; 703) | 5250 | 84% | 15% | 0% | 1% | 0.152 | **0.939** | **0.962** | 0.984 |
| US | 5L value set |  |  |  | 0.04 (-0.01; 0.08) | 233 (-186; 703) | 5845 | 81% | 15% | 1% | 3% | 0.152 | 0.864 | 0.910 | 0.935 |
|  | 5L to 3L crosswalk |  |  |  | 0.04 (0.01; 0.06) | 233 (-186; 703) | 5707 | 85% | 15% | 0% | 0% | 0.152 | 0.949 | 0.979 | 0.994 |
| JP | 5L value set |  |  |  | 0.02 (-0.02; 0.06) | 233 (-186; 703) | 11900 | 70% | 13% | 2% | 15% | 0.152 | **0.630** | **0.705** | 0.762 |
|  | 5L to 3L crosswalk |  |  |  | 0.04 (0.02; 0.05) | 233 (-186; 703) | 6540 | 85% | 15% | 0% | 0% | 0.152 | **0.939** | **0.982** | 0.998 |
| NL | 5L value set | (14) | Moderate low back pain | Medium | 0.12 (0.07; 0.16) | 39 (-416; 488) | 330 | 56% | 44% | 0% | 0% | 0.435 | 1 | 1 | 1 |
|  | 5L to 3L crosswalk |  |  |  | 0.14 (0.10; 0.17) | 39 (-416; 488) | 281 | 56% | 44% | 0% | 0% | 0.435 | 1 | 1 | 1 |
| US | 5L value set |  |  |  | 0.15 (0.10; 0.20) | 39 (-416; 488) | 258 | 56% | 44% | 0% | 0% | 0.435 | 1 | 1 | 1 |
|  | 5L to 3L crosswalk |  |  |  | 0.13 (0.10; 0.15) | 39 (-416; 488) | 308 | 56% | 44% | 0% | 0% | 0.435 | 1 | 1 | 1 |
| JP | 5L value set |  |  |  | 0.09 (0.05; 0.13) | 39 (-416; 488) | 418 | 56% | 44% | 0% | 0% | 0.435 | 1 | 1 | 1 |
|  | 5L to 3L crosswalk |  |  |  | 0.11 (0.09; 0.13) | 39 (-416; 488) | 356 | 56% | 44% | 0% | 0% | 0.435 | 1 | 1 | 1 |
| NL | 5L value set | (15) | Moderate low back pain | Large | 0.19 (0.15; 0.24) | -303 (-916; 224) | -1591 | 14% | 86% | 0% | 0% | 0.858 | 1 | 1 | 1 |
|  | 5L to 3L crosswalk |  |  |  | 0.20 (0.17; 0.24) | -303 (-916; 224) | -1486 | 14% | 86% | 0% | 0% | 0.858 | 1 | 1 | 1 |
| US | 5L value set |  |  |  | 0.22 (0.18; 0.27) | -303 (-916; 224) | -1368 | 14% | 86% | 0% | 0% | 0.858 | 1 | 1 | 1 |
|  | 5L to 3L crosswalk |  |  |  | 0.19 (0.16; 0.21) | -303 (-916; 224) | -1630 | 14% | 86% | 0% | 0% | 0.858 | 1 | 1 | 1 |
| JP | 5L value set |  |  |  | 0.16 (0.12; 0.20) | -303 (-916; 224) | -1887 | 14% | 86% | 0% | 0% | 0.858 | 1 | 1 | 1 |
|  | 5L to 3L crosswalk |  |  |  | 0.19 (0.17; 0.21) | -303 (-916; 224) | -1640 | 14% | 86% | 0% | 0% | 0.858 | 1 | 1 | 1 |
| NL | 5L value set | (16) | Severe low back pain | Small | 0.04 (0.001; 0.08) | 178 (-285; 607) | 4422 | 76% | 22% | 0% | 2% | 0.226 | 0.903 | 0.941 | 0.963 |
|  | 5L to 3L crosswalk |  |  |  | 0.03 (-0.002; 0.06) | 178 (-285; 607) | 5750 | 75% | 22% | 0% | 3% | 0.226 | 0.861 | 0.911 | 0.944 |
| US | 5L value set |  |  |  | 0.03 (-0.01; 0.07) | 178 (-285; 607) | 6404 | 71% | 21% | 2% | 6% | 0.226 | 0.793 | 0.849 | 0.883 |
|  | 5L to 3L crosswalk |  |  |  | 0.02 (-0.004; 0.05) | 178 (-285; 607) | 8413 | 73% | 22% | 1% | 4% | 0.226 | 0.766 | 0.838 | 0.898 |
| JP | 5L value set |  |  |  | 0.02 (-0.01; 0.06) | 178 (-285; 607) | 7616 | 71% | 21% | 2% | 6% | 0.226 | 0.761 | 0.831 | 0.874 |
|  | 5L to 3L crosswalk |  |  |  | 0.02 (-0.004; 0.04) | 178 (-285; 607) | 10327 | 73% | 21% | 2% | 4% | 0.226 | 0.714 | 0.806 | 0.875 |
| NL | 5L value set | (17) | Severe low back pain | Medium | 0.11 (0.07; 0.15) | 479 (-13; 939) | 4338 | 97% | 3% | 0% | 0% | 0.025 | 0.999 | 1 | 1 |
|  | 5L to 3L crosswalk |  |  |  | 0.09 (0.06; 0.13) | 479 (-13; 939) | 5062 | 97% | 3% | 0% | 0% | 0.025 | 0.999 | 1 | 1 |
| US | 5L value set |  |  |  | 0.10 (0.07; 0.14) | 479 (-13; 939) | 4701 | 97% | 3% | 0% | 0% | 0.025 | 0.998 | 0.999 | 1 |
|  | 5L to 3L crosswalk |  |  |  | 0.07 (0.05; 0.10) | 479 (-13; 939) | 6442 | 97% | 3% | 0% | 0% | 0.025 | 0.995 | 0.999 | 1 |
| JP | 5L value set |  |  |  | 0.08 (0.05; 0.11) | 479 (-13; 939) | 6306 | 97% | 3% | 0% | 0% | 0.025 | 0.992 | 0.999 | 1 |
|  | 5L to 3L crosswalk |  |  |  | 0.06 (0.04; 0.08) | 479 (-13; 939) | 7621 | 97% | 3% | 0% | 0% | 0.025 | 0.985 | 0.999 | 1 |
| NL | 5L value set | (18) | Severe low back pain | Large | 0.16 (0.13; 0.20) | 249 (-161; 748) | 1525 | 87% | 13% | 0% | 0% | 0.125 | 1 | 1 | 1 |
|  | 5L to 3L crosswalk |  |  |  | 0.15 (0.12; 0.18) | 249 (-161; 748) | 1693 | 87% | 13% | 0% | 0% | 0.125 | 1 | 1 | 1 |
| US | 5L value set |  |  |  | 0.16 (0.12; 0.20) | 249 (-161; 748) | 1554 | 87% | 13% | 0% | 0% | 0.125 | 1 | 1 | 1 |
|  | 5L to 3L crosswalk |  |  |  | 0.12 (0.10; 0.15) | 249 (-161; 748) | 2040 | 87% | 13% | 0% | 0% | 0.125 | 1 | 1 | 1 |
| JP | 5L value set |  |  |  | 0.12 (0.09; 0.15) | 249 (-161; 748) | 2079 | 87% | 13% | 0% | 0% | 0.125 | 1 | 1 | 1 |
|  | 5L to 3L crosswalk |  |  |  | 0.11 (0.09; 0.13) | 249 (-161; 748) | 2305 | 87% | 13% | 0% | 0% | 0.125 | 1 | 1 | 1 |

**Cont. Supplementary Table 4.2. Cost-utility analysis results for 5L value set and 5L to 3L crosswalk per country**

| **Country** | **Scoring method** | **Scenario** | **Patient population** | **Effect size** | **Incremental QALYs**  **IQ (95% CI)** | **Incremental costs**  **€ (95% CI)** | **ICER**  **€/point** | **Distribution CE-plane (%)** | | | | **Probability of cost-effectiveness** | | | |
| --- | --- | --- | --- | --- | --- | --- | --- | --- | --- | --- | --- | --- | --- | --- | --- |
|  |  |  |  |  |  |  |  | **NE** | **SE** | **SW** | **NW** | **p_CE_(0)** | **p_CE_(20000)** | **p_CE_(30000)** | **p_CE_(50000)** |
| NL | 5L value set | (19) | Mild osteoarthritis | Small | 0.03 (-0.01; 0.06) | 82 (-401; 623) | 2900 | 58% | 36% | 1% | 5% | 0.372 | 0.850 | 0.892 | 0.916 |
|  | 5L to 3L crosswalk |  |  |  | 0.03 (-0.01; 0.06) | 82 (-401; 623) | 2719 | 60% | 36% | 1% | 3% | 0.372 | 0.883 | 0.924 | 0.943 |
| US | 5L value set |  |  |  | 0.04 (0.001; 0.08) | 82 (-401; 623) | 1945 | 61% | 37% | 1% | 1% | 0.372 | 0.937 | 0.957 | 0.971 |
|  | 5L to 3L crosswalk |  |  |  | 0.03 (0.001; 0.05) | 82 (-401; 623) | 2925 | 61% | 37% | 1% | 1% | 0.372 | 0.893 | 0.941 | 0.963 |
| JP | 5L value set |  |  |  | 0.02 (-0.01; 0.05) | 82 (-401; 623) | 3529 | 58% | 35% | 2% | 5% | 0.372 | 0.821 | 0.861 | 0.896 |
|  | 5L to 3L crosswalk |  |  |  | 0.02 (-0.002; 0.04) | 82 (-401; 623) | 4173 | 60% | 36% | 1% | 3% | 0.372 | 0.820 | 0.879 | 0.918 |
| NL | 5L value set | (20) | Mild osteoarthritis | Medium | 0.10 (0.06; 0.13) | -125 (-629; 390) | -1299 | 32% | 68% | 0% | 0% | 0.677 | 1 | 1 | 1 |
|  | 5L to 3L crosswalk |  |  |  | 0.11 (0.08; 0.14) | -125 (-629; 390) | -1156 | 32% | 68% | 0% | 0% | 0.677 | 1 | 1 | 1 |
| US | 5L value set |  |  |  | 0.10 (0.07; 0.14) | -125 (-629; 390) | -1198 | 32% | 68% | 0% | 0% | 0.677 | 1 | 1 | 1 |
|  | 5L to 3L crosswalk |  |  |  | 0.09 (0.06; 0.11) | -125 (-629; 390) | -1406 | 32% | 68% | 0% | 0% | 0.677 | 1 | 1 | 1 |
| JP | 5L value set |  |  |  | 0.07 (0.04; 0.10) | -125 (-629; 390) | -1798 | 32% | 68% | 0% | 0% | 0.677 | 1 | 1 | 1 |
|  | 5L to 3L crosswalk |  |  |  | 0.07 (0.05; 0.09) | -125 (-629; 390) | -1781 | 32% | 68% | 0% | 0% | 0.677 | 1 | 1 | 1 |
| NL | 5L value set | (21) | Mild osteoarthritis | Large | 0.14 (0.12; 0.17) | -270 (-708; 179) | -1883 | 11% | 89% | 0% | 0% | 0.887 | 1 | 1 | 1 |
|  | 5L to 3L crosswalk |  |  |  | 0.16 (0.13; 0.18) | -270 (-708; 179) | -1741 | 11% | 89% | 0% | 0% | 0.887 | 1 | 1 | 1 |
| US | 5L value set |  |  |  | 0.15 (0.11; 0.18) | -270 (-708; 179) | -1853 | 11% | 89% | 0% | 0% | 0.887 | 1 | 1 | 1 |
|  | 5L to 3L crosswalk |  |  |  | 0.13 (0.11; 0.15) | -270 (-708; 179) | -2115 | 11% | 89% | 0% | 0% | 0.887 | 1 | 1 | 1 |
| JP | 5L value set |  |  |  | 0.11 (0.08; 0.13) | -270 (-708; 179) | -2574 | 11% | 89% | 0% | 0% | 0.887 | 1 | 1 | 1 |
|  | 5L to 3L crosswalk |  |  |  | 0.11 (0.09; 0.13) | -270 (-708; 179) | -2467 | 11% | 89% | 0% | 0% | 0.887 | 1 | 1 | 1 |
| NL | 5L value set | (22) | Moderate osteoarthritis | Small | 0.03 (-0.01; 0.06) | -175 (-604; 296) | -6847 | 21% | 72% | 5% | 2% | 0.768 | 0.946 | 0.950 | 0.946 |
|  | 5L to 3L crosswalk |  |  |  | 0.02 (-0.01; 0.05) | -175 (-604; 296) | -8488 | 21% | 69% | 8% | 2% | 0.768 | 0.919 | 0.921 | 0.918 |
| US | 5L value set |  |  |  | 0.03 (-0.01; 0.06) | -175 (-604; 296) | -6160 | 21% | 73% | 4% | 2% | 0.768 | 0.953 | 0.954 | 0.955 |
|  | 5L to 3L crosswalk |  |  |  | 0.01 (-0.01; 0.04) | -175 (-604; 296) | -12052 | 20% | 67% | 10% | 3% | 0.768 | 0.893 | 0.898 | 0.895 |
| JP | 5L value set |  |  |  | 0.03 (-0.001; 0.05) | -175 (-604; 296) | -6939 | 22% | 75% | 2% | 1% | 0.768 | 0.965 | 0.972 | 0.974 |
|  | 5L to 3L crosswalk |  |  |  | 0.01 (-0.01; 0.04) | -175 (-604; 296) | -12450 | 20% | 69% | 8% | 3% | 0.768 | 0.906 | 0.915 | 0.914 |
| NL | 5L value set | (23) | Moderate osteoarthritis | Medium | 0.08 (0.05; 0.11) | 11 (-484; 634) | 134 | 49% | 51% | 0% | 0% | 0.506 | 1 | 1 | 1 |
|  | 5L to 3L crosswalk |  |  |  | 0.09 (0.06; 0.12) | 11 (-484; 634) | 120 | 49% | 51% | 0% | 0% | 0.506 | 1 | 1 | 1 |
| US | 5L value set |  |  |  | 0.09 (0.05; 0.12) | 11 (-484; 634) | 125 | 49% | 51% | 0% | 0% | 0.506 | 1 | 1 | 1 |
|  | 5L to 3L crosswalk |  |  |  | 0.07 (0.05; 0.09) | 11 (-484; 634) | 154 | 49% | 51% | 0% | 0% | 0.506 | 1 | 1 | 1 |
| JP | 5L value set |  |  |  | 0.06 (0.03; 0.08) | 11 (-484; 634) | 180 | 49% | 51% | 0% | 0% | 0.506 | 0.998 | 1 | 1 |
|  | 5L to 3L crosswalk |  |  |  | 0.05 (0.03; 0.07) | 11 (-484; 634) | 203 | 49% | 51% | 0% | 0% | 0.506 | 0.999 | 1 | 1 |
| NL | 5L value set | (24) | Moderate osteoarthritis | Large | 0.13 (0.10; 0.15) | -269 (-781; 259) | -2144 | 16% | 84% | 0% | 0% | 0.836 | 1 | 1 | 1 |
|  | 5L to 3L crosswalk |  |  |  | 0.14 (0.11; 0.17) | -269 (-781; 259) | -1938 | 16% | 84% | 0% | 0% | 0.836 | 1 | 1 | 1 |
| US | 5L value set |  |  |  | 0.14 (0.11; 0.17) | -269 (-781; 259) | -1965 | 16% | 84% | 0% | 0% | 0.836 | 1 | 1 | 1 |
|  | 5L to 3L crosswalk |  |  |  | 0.11 (0.09; 0.14) | -269 (-781; 259) | -2355 | 16% | 84% | 0% | 0% | 0.836 | 1 | 1 | 1 |
| JP | 5L value set |  |  |  | 0.10 (0.07; 0.12) | -269 (-781; 259) | -2731 | 16% | 84% | 0% | 0% | 0.836 | 1 | 1 | 1 |
|  | 5L to 3L crosswalk |  |  |  | 0.09 (0.07; 0.11) | -269 (-781; 259) | -2896 | 16% | 84% | 0% | 0% | 0.836 | 1 | 1 | 1 |
| NL | 5L value set | (25) | Severe osteoarthritis | Small | 0.04 (-0.01; 0.09) | 386 (-89; 893) | 10222 | 87% | 6% | 0% | 7% | 0.063 | 0.712 | 0.798 | 0.862 |
|  | 5L to 3L crosswalk |  |  |  | 0.03 (-0.01; 0.08) | 386 (-89; 893) | 11157 | 87% | 6% | 0% | 7% | 0.063 | 0.696 | 0.799 | 0.861 |
| US | 5L value set |  |  |  | 0.05 (-0.004; 0.11) | 386 (-89; 893) | 7693 | 90% | 6% | 0% | 4% | 0.063 | **0.820** | 0.890 | 0.929 |
|  | 5L to 3L crosswalk |  |  |  | 0.04 (-0.001; 0.08) | 386 (-89; 893) | 10566 | 91% | 6% | 0% | 3% | 0.063 | **0.746** | 0.861 | 0.927 |
| JP | 5L value set |  |  |  | 0.04 (-0.01; 0.09) | 386 (-89; 893) | 10189 | 88% | 6% | 0% | 6% | 0.063 | 0.727 | 0.815 | 0.879 |
|  | 5L to 3L crosswalk |  |  |  | 0.03 (0.005; 0.07) | 386 (-89; 893) | 11441 | 92% | 6% | 0% | 2% | 0.063 | 0.751 | 0.874 | 0.939 |
| NL | 5L value set | (26) | Severe osteoarthritis | Medium | 0.14 (0.09; 0.18) | -171 (-645; 318) | -1228 | 23% | 77% | 0% | 0% | 0.772 | 1 | 1 | 1 |
|  | 5L to 3L crosswalk |  |  |  | 0.15 (0.10; 0.19) | -171 (-645; 318) | -1177 | 23% | 77% | 0% | 0% | 0.772 | 1 | 1 | 1 |
| US | 5L value set |  |  |  | 0.15 (0.10; 0.20) | -171 (-645; 318) | -1135 | 23% | 77% | 0% | 0% | 0.772 | 1 | 1 | 1 |
|  | 5L to 3L crosswalk |  |  |  | 0.12 (0.09; 0.16) | -171 (-645; 318) | -1386 | 23% | 77% | 0% | 0% | 0.772 | 1 | 1 | 1 |
| JP | 5L value set |  |  |  | 0.11 (0.06; 0.16) | -171 (-645; 318) | -1561 | 23% | 77% | 0% | 0% | 0.772 | 1 | 1 | 1 |
|  | 5L to 3L crosswalk |  |  |  | 0.10 (0.07; 0.13) | -171 (-645; 318) | -1658 | 23% | 77% | 0% | 0% | 0.772 | 1 | 1 | 1 |
| NL | 5L value set | (27) | Severe osteoarthritis | Large | 0.21 (0.16; 0.25) | -162 (-631; 297) | -781 | 25% | 75% | 0% | 0% | 0.751 | 1 | 1 | 1 |
|  | 5L to 3L crosswalk |  |  |  | 0.22 (0.18; 0.25) | -162 (-631; 297) | -747 | 25% | 75% | 0% | 0% | 0.751 | 1 | 1 | 1 |
| US | 5L value set |  |  |  | 0.22 (0.17; 0.27) | -162 (-631; 297) | -746 | 25% | 75% | 0% | 0% | 0.751 | 1 | 1 | 1 |
|  | 5L to 3L crosswalk |  |  |  | 0.19 (0.16; 0.22) | -162 (-631; 297) | -844 | 25% | 75% | 0% | 0% | 0.751 | 1 | 1 | 1 |
| JP | 5L value set |  |  |  | 0.17 (0.13; 0.22) | -162 (-631; 297) | -930 | 25% | 75% | 0% | 0% | 0.751 | 1 | 1 | 1 |
|  | 5L to 3L crosswalk |  |  |  | 0.18 (0.16; 0.21) | -162 (-631; 297) | -894 | 25% | 75% | 0% | 0% | 0.751 | 1 | 1 | 1 |

**Cont. Supplementary Table 4.2. Cost-utility analysis results for 5L value set and 5L to 3L crosswalk per country**

| **Country** | **Scoring method** | **Scenario** | **Patient population** | **Effect size** | **Incremental QALYs**  **IQ (95% CI)** | **Incremental costs**  **€ (95% CI)** | **ICER**  **€/point** | **Distribution CE-plane (%)** | | | | **Probability of cost-effectiveness** | | | |
| --- | --- | --- | --- | --- | --- | --- | --- | --- | --- | --- | --- | --- | --- | --- | --- |
|  |  |  |  |  |  |  |  | **NE** | **SE** | **SW** | **NW** | **p_CE_(0)** | **p_CE_(20000)** | **p_CE_(30000)** | **p_CE_(50000)** |
| NL | 5L value set | (28) | Mild cancer | Small | 0.02 (-0.02; 0.05) | 185 (-143; 726) | 16790 | 74% | 9% | 1% | 16% | 0.104 | 0.564 | 0.653 | 0.735 |
|  | 5L to 3L crosswalk |  |  |  | 0.01 (-0.02; 0.05) | 185 (-143; 726) | 21052 | 69% | 9% | 1% | 21% | 0.104 | 0.510 | 0.600 | 0.668 |
| US | 5L value set |  |  |  | 0.02 (-0.02; 0.06) | 185 (-143; 726) | 14746 | 76% | 10% | 1% | 13% | 0.104 | 0.604 | 0.698 | **0.765** |
|  | 5L to 3L crosswalk |  |  |  | 0.01 (-0.02; 0.04) | 185 (-143; 726) | 29126 | 67% | 9% | 2% | 22% | 0.104 | 0.426 | 0.523 | **0.618** |
| JP | 5L value set |  |  |  | 0.02 (-0.01; 0.05) | 185 (-143; 726) | 15272 | 80% | 10% | 0% | 10% | 0.104 | 0.605 | **0.719** | 0.807 |
|  | 5L to 3L crosswalk |  |  |  | 0.02 (-0.004; 0.05) | 185 (-143; 726) | 11749 | 86% | 10% | 0% | 39% | 0.104 | 0.722 | **0.830** | 0.902 |
| NL | 5L value set | (29) | Mild cancer | Medium | 0.09 (0.05; 0.12) | -182 (-634; 316) | -2049 | 21% | 79% | 0% | 0% | 0.787 | 1 | 1 | 1 |
|  | 5L to 3L crosswalk |  |  |  | 0.08 (0.05; 0.12) | -182 (-634; 316) | -2192 | 21% | 79% | 0% | 0% | 0.787 | 1 | 1 | 1 |
| US | 5L value set |  |  |  | 0.09 (0.06; 0.13) | -182 (-634; 316) | -1921 | 21% | 79% | 0% | 0% | 0.787 | 1 | 1 | 1 |
|  | 5L to 3L crosswalk |  |  |  | 0.07 (0.04; 0.09) | -182 (-634; 316) | -2748 | 21% | 79% | 0% | 0% | 0.787 | 1 | 1 | 1 |
| JP | 5L value set |  |  |  | 0.07 (0.04; 0.10) | -182 (-634; 316) | -2666 | 21% | 79% | 0% | 0% | 0.787 | 1 | 1 | 1 |
|  | 5L to 3L crosswalk |  |  |  | 0.07 (0.04; 0.09) | -182 (-634; 316) | -2732 | 21% | 79% | 0% | 0% | 0.787 | 1 | 1 | 1 |
| NL | 5L value set | (30) | Mild cancer | Large | 0.15 (0.12; 0.18) | 29 (-492; 538) | 189 | 55% | 45% | 0% | 0% | 0.455 | 1 | 1 | 1 |
|  | 5L to 3L crosswalk |  |  |  | 0.15 (0.12; 0.18) | 29 (-492; 538) | 194 | 55% | 45% | 0% | 0% | 0.455 | 1 | 1 | 1 |
| US | 5L value set |  |  |  | 0.15 (0.12; 0.19) | 29 (-492; 538) | 187 | 55% | 45% | 0% | 0% | 0.455 | 1 | 1 | 1 |
|  | 5L to 3L crosswalk |  |  |  | 0.11 (0.09; 0.14) | 29 (-492; 538) | 254 | 55% | 45% | 0% | 0% | 0.455 | 1 | 1 | 1 |
| JP | 5L value set |  |  |  | 0.12 (0.09; 0.15) | 29 (-492; 538) | 246 | 55% | 45% | 0% | 0% | 0.455 | 1 | 1 | 1 |
|  | 5L to 3L crosswalk |  |  |  | 0.12 (0.09; 0.14) | 29 (-492; 538) | 247 | 55% | 45% | 0% | 0% | 0.455 | 1 | 1 | 1 |
| NL | 5L value set | (31) | Moderate cancer | Small | 0.05 (-0.001; 0.11) | 345 (-190; 916) | 6643 | 86% | 10% | 1% | 3% | 0.102 | 0.847 | 0.903 | 0.928 |
|  | 5L to 3L crosswalk |  |  |  | 0.07 (0.02; 0.12) | 345 (-190; 916) | 4897 | 89% | 10% | 0% | 1% | 0.102 | 0.955 | 0.980 | 0.990 |
| US | 5L value set |  |  |  | 0.06 (0.005; 0.12) | 345 (-190; 916) | 5689 | 87% | 10% | 1% | 2% | 0.102 | 0.888 | 0.925 | 0.948 |
|  | 5L to 3L crosswalk |  |  |  | 0.05 (0.01; 0.09) | 345 (-190; 916) | 7488 | 88% | 10% | 0% | 2% | 0.102 | 0.855 | 0.928 | 0.958 |
| JP | 5L value set |  |  |  | 0.06 (0.01; 0.12) | 345 (-190; 916) | 5764 | 88% | 10% | 1% | 1% | 0.102 | **0.895** | **0.936** | **0.960** |
|  | 5L to 3L crosswalk |  |  |  | 0.005 (-0.03; 0.04) | 345 (-190; 916) | 72291 | 52% | 6% | 4% | 38% | 0.102 | **0.270** | **0.349** | **0.425** |
| NL | 5L value set | (32) | Moderate cancer | Medium | 0.11 (0.07; 0.14) | -260 (-696; 178) | -2423 | 13% | 87% | 0% | 0% | 0.869 | 1 | 1 | 1 |
|  | 5L to 3L crosswalk |  |  |  | 0.12 (0.09; 0.15) | -260 (-696; 178) | -2129 | 13% | 87% | 0% | 0% | 0.869 | 1 | 1 | 1 |
| US | 5L value set |  |  |  | 0.12 (0.08; 0.15) | -260 (-696; 178) | -2213 | 13% | 87% | 0% | 0% | 0.869 | 1 | 1 | 1 |
|  | 5L to 3L crosswalk |  |  |  | 0.11 (0.08; 0.13) | -260 (-696; 178) | -2433 | 13% | 87% | 0% | 0% | 0.869 | 1 | 1 | 1 |
| JP | 5L value set |  |  |  | 0.08 (0.04; 0.11) | -260 (-696; 178) | -3217 | 13% | 87% | 0% | 0% | 0.869 | 1 | 1 | 1 |
|  | 5L to 3L crosswalk |  |  |  | 0.11 (0.09; 0.13) | -260 (-696; 178) | -2358 | 13% | 87% | 0% | 0% | 0.869 | 1 | 1 | 1 |
| NL | 5L value set | (33) | Moderate cancer | Large | 0.30 (0.26; 0.34) | -236 (-724; 197) | -778 | 15% | 85% | 0% | 0% | 0.848 | 1 | 1 | 1 |
|  | 5L to 3L crosswalk |  |  |  | 0.27 (0.24; 0.30) | -236 (-724; 197) | -874 | 15% | 85% | 0% | 0% | 0.848 | 1 | 1 | 1 |
| US | 5L value set |  |  |  | 0.27 (0.22; 0.31) | -236 (-724; 197) | -889 | 15% | 85% | 0% | 0% | 0.848 | 1 | 1 | 1 |
|  | 5L to 3L crosswalk |  |  |  | 0.19 (0.17; 0.21) | -236 (-724; 197) | -1243 | 15% | 85% | 0% | 0% | 0.848 | 1 | 1 | 1 |
| JP | 5L value set |  |  |  | 0.14 (0.10; 0.18) | -236 (-724; 197) | -1682 | 15% | 85% | 0% | 0% | 0.848 | 1 | 1 | 1 |
|  | 5L to 3L crosswalk |  |  |  | 0.14 (0.12; 0.17) | -236 (-724; 197) | -1665 | 15% | 85% | 0% | 0% | 0.848 | 1 | 1 | 1 |
| NL | 5L value set | (34) | Severe cancer | Small | 0.04 (-0.02; 0.10) | 307 (-190; 832) | 7474 | 79% | 11% | 1% | 9% | 0.118 | 0.772 | 0.824 | 0.860 |
|  | 5L to 3L crosswalk |  |  |  | 0.03 (-0.02; 0.09) | 307 (-190; 832) | 9040 | 78% | 11% | 1% | 10% | 0.118 | 0.722 | 0.786 | 0.835 |
| US | 5L value set |  |  |  | 0.05 (-0.01; 0.12) | 307 (-190; 832) | 6039 | 82% | 12% | 0% | 6% | 0.118 | **0.838** | **0.877** | 0.906 |
|  | 5L to 3L crosswalk |  |  |  | 0.02 (-0.01; 0.07) | 307 (-190; 832) | 12906 | 76% | 11% | 1% | 12% | 0.118 | **0.623** | **0.713** | 0.784 |
| JP | 5L value set |  |  |  | 0.04 (-0.01; 0.11) | 307 (-190; 832) | 6839 | 81% | 12% | 0% | 7% | 0.118 | **0.815** | 0.853 | 0.887 |
|  | 5L to 3L crosswalk |  |  |  | 0.03 (-0.01; 0.06) | 307 (-190; 832) | 11741 | 80% | 11% | 0% | 8% | 0.118 | **0.674** | 0.768 | 0.846 |
| NL | 5L value set | (35) | Severe cancer | Medium | 0.18 (0.12; 0.24) | 386 (-38; 854) | 2171 | 95% | 5% | 0% | 0% | 0.045 | 1 | 1 | 1 |
|  | 5L to 3L crosswalk |  |  |  | 0.17 (0.12; 0.22) | 386 (-38; 854) | 2214 | 95% | 5% | 0% | 0% | 0.045 | 1 | 1 | 1 |
| US | 5L value set |  |  |  | 0.19 (0.13; 0.25) | 386 (-38; 854) | 2032 | 95% | 5% | 0% | 0% | 0.045 | 1 | 1 | 1 |
|  | 5L to 3L crosswalk |  |  |  | 0.14 (0.1; 0.18) | 386 (-38; 854) | 2711 | 95% | 5% | 0% | 0% | 0.045 | 1 | 1 | 1 |
| JP | 5L value set |  |  |  | 0.14 (0.08; 0.2) | 386 (-38; 854) | 2686 | 95% | 5% | 0% | 0% | 0.045 | 1 | 1 | 1 |
|  | 5L to 3L crosswalk |  |  |  | 0.12 (0.09; 0.16) | 386 (-38; 854) | 3173 | 95% | 5% | 0% | 0% | 0.045 | 1 | 1 | 1 |
| NL | 5L value set | (36) | Severe cancer | Large | 0.27 (0.21; 0.33) | -162 (-640; 305) | -595 | 24% | 76% | 0% | 0% | 0.756 | 1 | 1 | 1 |
|  | 5L to 3L crosswalk |  |  |  | 0.29 (0.24; 0.34) | -162 (-640; 305) | -551 | 24% | 76% | 0% | 0% | 0.756 | 1 | 1 | 1 |
| US | 5L value set |  |  |  | 0.30 (0.24; 0.36) | -162 (-640; 305) | -5360. | 24% | 76% | 0% | 0% | 0.756 | 1 | 1 | 1 |
|  | 5L to 3L crosswalk |  |  |  | 0.27 (0.23; 0.31) | -162 (-640; 305) | -604 | 24% | 76% | 0% | 0% | 0.756 | 1 | 1 | 1 |
| JP | 5L value set |  |  |  | 0.24 (0.18; 0.30) | -162 (-640; 305) | -681 | 24% | 76% | 0% | 0% | 0.756 | 1 | 1 | 1 |
|  | 5L to 3L crosswalk |  |  |  | 0.25 (0.21; 0.29) | -162 (-640; 305) | -647 | 24% | 76% | 0% | 0% | 0.756 | 1 | 1 | 1 |

NL: the Netherlands. US: United States. JP: Japan. QALY: quality-adjusted life-year. IQ: incremental QALY. 95% CI: 95% confidence interval. ICER: Incremental Cost-Effectiveness Ratio. NE: northeast. SE: southeast. SW: southwest. NW: orthwest. p_CE_: probability of the intervention being cost-effective compared to control.
